# Computational Tool for Fast *in silico* Evaluation of *h*ERG K^+^ Channel Affinity

**DOI:** 10.3389/fchem.2017.00007

**Published:** 2017-02-23

**Authors:** Giulia Chemi, Sandra Gemma, Giuseppe Campiani, Simone Brogi, Stefania Butini, Margherita Brindisi

**Affiliations:** ^1^European Research Centre for Drug Discovery (NatSynDrugs), University of SienaSiena, Italy; ^2^Department of Biotechnology, Chemistry and Pharmacy, University of SienaSiena, Italy

**Keywords:** 3D-QSAR, pharmacophore modeling, ligand-based model, human *Ether-à-go-go*-related gene (*h*ERG), cardiotoxicity

## Abstract

The development of a novel comprehensive approach for the prediction of *h*ERG activity is herein presented. Software Phase has been used to derive a 3D-QSAR model, employing as alignment rule a common pharmacophore built on a subset of 22 highly active compounds (threshold *Ki*: 50 nM) against *h*ERG K^+^ channel. Five features comprised the pharmacophore: two aromatic rings (R_1_ and R_2_), one hydrogen-bond acceptor (A), one hydrophobic site (H), and one positive ionizable function (P). The sequential 3D-QSAR model developed with a set of 421 compounds (randomly divided in training and test set) yielded a test set (*Q*^2^) = 0.802 and proved to be predictive with respect to an external test set of 309 compounds that were not used to generate the model ($r_{\text{ext}\text{\_}\text{ts}}^{2}$ = 0.860). Furthermore, the model was submitted to an *in silico* validation for assessing the reliability of the approach, by applying a decoys set, evaluating the Güner and Henry score (*GH*) and the Enrichment Factor (*EF*), and by using the ROC curve analysis. The outcome demonstrated the high predictive power of the inclusive 3D-QSAR model developed for the *h*ERG K^+^ channel blockers, confirming the fundamental validity of the chosen approach for obtaining a fast proprietary cardiotoxicity predictive tool to be employed for rationally designing compounds with reduced *h*ERG K^+^ channel activity at the early steps of the drug discovery trajectory.

## Introduction

The *h*ERG K^+^ channel is a member of the *Ether-à-go-go* family encoded by *KCNH2* gene that consists of four identical subunits (Trudeau et al., 1995). This channel is well known for its important role in the electrical activity of the heart that coordinates heart's beating. Mutations in *KCHN2* gene lead to long-QT syndrome, which can lead to ventricular arrhythmia or other adverse effects on cardiovascular system, causing sudden death (Sanguinetti et al., 1995; De Bruin et al., 2005; Sanguinetti and Tristani-Firouzi, 2006). *h*ERG K^+^ channels are particularly sensitive to blockage by a large number of structurally diverse drugs and are critical antitargets in drug discovery process (Dumaine and Kirsch, 1998; Dumaine et al., 1998; Barbey et al., 2002; Redfern et al., 2003; Thomas et al., 2003a,b; Rajamani et al., 2006). The interaction of small molecules with *h*ERG K^+^ channel is one of the major issues encountered by the pharmaceutical companies related to the drug development process. Blockade of *h*ERG K^+^ channel has then become a severe limitation for the introduction of new drugs in the market. Moreover, in the recent years several blockbuster drugs including astemizole, droperidol, terfenadine, lidolazine, sertindole, cisapride, and chlorpromazine have been discontinued due to their relevant activity on *h*ERG K^+^ channel (Honig et al., 1993; Mohammad et al., 1997; Gottlieb, 1999; Barbey et al., 2002; Ray et al., 2004; Roden, 2004). Currently, pharmaceutical companies may account on high throughput methods for predicting the capability of compounds to interfere with *h*ERG K^+^ channels, thus identifying safer compounds at the early stage of the drug discovery process. However, the inherent costs associated to this screening procedure are dramatically elevated in terms of expenditure, amount of molecules consumed, and time of analysis. Accordingly, the development of a fast and reliable computational procedure for an early and trustworthy prediction of *h*ERG K^+^ channel interference would represent a useful and significant advance in this frame. Recently, for this purpose diverse ligand-based computational models have been developed (see ref Keserü, 2003; Aronov, 2005; Wang et al., 2013, 2016; Braga et al., 2015 for further details). Moreover, three dimensional (3D) channel models using homology modeling technique were generated and adopted in molecular docking/molecular dynamics simulations for an *in silico* assessment of potential affinity of compounds for *h*ERG K^+^ channel (Farid et al., 2006; Boukharta et al., 2011; Durdagi et al., 2011, 2012; Dempsey et al., 2014). This latter approach was successfully applied by us in the past for investigating *h*ERG affinity of a class of antimalarials (Gemma et al., 2012) and antipsychotics agents (Butini et al., 2009, 2010; Brindisi et al., 2014). However, despite its accuracy, this approach is extremely time consuming. So, adopting a protocol based on homology model, molecular docking, and molecular dynamics is a challenging task for studying a large number of compounds. To improve accuracy of investigation of large set of molecules, we have developed and validated an inclusive 3D-QSAR model aimed at obtaining a fast predictive *in silico* tool able to unveil potential *h*ERG K^+^ channel activity of structurally diverse blockers at the early stages of our drug discovery and development process. This tool could be of pivotal importance for assisting us and others in designing active compounds for a given target endowed with limited *h*ERG K^+^ channel affinity.

## Results and discussion

**Figure 1:**
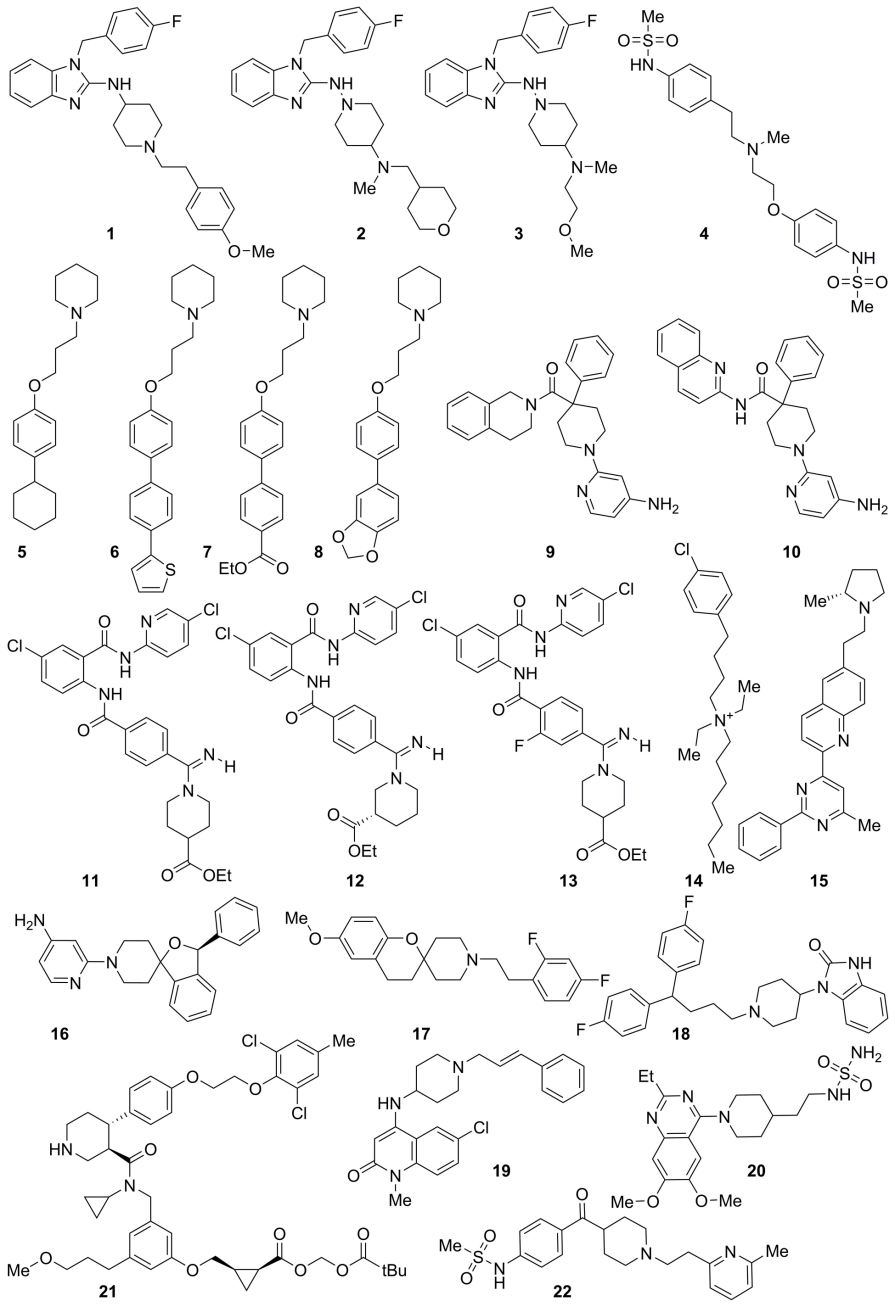
**Structure of highly active compounds against ***h***ERG K^**+**^ channel used for generating a common features pharmacophore**.

**Figure 2:**
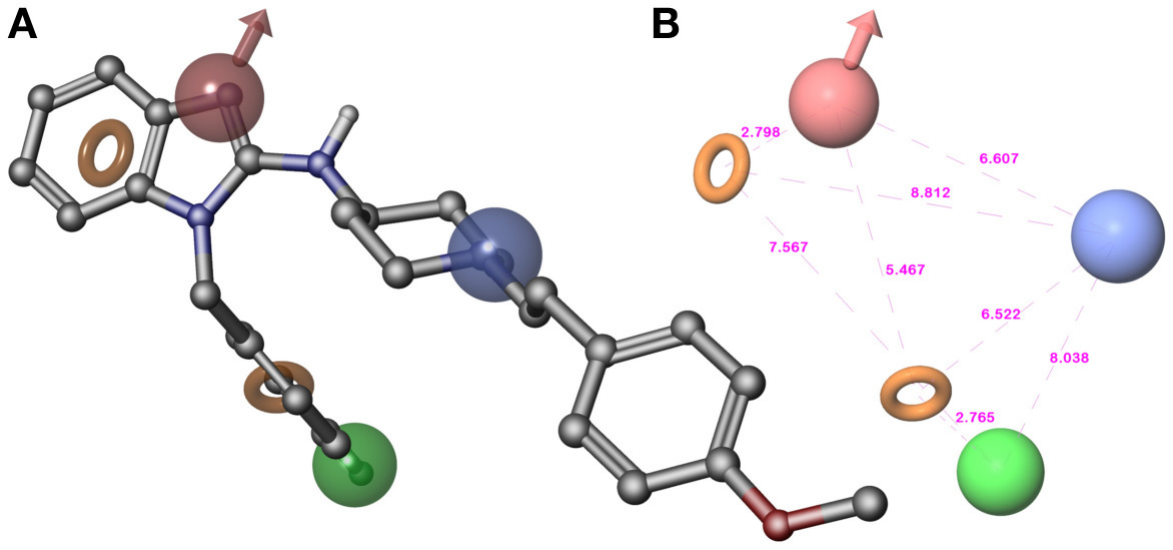
**(A)** Superposition of highly active compound **1** (astemizole) and AHPRR hypothesis. **(B)** AHPRR hypothesis and its inter-feature distances. Features are as follows: H-bond acceptors = A, red vector; hydrophobic feature = H, green sphere; positive ionizable = P, blue sphere; aromatic feature = R_1_ and R_2_, orange rings (the pictures were generated by means of Maestro software).

We have recently reported a series of predictive 3D-QSAR studies for different targets, in which a Phase common features pharmacophore has been used as the alignment rule for deriving quantitative structure-activity relationship models. These latter were used in virtual screening protocols and for rationally designing a library of compounds active on a given target (Brogi et al., 2011, 2013, 2015, 2016; Castelli et al., 2012; Pasquini et al., 2012; Zaccagnini et al., 2017). The results of the above-mentioned drug discovery investigations inspired us to generate a comprehensive computational model for predicting potential *h*ERG-related cardiotoxicity of compounds during the early steps of the drug discovery process employing the same software. For this purpose, a data set of 421 compounds (see the Supplementary Material for further details and references) with *h*ERG K^+^ channel binding affinity spanning five orders of magnitude (from 3.3 nM of compound **1** to 54 μM of compound **421**, Table S1) and belonging to different structural classes, were used for deriving a predictive *h*ERG 3D-QSAR model. Among them, 22 compounds selected from the literature (**1–22**, Figure 1, Table S1) as potent *h*ERG K^+^ channel blockers such as astemizole (**1**) (Finlayson et al., 2001; Fletcher et al., 2002; Blackburn et al., 2006; Murphy et al., 2006; Zhu et al., 2006; Deacon et al., 2007; Coon et al., 2009; Owen et al., 2009; Patel et al., 2009; Liu et al., 2010; Aspiotis et al., 2011; Levoin et al., 2011) were chosen for developing a common features pharmacophore (Figure 2), subsequently used as alignment rule for generating the 3D-QSAR model. At this step the compounds were randomly divided, 60% in the training and 40% in the test set (Table S1). This choice was made to guarantee the inclusion of the positive information arising from 60% of the compounds included in the training set (corresponding to 253 compounds), for the development of the computational tool. At the same time the high number of compounds kept in the test set (40%, 168 compounds) assures an appropriate evaluation of the predictive power of the generated model by means of an exhaustive internal cross-validation. Remarkably, for reducing the weakness of the ligand-based approach, an extensive conformational analysis for the selected ligands was accomplished by means of MacroModel software (see experimental section for further details). Conformational analysis is relevant for enhancing the quality of the alignment for the compounds used to build the 3D-QSAR model as well as the consistency of the *in silico* tool (Brogi et al., 2011, 2013, 2015; Durdagi et al., 2011; Zaccagnini et al., 2017).

The top-ranked hypothesis (AHPRR.42), obtained by means of Phase, was formed by five features: two aromatic rings (R_1_ and R_2_), one hydrogen-bond acceptor (A), one hydrophobic site (H), and one positive ionizable function (P). Pharmacophore AHPRR (shown in Figure 2A superimposed to astemizole (**1**), its inter-feature distances are reported in Figure 2B) highlighted the structural requirements for interacting with the channel, as confirmed by the comparison between the docked pose of astemizole (**1**) into *h*ERG K^+^ channel homology model (Masetti et al., 2008) with the alignment of **1** onto the pharmacophore model. Moreover, the analysis with molecular determinants reported by Farid and co-workers for a large number of *h*ERG blockers is consistent with our developed computational model (Farid et al., 2006). Accordingly, it could be inferred that pharmacophore AHPRR actually accounts for relevant interactions between selected molecules and *h*ERG channel. Indeed, it is well known that π-π stacking or hydrophobic contacts with key residues of *h*ERG K^+^ channel such as Y652 and/or F656 as well as ionic interactions are commonly found for a large number of *h*ERG blockers. In fact, positively charged functional groups are present in many drugs that strongly bind *h*ERG K^+^ channels. The presence of atoms able to form polar contacts with T623 and S624 represents another important requirement for *h*ERG inhibition. Therefore, the above-mentioned AHPRR hypothesis accounts for the key features, which play a pivotal role for *h*ERG K^+^ channel affinity, when at appropriate reciprocal distances. Consequently, it is not arbitrary to state that matching the pharmacophore may be predictive for binding to the channel. The AHPRR hypothesis was then used to align the molecules for the development of an atom-based 3D-QSAR model. As suggested by the Phase's user manual, the atom-based version of Phase's 3D-QSAR workflow was preferred to the pharmacophore-based one. Such a choice implied considering the whole contributions to *h*ERG channel activity as deriving from all the structural features also including the steric clashes, rather than from only those from the pharmacophore relevant features.

Models containing one to seven partial least squares (PLS) factors were generated, whose statistical parameters are detailed in Table 1. The model featuring seven PLS factors was chosen, since it better performed on the whole than those with fewer PLS factors. We have considered models containing up to seven PLS factors to avoid over-fitting phenomena. The high correlation and cross-validated correlation coefficients (*r*^2^ = 0.911 and *Q*^2^ = 0.802, respectively) of the selected model together with the high Pearson *R*-value (R-Pearson = 0.901), suggested a close correspondence between estimated and experimental *Ki*-values. These parameters are indicative of a computational model characterized by a strong predictive power and significance.

**Table 1:** **3D-QSAR statistical parameters of the seven Phase-derived sets of models**.

**PLS**	**r^2^^a^**	**SD^b^**	**F^c^**	**P^d^**	**RMSE^e^**	**Q^2^^f^**	**R-Pearson^g^**
1	0.179	0.793	54.7	2.13e-12	0.532	0.382	0.647
2	0.387	0.687	78.8	2.94e-27	0.470	0.518	0.738
3	0.574	0.573	112.0	6.40e-46	0.469	0.521	0.763
4	0.737	0.452	173.4	1.31e-70	0.382	0.681	0.829
5	0.831	0.363	242.8	3.65e-93	0.338	0.751	0.868
6	0.872	0.316	279.6	8.18e-107	0.327	0.767	0.883
7	0.911	0.264	357.9	9.14e-125	0.301	0.802	0.901

a *r^2^, value of r^2^ of the regression*.

b *SD, standard deviation of the regression*.

c *F, variance ratio*.

d *P, significance level of variance ratio*.

e *RMSE, root-mean-square error*.

f *Q^2^: value of Q^2^ for the predicted activities*.

g *R: r-Pearson, correlation between the predicted and observed selectivity index values for the test set*.

**Figure 3:**
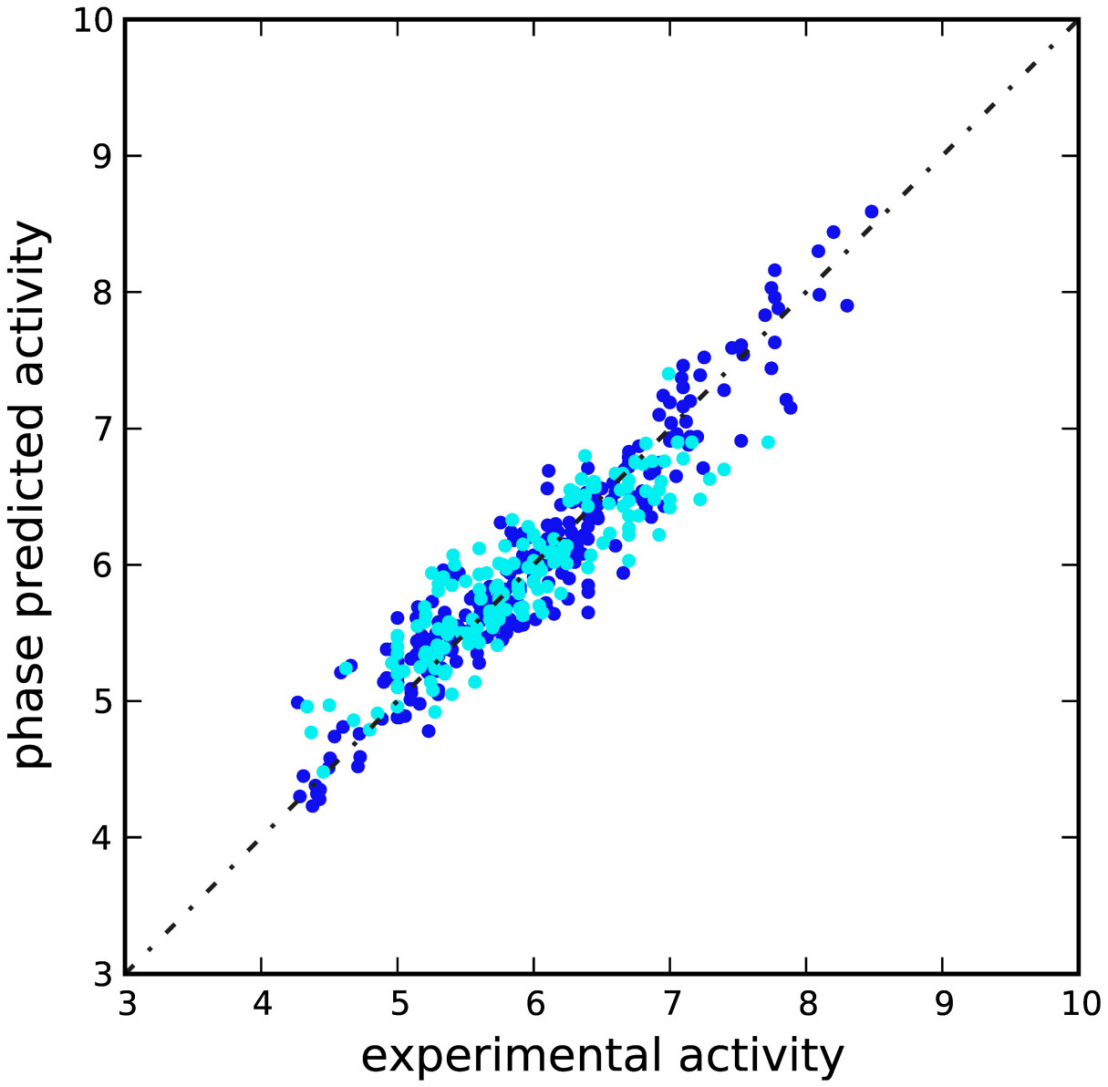
**Scatter plot for the predicted and observed p***Ki*** values (M) as calculated by the 3D-QSAR model applied to the training set (blue) and test set (cyan) compounds**.

A scatter plot of experimental against predicted activities was built to assess the results (Figure 3), which showed that *Ki*-values were effectively estimated for compounds belonging to training and test sets (Table S1). This latter, coupled with the limited number of PLS factors, the small *RMSE*-value, standard deviation (SD), variance ratio (P), and the large *F*-value supported the reliability of the approach.

**Figure 4:**
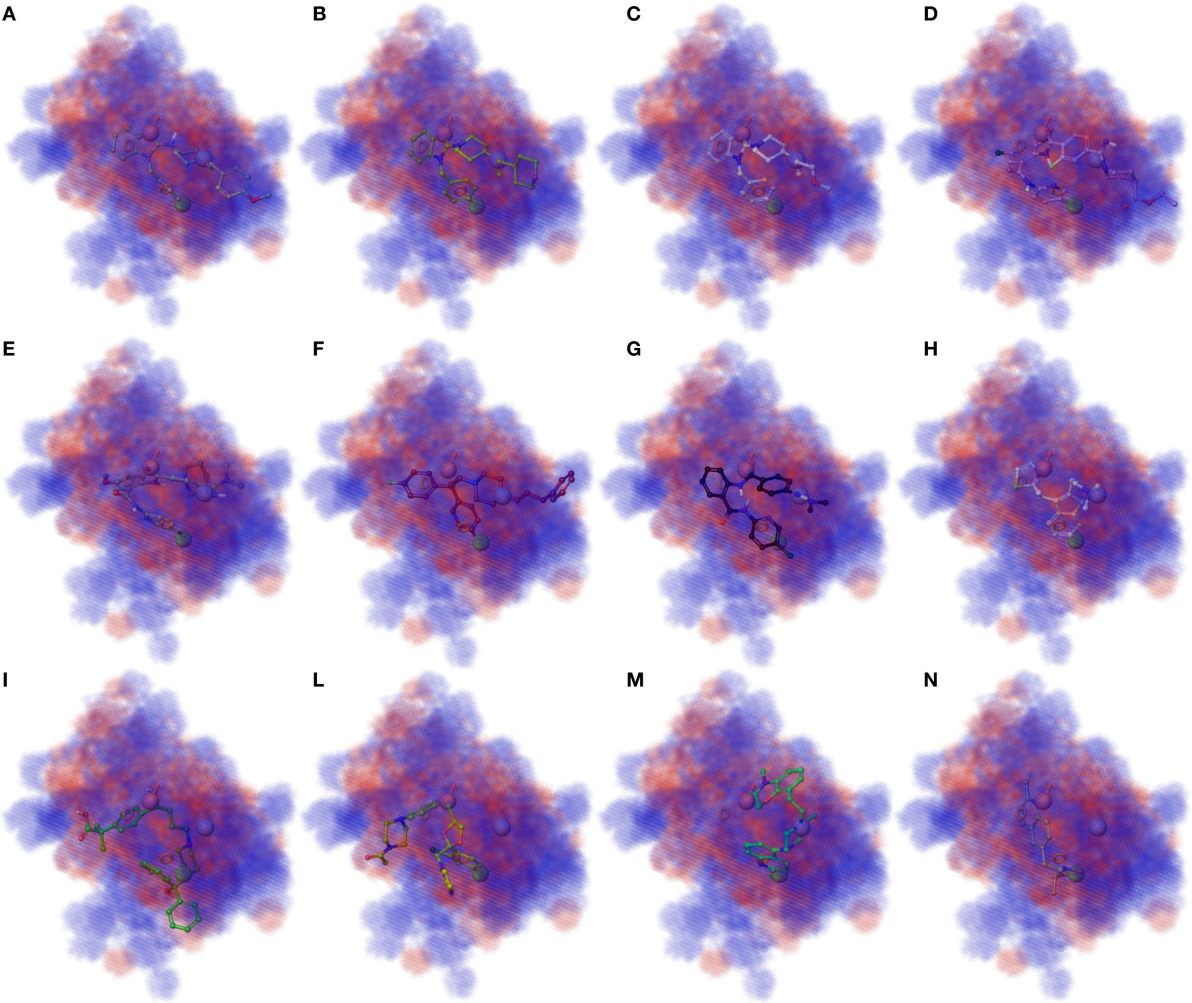
**(A–D)** Superposition of highly active compounds **1** (astemizole), **2**, **3**, and **13** with the 3D-QSAR model. **(E–H)** Superposition of moderate active compounds **35**, **56** (vanoxerine GBR-12909), **398** and **409** with the 3D-QSAR model. **(I–N)** Superposition of less active compounds **54** (fexofenadine), **57** (ketoconazole), **75** and **421** with the 3D-QSAR model. The pictures were generated by means of Maestro software (Schrödinger, LLC, New York, NY, 2015).

3D plots of the crucial volume elements occupied by ligands were employed to visualize the outcome of the 3D-QSAR model. In Figure 4 is depicted the 3D plot representation of the model superimposed to high (**1**, **2**, **3**, and **13**), moderate (**35**, **56**, **398**, and **409**), and less active derivatives (**54**, **57**, **75**, and **421**; Figures 4A–N, respectively). In this illustration, the cubes represent positive (blue cubes) and negative (red cubes) coefficients. For a ligand possessing atoms or functional groups occupying these volumes an increase or a decrease of activity could be predicted. Notably, compound **1** as well as other highly active molecules mainly occupy the blue regions (Figures 4A–D), while the less active compounds such as **421** largely resides on the red regions (Figures 4I–N).

After the generation of the 3D-QSAR model and, in order to perform its theoretical validation, an external test set was selected from the literature. This set was composed of 309 unrelated compounds not used for generating the model, with different inhibitory potency against *h*ERG K^+^ channel (ranging from 0.28 nM to 51 μM; Table S2 in the Supplementary Material). We have included very active compounds in order to stress the model for assessing its actual predictive power. Satisfyingly, the activities of the compounds included in the external test set were satisfactorily estimated by our model (Table S2). In the scatter plot depicted in Figure S1, the experimental and predicted p*Ki*-values of these compounds are also displayed (correlation coefficient $r_{\text{ext}\text{\_}\text{ts}}^{2}$ = 0.860). This outcome provided further indication that the correlation of the model was not accidental.

**Figure 5:**
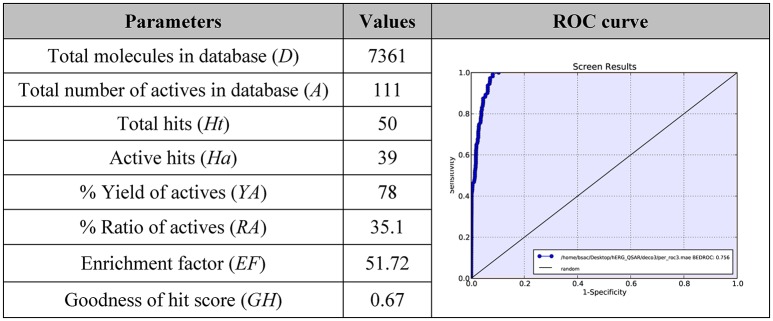
*****EF*** and ***GH*** scores obtained by the application of 3D-QSAR model in a database screening and ROC curve generated from database screening**.

Furthermore, the mentioned model was submitted to a further *in silico* validation by using two different approaches. In order to perform this step, we applied a commonly used validation method based on the generation of decoys set. Starting from highly active compounds used for the model generation (**1**–**22**), other compounds with relevant potency against *h*ERG K^+^ channel (cut-off *Ki* ≤ 150 nM; Table S1) and active molecules from the external test set (cut-off *Ki* ≤ 150 nM**;** Table S2) for a total of 111 actives (Table S3), we generated 7,250 decoys by means of Database of Useful Decoys: Enhanced (DUD-E) server (Huang et al., 2006; Mysinger et al., 2012; see Materials and Methods section for further details about the selection of active compounds). This procedure is largely used to assess the ability of *in silico* tools such as 3D-QSAR models, to discriminate between inactive or active derivatives (Sakkiah et al., 2011; Thangapandian et al., 2011; Braga and Andrade, 2013; Krishna et al., 2014; Brogi et al., 2015, 2016). Based on the obtained results, the assessment clearly demonstrated the validity of the proposed model. The analysis of the results (Figure 5A) of the decoys set revealed a trend where the inactive compounds fail to completely fulfill all the pharmacophore features, thus making their predicted activity very poor or absent. On the contrary, active compounds were reasonably well estimated by the 3D-QSAR model. Notably, we have found 39 actives in the top fifty ranked compounds. Moreover, this qualitative analysis was also supported by Enrichment Factor (*EF*) and Goodness of hit (*GH*) values. The statistical parameters obtained from the model validation were reported in Figure 5A and calculated as reported in the Materials and Methods section.

In particular, a database of 7,361 compounds (*D*) including 111 known active molecules (*A*) was used to early validate our *h*ERG 3D-QSAR model herein discussed. The database screening results (Figure 5A) displayed that 50 molecules were found as hits (*Ht*) (cutoff value corresponding to potential purchasable compounds after a virtual screening protocol). Among *Ht*, 39 compounds (*Ha*) are known *h*ERG blockers (*A*). From screening we calculated an *EF* = 51.72, meaning that it could be about 52 times more probable to find active molecules from chemical-databases with respect to chance. The estimated *GH* score value of 0.67, larger than 0.5, demonstrates a great consistency of the model, suggesting that the presented computational model could be efficiently used for designing compounds with reduced *h*ERG K^+^ channel affinity.

The 3D-QSAR model was evaluated by means of the receiver operating characteristic (ROC) curve, for assessing the balance between model sensitivity and specificity (capability to discover true positives and capability to avoid false positives, respectively; Triballeau et al., 2005; Zhao et al., 2009; Zaccagnini et al., 2017). Compounds included in the validation database (7,361 compounds) were virtually screened by using the generated computational tool and ordered according to their estimated p*Ki*. The output of ROC curve provided a score for assessing the performance of the model. In particular, the closer the ROC score is to 1.0, the better is the model at discriminating active from inactive compounds. The AUC score of our presented *in silico* model was found to be 0.96 (Figure 5B), showing high confidence of the 3D-QSAR model, indicating that the computational tool possessed a rationale for virtual screening, and it could be effectively used to rationally design compounds with reduced *h*ERG activity. Notably the performance of our model appears to be comparable with the previous published models (Aronov, 2005; Wang et al., 2013, 2016; Braga et al., 2015).

## Conclusion

In summary, a pharmacophore hypothesis was built by applying ligand-based pharmacophore generation workflow, implemented in Phase, followed by the development and validation of a 3D-QSAR model, by using PLS analysis for estimating the *h*ERG K^+^ channel activity, using a large set of compounds (training set, test set, and an external test set for a total of 730 molecules). The aim of this approach was to develop a fast and reliable in-house computational tool for the prediction of *h*ERG K^+^ channel activity during the drug discovery process. The computational model developed and validated by us is endowed with strong predictivity, as herein described, and might be useful for the rational design and optimization of new inhibitors against a given target with reduced potential *h*ERG-related cardiotoxicity.

## Materials and methods

### Computational details

#### 3D-QSAR model generation

3D structure building, pharmacophore mapping and 3D-QSAR studies were carried out by means of Maestro 10.1 (Schrödinger, LLC, New York, NY, 2015). Phase 4.2 (Schrödinger, LLC, New York, NY, 2015), implemented in Maestro suite, was used to generate pharmacophore hypotheses and 3D-QSAR model for *h*ERG K^+^ channel. Conformers of each derivative were generated by means of MacroModel 10.7 (Schrödinger, LLC, New York, NY, 2015), using the OPLS_2005 force field (Jorgensen et al., 1996). The solvent effects are simulated employing the analytical Generalized-Born/Surface-Area (GB/SA) model (Still et al., 1990), and no cutoff for nonbonded interactions was selected. Polak-Ribiere conjugate gradient (PRCG) method with 2,000 maximum iterations and 0.001 gradient convergence threshold was employed. The conformational searches were carried out by applying MCMM (Monte Carlo Multiple Minimum) torsional sampling method. Automatic setup with 21 kJ/mol (5.02 kcal/mol) in the energy window for saving structure and a 0.5 Å cutoff distance for redundant conformers was used. Phase was employed to develop common features hypotheses. Pharmacophore feature sites for the compounds were specified by a set of features well-defined in Phase as hydrogen-bond donor (D), hydrogen-bond acceptor (A), aromatic ring (R), positively charged group (P), negatively charged group (N), and hydrophobic group (H). Twenty-two active compounds (Figure 1 and in Table S1 in the Supplementary Material) were selected for generating the pharmacophore hypotheses for *h*ERG K^+^ channel. Common pharmacophore hypotheses were identified, scored and ranked by means of conformational analysis and tree-based partitioning techniques. The best ranked pharmacophore model obtained by Phase [AHPRR, shown in Figure 2 superimposed to astemizole (**1**)], consisted of five features: two aromatic rings (R_1_ and R_2_), one hydrogen-bond acceptor (A), one hydrophobic site (H), and one positive ionizable function (P). This pharmacophore was chosen for further 3D-QSAR analysis. All molecules used for QSAR studies (Table S1) were aligned to the selected pharmacophore hypothesis. In this study, we set a p*Ki* threshold for the selection of active and inactive ligands. Compounds were chosen based on the displacement assay against *h*ERG using the labeled [^3^H]dofetilide. In particular, compounds that showed a *Ki* comprised between 5 and 54 μM were considered as inactive compounds. Moderate inhibitors were considered compounds with *Ki* between 50 nM and 5 μM, while compounds possessing a *Ki* ≤ 50 nM were considered potent inhibitors of *h*ERG K^+^ channel and consequently as active in *h*ERG blockage during 3D-QSAR model generation, a very similar selection was already reported in literature (Durdagi et al., 2011). Notably only molecules with experimentally definite inhibitory potency have been selected to develop the *in silico* model, for avoiding possible faults arising from the inclusion in the set of molecules with uncertain activity. Atom-based QSAR models were generated for *h*ERG hypothesis using the 253 compounds in the training set (421 compounds were randomly divided 60% in the training and 40% in the test set) and a grid spacing of 0.5 Å. QSAR models containing one to seven PLS factors were produced, and an internal cross-validation was achieved employing leave-n-out (LnO) technique as specified in Phase user manual (Phase, version 4.2, User Manual, Schrödinger press, LLC, New York, NY, 2015).

#### 3D-QSAR model validation

Extensive model validation was performed using an external test set of compounds (309 molecules) not used for generating and cross validating the model. Compounds were prepared by using Maestro, LigPrep and MacroModel adopting the same procedure for preparing the molecules used to derive the model.

DUD-E web server (http://dude.docking.org access date November 2016) was employed to generate a set of decoys starting from the active compounds selected to develop the pharmacophore model, other compounds with relevant activity against *h*ERG K^+^ channel and active compounds in the external test set, for a total of 111 active compounds (Table S3). For this set of active ligands, DUD-E server made available 7,250 inactive ligands from a subset of the ZINC database (http://zinc.docking.org access date November 2016) filtered by means of Lipinski rules for drug-likeness, for a total of 7,361 compounds between inactives and actives. Each of these inactive decoys was selected to bear a resemblance to the reference molecule in terms of physico-chemical properties but a divergence from it in 2D structure (e. g. large difference of Tanimoto coefficient between decoys and active compounds). The generated decoys were divided in 145 smiles files, downloaded from the website, imported into Maestro and prepared by means of LigPrep 3.3 (Schrödinger, LLC, New York, NY, 2015) to properly convert smiles into three-dimensional structures. Subsequently, in order to perform a minimization and a conformational search of the obtained structures MacroModel program was adopted (the same parameters reported for ligand preparation were applied). A single file with conformers of active molecules and decoys was produced and submitted to the software for evaluating the activity against *h*ERG K^+^ channel using the 3D-QSAR model employing “search for matches” option. After decoys generation and the prediction of p*Ki*s, the Güner and Henry score, i.e., goodness of hit-list (*GH*) and the enrichment factor (*EF*) value were estimated using the Equations 1, 2, respectively.

(1) $$\begin{array}{c} EF=\frac{Ha/Ht}{\left(A/D\right)} \end{array}$$

(2) $$\begin{array}{c} GH=\left\{\frac{Ha*(3A+Ht)}{4HtA}\right\}*\left[1-\frac{\left(Ht-Ha\right)}{\left(D-A\right)}\right] \end{array}$$

*Ht* represents the total number of compounds in the hit list found by virtual screening, *Ha* is the total actives found by virtual screening considering the top 50-ranked position (positions comprise within the cutoff value). The total number of compounds (*Ht*) might represent the amount of molecules to purchase after a virtual screening protocol and almost the 1% of the considered database (*D*). *A* represents the total of the active derivatives enclosed in the database, and *D* stands for the total number of molecules existing in the set. The *GH* score ranges from 0 to 1. The *GH* score 0 indicates a null model; while the *GH* score 1 denotes an ideal model. Additionally, by using the equations 3 and 4, the % yield of actives (*%YA*) and % ratio of actives (*%RA*) were calculated, respectively.

(3) $$\begin{array}{c} \%YA=\left[\left(\frac{Ha}{Ht}\right)*100\right] \end{array}$$

(4) $$\begin{array}{c} \%RA=\left[\left(\frac{Ha}{A}\right)*100\right] \end{array}$$

Enrichment Calculator (*enrichment.py*) script (https://www.schrodinger.com/scriptcenter) was employed to assess the predictive power of the 3D-QSAR model by a Receiving Operator Curve (ROC). The mentioned script calculates the enrichment metrics, including area under the receiver-operating characteristic curve (AUC), from virtual screening by means of the output structure file and a list of known active molecules. The output of the screening protocol, employing decoys and active molecules, consisted of a list of molecules ranked by the predicted activity from the top-predicted molecules as estimated by the 3D-QSAR model. These ranking data and a list file of active compounds were used as input for *enrichment.py* application.

## Author contributions

GC carried out the computational experiments and performed the acquisition, analysis, and interpretation of data. SG evaluated the integrity of every section of the manuscript and collaborated in writing the introduction. GiCa drafted the work and revised it critically for medicinal chemistry content; and approved the submitted version. SB conceived, designed, and performed the computational experiments, supervised the overall work, wrote and revised the manuscript. StB collected the literature focusing on the selection of compounds used in this study and revised the manuscript. MB analyzed the data, contributing in writing and revising the manuscript.

### Conflict of interest statement

The authors declare that the research was conducted in the absence of any commercial or financial relationships that could be construed as a potential conflict of interest.

## References

[B1] Aronov A. M. (2005). Predictive *in silico* modeling for hERG channel blockers. Drug Discov. Today 10, 149–155. 10.1016/S1359-6446(04)03278-7 15718164

[B2] Aspiotis R. Chen A. Cauchon E. Dubé D. Falgueyret J. P. Gagné S.. (2011). The discovery and synthesis of potent zwitterionic inhibitors of renin. Bioorg. Med. Chem. Lett. 21, 2430–2436. 10.1016/j.bmcl.2011.02.067 21429746

[B3] Barbey J. T. Lazzara R. Zipes D. P. (2002). Spontaneous adverse event reports of serious ventricular arrhythmias, QT prolongation, syncope, and sudden death in patients treated with cisapride. J. Cardiovasc. Pharmacol. Ther. 7, 65–76. 12075394 10.1177/107424840200700202

[B4] Blackburn C. Lamarche M. J. Brown J. Che J. L. Cullis C. A. Lai S.. (2006). Identification and characterization of amino-piperidinequinolones and quinazolinones as MCHr1 antagonists. Bioorg. Med. Chem. Lett. 16, 2621–2627. 10.1016/j.bmcl.2006.02.044 16524729

[B5] Boukharta L. Keränen H. Stary-Weinzinger A. Wallin G. De Groot B. L. Aqvist J. (2011). Computer simulations of structure-activity relationships for HERG channel blockers. Biochemistry 50, 6146–6156. 10.1021/bi200173n 21657256

[B6] Braga R. C. Alves V. M. Silva M. F. B. Muratov E. Fourches D. Lião L. M.. (2015). Pred-hERG: a novel web-accessible computational tool for predicting cardiac toxicity. Mol. Inform. 34, 698–701. 10.1002/minf.201500040 27490970 PMC5720373

[B7] Braga R. C. Andrade C. H. (2013). Assessing the performance of 3D pharmacophore models in virtual screening: how good are they? Curr. Top. Med. Chem. 13, 1127–1138. 10.2174/1568026611313090010 23651486

[B8] Brindisi M. Butini S. Franceschini S. Brogi S. Trotta F. Ros S.. (2014). Targeting dopamine D3 and serotonin 5-HT1A and 5-HT2A receptors for developing effective antipsychotics: synthesis, biological characterization, and behavioral studies. J. Med. Chem. 57, 9578–9597. 10.1021/jm501119j 25343529

[B9] Brogi S. Brindisi M. Joshi B. P. Sanna Coccone S. Parapini S. Basilico N.. (2015). Exploring clotrimazole-based pharmacophore: 3D-QSAR studies and synthesis of novel antiplasmodial agents. Bioorg. Med. Chem. Lett. 25, 5412–5418. 10.1016/j.bmcl.2015.09.007 26428874

[B10] Brogi S. Corelli F. Di Marzo V. Ligresti A. Mugnaini C. Pasquini S.. (2011). Three-dimensional quantitative structure-selectivity relationships analysis guided rational design of a highly selective ligand for the cannabinoid receptor 2. Eur. J. Med. Chem. 46, 547–555. 10.1016/j.ejmech.2010.11.034 21183257

[B11] Brogi S. Giovani S. Brindisi M. Gemma S. Novellino E. Campiani G.. (2016). *In silico* study of subtilisin-like protease 1 (SUB1) from different Plasmodium species in complex with peptidyl-difluorostatones and characterization of potent pan-SUB1 inhibitors. J. Mol. Graph. Model. 64, 121–130. 10.1016/j.jmgm.2016.01.005 26826801 PMC5276822

[B12] Brogi S. Papazafiri P. Roussis V. Tafi A. (2013). 3D-QSAR using pharmacophore-based alignment and virtual screening for discovery of novel MCF-7 cell line inhibitors. Eur. J. Med. Chem. 67, 344–351. 10.1016/j.ejmech.2013.06.048 23880359

[B13] Butini S. Campiani G. Franceschini S. Trotta F. Kumar V. Guarino E.. (2010). Discovery of bishomo (hetero) arylpiperazines as novel multifunctional ligands targeting dopamine D(3) and serotonin 5-HT(1A) and 5-HT(2A) receptors. J. Med. Chem. 53, 4803–4807. 10.1021/jm100294b 20481570

[B14] Butini S. Gemma S. Campiani G. Franceschini S. Trotta F. Borriello M.. (2009). Discovery of a new class of potential multifunctional atypical antipsychotic agents targeting dopamine D3 and serotonin 5-HT1A and 5-HT2A receptors: design, synthesis, and effects on behavior. J. Med. Chem. 52, 151–169. 10.1021/jm800689g 19072656

[B15] Castelli M. P. Casu A. Casti P. Lobina C. Carai M. A. Colombo G.. (2012). Characterization of COR627 and COR628, two novel positive allosteric modulators of the GABA(B) receptor. J. Pharmacol. Exp. Ther. 340, 529–538. 10.1124/jpet.111.186460 22129594

[B16] Coon T. Moree W. J. Li B. Yu J. Zamani-Kord S. Malany S.. (2009). Brain-penetrating 2-aminobenzimidazole H(1)-antihistamines for the treatment of insomnia. Bioorg. Med. Chem. Lett. 19, 4380–4384. 10.1016/j.bmcl.2009.05.086 19553115

[B17] Deacon M. Singleton D. Szalkai N. Pasieczny R. Peacock C. Price D.. (2007). Early evaluation of compound QT prolongation effects: a predictive 384-well fluorescence polarization binding assay for measuring hERG blockade. J. Pharmacol. Toxicol. Methods 55, 238–247. 10.1016/j.vascn.2006.09.003 17141530

[B18] De Bruin M. L. Pettersson M. Meyboom R. H. Hoes A. W. Leufkens H. G. (2005). Anti-HERG activity and the risk of drug-induced arrhythmias and sudden death. Eur. Heart J. 26, 590–597. 10.1093/eurheartj/ehi092 15637086

[B19] Dempsey C. E. Wright D. Colenso C. K. Sessions R. B. Hancox J. C. (2014). Assessing hERG pore models as templates for drug docking using published experimental constraints: the inactivated state in the context of drug block. J. Chem. Inf. Model. 54, 601–612. 10.1021/ci400707h 24471705 PMC3977586

[B20] Dumaine R. Kirsch G. E. (1998). Mechanism of lidocaine block of late current in long Q-T mutant Na+ channels. Am. J. Physiol. 274, H477–H487. 9486250 10.1152/ajpheart.1998.274.2.H477

[B21] Dumaine R. Roy M. L. Brown A. M. (1998). Blockade of HERG and Kv1.5 by ketoconazole. J. Pharmacol. Exp. Ther. 286, 727–735. Available online at: http://jpet.aspetjournals.org/content/286/2/727/tab-article-info 9694927

[B22] Durdagi S. Deshpande S. Duff H. J. Noskov S. Y. (2012). Modeling of open, closed, and open-inactivated states of the hERG1 channel: structural mechanisms of the state-dependent drug binding. J. Chem. Inf. Model. 52, 2760–2774. 10.1021/ci300353u 22989185

[B23] Durdagi S. Duff H. J. Noskov S. Y. (2011). Combined receptor and ligand-based approach to the universal pharmacophore model development for studies of drug blockade to the hERG1 pore domain. J. Chem. Inf. Model. 51, 463–474. 10.1021/ci100409y 21241063

[B24] Farid R. Day T. Friesner R. A. Pearlstein R. A. (2006). New insights about HERG blockade obtained from protein modeling, potential energy mapping, and docking studies. Bioorg. Med. Chem. 14, 3160–3173. 10.1016/j.bmc.2005.12.032 16413785

[B25] Finlayson K. Turnbull L. January C. T. Sharkey J. Kelly J. S. (2001). [^3^H]dofetilide binding to HERG transfected membranes: a potential high throughput preclinical screen. Eur. J. Pharmacol. 430, 147–148. 10.1016/S0014-2999(01)01362-0 11698075

[B26] Fletcher S. R. Burkamp F. Blurton P. Cheng S. K. Clarkson R. O'connor D.. (2002). 4-(Phenylsulfonyl)piperidines: novel, selective, and bioavailable 5-HT(2A) receptor antagonists. J. Med. Chem. 45, 492–503. 10.1021/jm011030v 11784153

[B27] Gemma S. Camodeca C. Brindisi M. Brogi S. Kukreja G. Kunjir S.. (2012). Mimicking the intramolecular hydrogen bond: synthesis, biological evaluation, and molecular modeling of benzoxazines and quinazolines as potential antimalarial agents. J. Med. Chem. 55, 10387–10404. 10.1021/jm300831b 23145816

[B28] Gottlieb S. (1999). Antihistamine drug withdrawn by manufacturer. BMJ 319, 7. 10390435 10.1136/bmj.319.7201.7a PMC1116178

[B29] Honig P. K. Wortham D. C. Zamani K. Conner D. P. Mullin J. C. Cantilena L. R. (1993). Terfenadine-ketoconazole interaction. Pharmacokinetic and electrocardiographic consequences. JAMA 269, 1513–1518. 10.1001/jama.1993.03500120051025 8445813

[B30] Huang N. Shoichet B. K. Irwin J. J. (2006). Benchmarking sets for molecular docking. J. Med. Chem. 49, 6789–6801. 10.1021/jm0608356 17154509 PMC3383317

[B31] Jorgensen W. L. Maxwell D. S. Tiradorives J. (1996). Development and testing of the OPLS all atom force field on conformational energetics and properties of organic liquids. J. Am. Chem. Soc. 118, 11225–11236. 10.1021/ja9621760

[B32] Keserü G. M. (2003). Prediction of hERG potassium channel affinity by traditional and hologram QSAR methods. Bioorg. Med. Chem. Lett. 13, 2773–2775. 10.1016/S0960-894x(03)00492-X 12873512

[B33] Krishna S. Singh D. K. Meena S. Datta D. Siddiqi M. I. Banerjee D. (2014). Pharmacophore-based screening and identification of novel human ligase I inhibitors with potential anticancer activity. J. Chem. Inf. Model. 54, 781–792. 10.1021/ci5000032 24593844

[B34] Levoin N. Labeeuw O. Calmels T. Poupardin-Olivier O. Berrebi-Bertrand I. Lecomte J. M.. (2011). Novel and highly potent histamine H3 receptor ligands. Part 1: withdrawing of hERG activity. Bioorg. Med. Chem. Lett. 21, 5378–5383. 10.1016/j.bmcl.2011.07.006 21802950

[B35] Liu H. Altenbach R. J. Diaz G. J. Manelli A. M. Martin R. L. Miller T. R.. (2010). *In vitro* studies on a class of quinoline containing histamine H3 antagonists. Bioorg. Med. Chem. Lett. 20, 3295–3300. 10.1016/j.bmcl.2010.04.045 20457525

[B36] Masetti M. Cavalli A. Recanatini M. (2008). Modeling the hERG potassium channel in a phospholipid bilayer: molecular dynamics and drug docking studies. J. Comput. Chem. 29, 795–808. 10.1002/jcc.20842 17926340

[B37] Mohammad S. Zhou Z. Gong Q. January C. T. (1997). Blockage of the HERG human cardiac K+ channel by the gastrointestinal prokinetic agent cisapride. Am. J. Physiol. 273, H2534–H2538. Available online at: http://ajpheart.physiology.org/content/273/5/H2534.full.pdf+html 9374794 10.1152/ajpheart.1997.273.5.H2534

[B38] Murphy S. M. Palmer M. Poole M. F. Padegimas L. Hunady K. Danzig J.. (2006). Evaluation of functional and binding assays in cells expressing either recombinant or endogenous hERG channel. J. Pharmacol. Toxicol. Methods 54, 42–55. 10.1016/j.vascn.2005.10.003 16326118

[B39] Mysinger M. M. Carchia M. Irwin J. J. Shoichet B. K. (2012). Directory of useful decoys, enhanced (DUD-E): better ligands and decoys for better benchmarking. J. Med. Chem. 55, 6582–6594. 10.1021/jm300687e 22716043 PMC3405771

[B40] Owen D. R. Rodriguez-Lens M. Corless M. D. Gaulier S. M. Horne V. A. Kinloch R. A.. (2009). 2,4-Diaminopyridine delta-opioid receptor agonists and their associated hERG pharmacology. Bioorg. Med. Chem. Lett. 19, 1702–1706. 10.1016/j.bmcl.2009.01.106 19231185

[B41] Pasquini S. Mugnaini C. Ligresti A. Tafi A. Brogi S. Falciani C.. (2012). Design, synthesis, and pharmacological characterization of indole-3-yl-acetamides, -oxoacetamides, and -carboxamides: potent and selective CB2 cannabinoid receptor inverse agonists. J. Med. Chem. 55, 5391–5402. 10.1021/jm3003334 22548457

[B42] Patel S. D. Habeski W. M. Cheng A. C. de La Cruz E. Loh C. Kablaoui N. M. (2009). Quinazolin-4-piperidin-4-methyl sulfamide PC-1 inhibitors: alleviating hERG interactions through structure based design. Bioorg. Med. Chem. Lett. 19, 3339–3343. 10.1016/j.bmcl.2009.04.006 19435660

[B43] Rajamani S. Eckhardt L. L. Valdivia C. R. Klemens C. A. Gillman B. M. Anderson C. L.. (2006). Drug-induced long QT syndrome: hERG K+ channel block and disruption of protein trafficking by fluoxetine and norfluoxetine. Br. J. Pharmacol. 149, 481–489. 10.1038/sj.bjp.0706892 16967046 PMC2014667

[B44] Ray W. A. Murray K. T. Meredith S. Narasimhulu S. S. Hall K. Stein C. M. (2004). Oral erythromycin and the risk of sudden death from cardiac causes. N. Engl. J. Med. 351, 1089–1096. 10.1056/NEJMoa040582 15356306

[B45] Redfern W. S. Carlsson L. Davis A. S. Lynch W. G. Mackenzie I. Palethorpe S.. (2003). Relationships between preclinical cardiac electrophysiology, clinical QT interval prolongation and torsade de pointes for a broad range of drugs: evidence for a provisional safety margin in drug development. Cardiovasc. Res. 58, 32–45. 10.1016/S0008-6363(02)00846-5 12667944

[B46] Roden D. M. (2004). Drug-induced prolongation of the QT interval. N.Engl. J. Med. 350, 1013–1022. 10.1056/NEJMra032426 14999113

[B47] Sakkiah S. Thangapandian S. John S. Lee K. W. (2011). Pharmacophore based virtual screening, molecular docking studies to design potent heat shock protein 90 inhibitors. Eur. J. Med. Chem. 46, 2937–2947. 10.1016/j.ejmech.2011.04.018 21531051

[B48] Sanguinetti M. C. Jiang C. Curran M. E. Keating M. T. (1995). A mechanistic link between an inherited and an acquired cardiac arrhythmia: *HERG* encodes the I_Kr_ potassium channel. Cell 81, 299–307. 10.1016/0092-8674(95)90340-2 7736582

[B49] Sanguinetti M. C. Tristani-Firouzi M. (2006). hERG potassium channels and cardiac arrhythmia. Nature 440, 463–469. 10.1038/nature04710 16554806

[B50] Still W. C. Tempczyk A. Hawley R. C. Hendrickson T. (1990). Semianalytical treatment of solvation for molecular mechanics and dynamics. J. Am. Chem. Soc. 112, 6127–6129. 10.1021/Ja00172a038

[B51] Thangapandian S. John S. Sakkiah S. Lee K. W. (2011). Pharmacophore-based virtual screening and Bayesian model for the identification of potential human leukotriene A4 hydrolase inhibitors. Eur. J. Med. Chem. 46, 1593–1603. 10.1016/j.ejmech.2011.02.007 21377770

[B52] Thomas D. Kathofer S. Zhang W. Wu K. Wimmer A. B. Zitron E.. (2003a). Acute effects of dronedarone on both components of the cardiac delayed rectifier K+ current, HERG and KvLQT1/minK potassium channels. Br. J. Pharmacol. 140, 996–1002. 10.1038/sj.bjp.0705502 14517175 PMC1574095

[B53] Thomas D. Wu K. Kathöfer S. Katus H. A. Schoels W. Kiehn J.. (2003b). The antipsychotic drug chlorpromazine inhibits HERG potassium channels. Br. J. Pharmacol. 139, 567–574. 10.1038/sj.bjp.0705283 12788816 PMC1573882

[B54] Triballeau N. Acher F. Brabet I. Pin J. P. Bertrand H. O. (2005). Virtual screening workflow development guided by the “receiver operating characteristic” curve approach. Application to high-throughput docking on metabotropic glutamate receptor subtype 4. J. Med. Chem. 48, 2534–2547. 10.1021/jm049092j 15801843

[B55] Trudeau M. C. Warmke J. W. Ganetzky B. Robertson G. A. (1995). HERG, a human inward rectifier in the voltage-gated potassium channel family. Science 269, 92–95. 10.1126/science.7604285 7604285

[B56] Wang S. Li Y. Xu L. Li D. Hou T. (2013). Recent developments in computational prediction of HERG blockage. Curr. Top. Med. Chem. 13, 1317–1326. 10.2174/15680266113139990036 23675938

[B57] Wang S. Sun H. Liu H. Li D. Li Y. Hou T. (2016). ADMET evaluation in drug discovery. 16. Predicting hERG Blockers by combining multiple pharmacophores and machine learning approaches. Mol. Pharm. 13, 2855–2866. 10.1021/acs.molpharmaceut.6b00471 27379394

[B58] Zaccagnini L. Brogi S. Brindisi M. Gemma S. Chemi G. Legname G.. (2017). Identification of novel fluorescent probes preventing PrPSc replication in prion diseases. Eur. J. Med. Chem. 127, 859–873. 10.1016/j.ejmech.2016.10.064 27842893

[B59] Zhao W. Hevener K. E. White S. W. Lee R. E. Boyett J. M. (2009). A statistical framework to evaluate virtual screening. BMC Bioinformatics 10:225. 10.1186/1471-2105-10-225 19619306 PMC2722655

[B60] Zhu B. Y. Jia Z. J. Zhang P. Su T. Huang W. Goldman E.. (2006). Inhibitory effect of carboxylic acid group on hERG binding. Bioorg. Med. Chem. Lett. 16, 5507–5512. 10.1016/j.bmcl.2006.08.039 16931010

