# Supplementary material for: Computational Tool for Fast in silico Evaluation of hERG K+ Channel Affinity
Source: Front Chem. 2017 Feb 23;5:7. doi: 10.3389/fchem.2017.00007 (PMC5408157; doi:10.3389/fchem.2017.00007)
Supplement: Supplementary file 1 [file Table1.DOCX]

**Table S1** Experimental (Observed column) and predicted (Predicted column) activity p*Ki* (M) for compounds for developing the 3D-QSAR model. (Note: a) compounds included in the training set; b) compounds included in the tests set; * compounds used for the decoys generation).

| **Compound** | | | **Observed *Ki* (nM)** | **Observed p*Ki*** | **Predicted p*Ki*** | **Note** |
| --- | --- | --- | --- | --- | --- | --- |
|  | c1cc(OC)ccc1CCN(CC2)CCC2Nc(n3)n(Cc(cc4)ccc4F)c(c35)cccc5  **Astemizole** | ([Murphy et al., 2006](#_ENREF_27)) | 3.3 | 8.48 | 8.59 | a,* |
|  | Fc1ccc(cc1)Cn(c(c23)cccc2)c(n3)N(CC4)CCC4N(C)CC5CCOCC5 | ([Coon et al., 2009](#_ENREF_8)) | 18 | 7.75 | 7.44 | a,* |
|  | Fc1ccc(cc1)Cn(c(c23)cccc2)c(n3)N(CC4)CCC4N(C)CCOC | ([Coon et al., 2009](#_ENREF_8)) | 40 | 7.40 | 6.70 | b,* |
|  | O=S(=O)(C)Nc1ccc(cc1)OCCN(C)CCc(cc2)ccc2NS(=O)(=O)C  **Dofetilide** | ([Deacon et al., 2007](#_ENREF_9)) | 6.3 | 8.20 | 8.44 | a,* |
|  | C1CCCCC1c2ccc(cc2)OCCCN3CCCCC3 | ([Levoin et al., 2011](#_ENREF_20)) | 13 | 7.89 | 7.15 | a,* |
|  | s1cccc1-c2ccc(cc2)OCCCN3CCCCC3 | ([Levoin et al., 2011](#_ENREF_20)) | 14 | 7.85 | 7.21 | a,* |
|  | CCOC(=O)c1ccc(cc1)-c2ccc(cc2)OCCCN3CCCCC3 | ([Levoin et al., 2011](#_ENREF_20)) | 8 | 8.10 | 7.98 | a,* |
|  | O1COc(c12)cc(cc2)-c3ccc(cc3)OCCCN4CCCCC4 | ([Levoin et al., 2011](#_ENREF_20)) | 5 | 8.30 | 7.90 | a,* |
|  | Nc1ccnc(c1)N(CC2)CCC2(c3ccccc3)C(=O)N(CC4)Cc(c45)cccc5 | ([Owen et al., 2009](#_ENREF_28)) | 35 | 7.46 | 7.59 | a,* |
|  | Nc1ccnc(c1)N(CC2)CCC2(c3ccccc3)C(=O)Nc(n4)ccc(c45)cccc5 | ([Owen et al., 2009](#_ENREF_28)) | 17 | 7.77 | 7.96 | a,* |
|  | Clc1cnc(cc1)NC(=O)c(cc2Cl)c(cc2)NC(=O)c3ccc(cc3)/C(=N/[H])N4CCC(CC4)C(=O)OCC | ([Zhu et al., 2006](#_ENREF_35)) | 17 | 7.77 | 7.63 | a,* |
|  | CCOC(=O)[C@@H](CCC1)CN1C(=N/[H])\c(cc2)ccc2C(=O)Nc(cc3)c(cc3Cl)C(=O)Nc(cc4)ncc4Cl | ([Zhu et al., 2006](#_ENREF_35)) | 16 | 7.79 | 7.88 | a,* |
|  | Clc1cnc(cc1)NC(=O)c(cc2Cl)c(c(c2)OC)NC(=O)c3ccc(cc3F)/C(=N/[H])N4CCC(CC4)C(=O)OCC | ([Zhu et al., 2006](#_ENREF_35)) | 51 | 7.29 | 6.63 | b,* |
|  | Clc1ccc(cc1)CCCC[N+](CC)(CC)CCCCCCC  **Clofilium** | ([Finlayson et al., 2001](#_ENREF_13)) | 8.12 | 8.09 | 8.30 | a,* |
|  | C1CC[C@@H](C)N1CCc(cc2)cc(c23)ccc(n3)-c(c4)nc(nc4C)-c5ccccc5 | ([Liu et al., 2010](#_ENREF_23)) | 30 | 7.52 | 6.91 | a,* |
|  | c1c(N)ccnc1N(CC2)CCC23c4c(cccc4)[C@@H](O3)c5ccccc5 | ([Owen et al., 2009](#_ENREF_28)) | 18 | 7.75 | 8.03 | a,* |
|  | c1cc(OC)cc(CC2)c1OC23CCN(CC3)CCc(c(F)c4)ccc4F | ([Fletcher et al., 2002](#_ENREF_16)) | 20 | 7.69 | 7.83 | a,* |
|  | c1cc(F)ccc1C(c(cc2)ccc2F)CCCN(CC3)CCC3n4c(=O)[nH]c(c45)cccc5  **Pimozide** | ([Murphy et al., 2006](#_ENREF_27)) | 19 | 7.72 | 6.90 | b,* |
|  | c1cc(Cl)cc(c12)c(cc(=O)n2C)NC3CCN(CC3)C/C=C/c4ccccc4 | ([Blackburn et al., 2006](#_ENREF_3)) | 30 | 7.52 | 7.61 | a,* |
|  | O=S(=O)(N)NCCC(CC1)CCN1c(nc(n2)CC)c(c23)cc(OC)c(c3)OC | ([Patel et al., 2009](#_ENREF_30)) | 17 | 7.77 | 8.16 | a,* |
|  | Cc1cc(Cl)c(c(Cl)c1)OCCOc2ccc(cc2)[C@H]3CCNC[C@@H]3C(=O)N(C4CC4)Cc(c5)cc(CCCOC)cc5OC[C@@H]6C[C@@H]6C(=O)OCOC(=O)C(C)(C)C | ([Aspiotis et al., 2011](#_ENREF_2)) | 40 | 7.40 | 7.28 | a,* |
|  | CS(=O)(=O)Nc1ccc(cc1)C(=O)C2CCN(CC2)CCc(n3)cccc3C  **E4031** | ([Deacon et al., 2007](#_ENREF_9)) | 29 | 7.54 | 7.54 | a,* |
|  | CN(C)C(=N/[H])\c(cc1)ccc1C(=O)Nc(cc2)c(C(=O)Nc(cc3)ncc3Cl)cc2Sc4ccccc4 | ([Zhu et al., 2006](#_ENREF_35)) | 89 | 7.05 | 6.96 | a,* |
|  | CN(C)C(=N/[H])\c(cc1)ccc1C(=O)Nc(cc2)c(C(=O)Nc(cc3)ncc3Cl)cc2Sc(cc4)ccc4C(=O)OC | ([Zhu et al., 2006](#_ENREF_35)) | 71 | 7.15 | 6.94 | a,* |
|  | CN(C)C(=N/[H])\c(cc1)ccc1C(=O)Nc(cc2)c(C(=O)Nc(cc3)ncc3Cl)cc2Sc(cc4)ccc4C(=O)N(C)C | ([Zhu et al., 2006](#_ENREF_35)) | 370 | 6.43 | 6.57 | b |
|  | CN(C)C(=N/[H])\c(cc1)ccc1C(=O)Nc(cc2)c(C(=O)Nc(cc3)ncc3Cl)cc2Sc(cc4)ccc4C(=O)O | ([Zhu et al., 2006](#_ENREF_35)) | 4,600 | 5.34 | 5.96 | a |
|  | CN(C)C(=N/[H])\c(cc1)ccc1C(=O)Nc(cc2)c(C(=O)Nc(cc3)ncc3Cl)cc2Oc(cc4)ccc4C(=O)OC | ([Zhu et al., 2006](#_ENREF_35)) | 570 | 6.24 | 6.14 | b |
|  | CN(C)C(=N/[H])\c(cc1)ccc1C(=O)Nc(cc2)c(C(=O)Nc(cc3)ncc3Cl)cc2CCC(=O)OC | ([Zhu et al., 2006](#_ENREF_35)) | 790 | 6.10 | 6.29 | a |
|  | CN(C)C(=N/[H])\c(cc1)ccc1C(=O)Nc(cc2)c(C(=O)Nc(cc3)ncc3Cl)cc2OCC(=O)OCC | ([Zhu et al., 2006](#_ENREF_35)) | 970 | 6.01 | 5.60 | a |
|  | [H]\N=C(N(C)C)\c(cc1)ccc1C(=O)Nc(cc2)c(cc2Cl)C(=O)Nc(cc3)ncc3Cl | ([Zhu et al., 2006](#_ENREF_35)) | 100 | 7.00 | 6.42 | b,* |
|  | OC(=O)CN(C)C(=N/[H])\c(cc1)ccc1C(=O)Nc(cc2)c(cc2Cl)C(=O)Nc(cc3)ncc3Cl | ([Zhu et al., 2006](#_ENREF_35)) | 1,400 | 5.85 | 6.18 | a |
|  | O=C(O)CCCN(C)C(=N/[H])\c(cc1)ccc1C(=O)Nc(cc2)c(cc2Cl)C(=O)Nc(cc3)ncc3Cl | ([Zhu et al., 2006](#_ENREF_35)) | 1,400 | 5.85 | 5.86 | a |
|  | C1CCCCN1c2cc(/C(=N/[H])N(C)C)ccc2C(=O)Nc(cc3)c(cc3Cl)C(=O)Nc(cc4)ncc4Cl | ([Zhu et al., 2006](#_ENREF_35)) | 140 | 6.85 | 6.67 | a,* |
|  | C1CC[C@@H](C(=O)O)CN1c2cc(/C(=N/[H])N(C)C)ccc2C(=O)Nc(cc3)c(cc3Cl)C(=O)Nc(cc4)ncc4Cl | ([Zhu et al., 2006](#_ENREF_35)) | 1,600 | 5.80 | 5.82 | a |
|  | C1CCCCN1c2cc(/C(=N/[H])N(C)C)ccc2C(=O)Nc(cc3)c(cc3OC)C(=O)Nc(cc4)ncc4Cl | ([Zhu et al., 2006](#_ENREF_35)) | 200 | 6.70 | 6.03 | b |
|  | Clc1cnc(cc1)NC(=O)c(cc2OC)c(cc2)NC(=O)c3ccc(/C(=N/[H])N(C)C)cc3N4CCC(CC4)C(=O)OCC | ([Zhu et al., 2006](#_ENREF_35)) | 600 | 6.22 | 6.12 | b |
|  | CN1CCN=C1c(cc2)ccc2C(=O)N(CC3)[C@@H](CC(=O)N4CCOCC4)CN3S(=O)(=O)c(c5)sc(c56)cc(Cl)cc6 | ([Zhu et al., 2006](#_ENREF_35)) | 600 | 6.22 | 6.15 | a |
|  | CN(C)C(=N/[H])\c(cc1)ccc1CN(C(=O)C2)[C@@H](C(=O)OCC)CN2S(=O)(=O)c(c3)[nH]c(c34)ccc(Cl)c4 | ([Zhu et al., 2006](#_ENREF_35)) | 71 | 7.15 | 7.20 | a,* |
|  | C1CCCN1C(=N/[H])\c(cc2)ccc2NC(=O)c3cc(C)nn3-c(c4)c(C#N)cc(c45)cccc5 | ([Zhu et al., 2006](#_ENREF_35)) | 880 | 6.06 | 5.96 | b |
|  | c1cccc(S(=O)(=O)N)c1-c(cc2)ccc2NC(=O)/C(F)=C(/C)c3cccc(c3)/C(=N/[H])N | ([Zhu et al., 2006](#_ENREF_35)) | 530 | 6.28 | 6.24 | a |
|  | [H]/N=C(N)\c(cc1)cc(c12)cc(cc2)CN(S(=O)(=O)C)c3ccc(cc3)OC4CCN(CC4)/C(=N/[H])C | ([Zhu et al., 2006](#_ENREF_35)) | 3,900 | 5.41 | 6.07 | b |
|  | C1CCCCN1Cc(cccc2)c2-c(cc3)cc(F)c3NC(=O)c4cc(C)nn4-c(c5)c(F)cc(c56)cccc6 | ([Zhu et al., 2006](#_ENREF_35)) | 1,000 | 6.00 | 6.03 | b |
|  | c1cccc(c12)oc(CCCC)c2C(=O)c(cc3I)cc(I)c3OCCN(CC)CC **Amiodarone** | ([Diaz et al., 2004](#_ENREF_10)) | 400 | 6.40 | 6.43 | b |
|  | N1C(=O)CCc(c12)ccc(c2)OCCCCN(CC3)CCN3c(c4Cl)cccc4Cl **Aripiprazole** | ([Diaz et al., 2004](#_ENREF_10)) | 2,450 | 5.61 | 5.65 | a |
|  | c1ccccc1CN(c2ccccc2)C[C@H](COCC(C)C)N3CCCC3  **Bepridil** | ([Diaz et al., 2004](#_ENREF_10)) | 170 | 6.77 | 6.36 | b |
|  | c1cccc(c12)Sc3c(cc(Cl)cc3)N2CCCN(C)C  **Chlorpromazine** | ([Diaz et al., 2004](#_ENREF_10)) | 780 | 6.11 | 6.09 | a |
|  | c1c(Cl)ccc(c12)Nc3c(cccc3)C(=N2)N(CC4)CCN4C  **Clozapine** | ([Diaz et al., 2004](#_ENREF_10)) | 1,200 | 5.92 | 5.69 | a |
|  | c1c(Cl)ccc(c12)Nc3c(cccc3)C(=N2)N4CCNCC4  **N-des-methyl-clozapine** | ([Diaz et al., 2004](#_ENREF_10)) | 4,040 | 5.39 | 5.57 | b |
|  | c1cccc(c12)S[C@@H](c(cc3)ccc3OC)[C@@H](OC(=O)C)C(=O)N2CCN(C)C  **Diltiazem** | ([Diaz et al., 2004](#_ENREF_10)) | 31,200 | 4.51 | 4.58 | a |
|  | c1ccccc1C(OCCN(C)C)c2ccccc2  **Diphenhydramine** | ([Diaz et al., 2004](#_ENREF_10)) | 4,140 | 5.38 | 5.55 | a |
|  | c1cccc(c12)[nH]c(=O)n2CCCN3CCC(CC3)n(c(=O)[nH]4)c(c45)ccc(Cl)c5  **Domperidone** | ([Diaz et al., 2004](#_ENREF_10)) | 220 | 6.66 | 6.43 | b |
|  | Fc1ccc(cc1)C(=O)CCCN2CC=C(CC2)n(c(=O)[nH]3)c(c34)cccc4  **Droperidol** | ([Diaz et al., 2004](#_ENREF_10)) | 120 | 6.92 | 7.10 | a,* |
|  | c1ccccc1C(c2ccccc2)OC(CC3)CCN3CCCC(=O)c(cc4)ccc4C(C)(C)C  **Ebastine** | ([Diaz et al., 2004](#_ENREF_10)) | 100 | 7.00 | 7.19 | a,* |
|  | c1ccccc1C(O)(c2ccccc2)C3CCN(CC3)CCC[C@@H](O)c(cc4)ccc4C(C)(C)C(=O)O  **Fexofenadine** | ([Diaz et al., 2004](#_ENREF_10)) | 32,000 | 4.49 | 4.51 | a |
|  | FC(F)(F)COc(cc1)cc(c1OCC(F)(F)F)C(=O)NC[C@H]2NCCCC2  **Flecainide** | ([Diaz et al., 2004](#_ENREF_10)) | 4,540 | 5.34 | 5.39 | b |
|  | c1cc(F)ccc1C(c(cc2)ccc2F)OCCN3CCN(CC3)CCCc4ccccc4  **Vanoxerine_GBR-12909** | ([Diaz et al., 2004](#_ENREF_10)) | 60 | 7.22 | 7.39 | a,* |
|  | C1CN(C(=O)C)CCN1c(cc2)ccc2OC[C@H](O3)CO[C@@]3(c(cc4)c(Cl)cc4Cl)Cn5ccnc5  **Ketoconazole** | ([Diaz et al., 2004](#_ENREF_10)) | 19,500 | 4.71 | 4.52 | a |
|  | c1cccc(c12)Sc3c(cc(cc3)[S@](=O)C)N2CC[C@@H]4CCCCN4C  **Mesoridazine** | ([Diaz et al., 2004](#_ENREF_10)) | 1,790 | 5.75 | 5.77 | a |
|  | c1cc(F)cc(c12)CC[C@]([C@H]2C(C)C)(OC(=O)COC)CCN(C)CCCc(n3)[nH]c(c34)cccc4  **Mibefradil** | ([Diaz et al., 2004](#_ENREF_10)) | 660 | 6.18 | 6.24 | a |
|  | c1cc(OC)ccc1CN(CCN(C)C)c2ccccn2  **Pyrilamine** | ([Diaz et al., 2004](#_ENREF_10)) | 5,000 | 5.30 | 5.86 | b |
|  | c1cccc(c12)Sc3c(cccc3)C(=N2)N(CC4)CCN4CCOCCO  **Quetiapine** | ([Diaz et al., 2004](#_ENREF_10)) | 8,040 | 5.09 | 5.01 | a |
|  | C[C@H]1N[C@@H](C)CN(C1)c(c(F)c2N)c(F)c(c23)n(C4CC4)cc(c3=O)C(=O)O  **Sparfloxacin** | ([Diaz et al., 2004](#_ENREF_10)) | 18,800 | 4.73 | 4.59 | a |
|  | c1cc(C)nc(c12)cccc2N(CC3)CCN3CCc(c4)ccc(c45)OCC(=O)N5 | ([Bromidge et al., 2009](#_ENREF_6)) | 63 | 7.20 | 6.94 | a,* |
|  | c1cc(C)nc(c12)cccc2N(CC3)CCN3CCc(c4)ccc(c45)OCC(=O)N5C | ([Bromidge et al., 2009](#_ENREF_6)) | 794 | 6.10 | 6.56 | a |
|  | c1cc(C)nc(c12)cccc2N(CC3)CCN3CCc(ccc4)c(c45)OCC(=O)N5 | ([Bromidge et al., 2009](#_ENREF_6)) | 2,512 | 5.60 | 5.43 | b |
|  | c1cc(C)nc(c12)cccc2N(CC3)CCN3CCc(ccc4)c(c45)OCC(=O)N5C | ([Bromidge et al., 2009](#_ENREF_6)) | 3,981 | 5.40 | 5.53 | b |
|  | c1cc(C)nc(c12)cccc2N(CC3)CCN3CCc(cc4)cc(c45)OCC(=O)N5 | ([Bromidge et al., 2009](#_ENREF_6)) | 100 | 7.00 | 6.93 | a,* |
|  | c1cc(C)nc(c12)cccc2N(CC3)CCN3CCc(cc4)cc(c45)OCC(=O)N5C | ([Bromidge et al., 2009](#_ENREF_6)) | 3,162 | 5.50 | 5.63 | a |
|  | c1cc(C)nc(c12)cccc2N(CC3)CCN3CCc(ccc4)c(c45)OCC(=O)N5CC | ([Bromidge et al., 2009](#_ENREF_6)) | 7,943 | 5.10 | 5.31 | a |
|  | c1cc(C)nc(c12)cccc2N(CC3)CCN3CCc(ccc4)c(c45)OCC(=O)N5C(C)C | ([Bromidge et al., 2009](#_ENREF_6)) | 5,012 | 5.30 | 5.39 | a |
|  | c1cc(C)nc(c12)cccc2N(CC3)CCN3CCc(ccc4)c(c45)OCC(=O)N5C6CC6 | ([Bromidge et al., 2009](#_ENREF_6)) | 1,585 | 5.80 | 5.57 | a |
|  | c1cc(C)nc(c12)cccc2N(CC3)CCN3CCc(ccc4)c(c45)OCC(=O)N5CC6CC6 | ([Bromidge et al., 2009](#_ENREF_6)) | 1,585 | 5.80 | 5.50 | a |
|  | c1cc(C)nc(c12)cccc2N(CC3)CCN3CCc(ccc4)c(c45)OCC(=O)N5C(F)F | ([Bromidge et al., 2009](#_ENREF_6)) | 398 | 6.40 | 5.65 | a |
|  | c1cc(C)nc(c12)cccc2N(CC3)C[C@@H](C)N3CCc(ccc4)c(c45)OCC(=O)N5C | ([Bromidge et al., 2009](#_ENREF_6)) | 10,000 | 5.00 | 5.29 | a |
|  | c1cc(C)nc(c12)cccc2N(CC3)C[C@H](C)N3CCc(ccc4)c(c45)OCC(=O)N5C | ([Bromidge et al., 2009](#_ENREF_6)) | 12,589 | 4.90 | 5.14 | a |
|  | c1cc(C)nc(c12)cc(F)cc2N(CC3)CCN3CCc(ccc4)c(c45)OCC(=O)N5C | ([Bromidge et al., 2009](#_ENREF_6)) | 3,981 | 5.40 | 5.48 | a |
|  | c1cc(C)nc(c12)cc(F)cc2N(CC3)C[C@@H](C)N3CCc(ccc4)c(c45)OCC(=O)N5C | ([Bromidge et al., 2009](#_ENREF_6)) | 3,162 | 5.50 | 5.88 | b |
|  | c1cc(C)nc(c12)cccc2N(CC3)CCN3CCCc(ccc4)c(c45)OCC(=O)N5C | ([Bromidge et al., 2009](#_ENREF_6)) | 5,012 | 5.30 | 5.81 | b |
|  | c1cc(C)nc(c12)cccc2N(CC3)C[C@@H](C)N3CCCc(ccc4)c(c45)OCC(=O)N5C | ([Bromidge et al., 2009](#_ENREF_6)) | 6,310 | 5.20 | 5.25 | a |
|  | c1cc(C)nc(c12)cc(F)cc2N(CC3)CCN3CCCc(ccc4)c(c45)OCC(=O)N5C | ([Bromidge et al., 2009](#_ENREF_6)) | 6,310 | 5.20 | 5.58 | b |
|  | c1cc(C)nc(c12)cc(F)cc2N(CC3)C[C@@H](C)N3CCCc(ccc4)c(c45)OCC(=O)N5C | ([Bromidge et al., 2009](#_ENREF_6)) | 3,162 | 5.50 | 5.51 | a |
|  | c1cc(C)nc(c12)cccc2N(CC3)CCN3CCCc(ccc4)c(c45)OCC(=O)N5 | ([Bromidge et al., 2009](#_ENREF_6)) | 631 | 6.20 | 5.79 | b |
|  | c1cc(C)nc(c12)cccc2N(CC3)CCN3CCc(ccc4)c(c45)CCC(=O)N5 | ([Bromidge et al., 2010](#_ENREF_5)) | 3,162 | 5.50 | 5.49 | b |
|  | c1cc(C)nc(c12)cccc2N(CC3)CCN3CCc(ccc4)c(c45)CCC(=O)N5C | ([Bromidge et al., 2010](#_ENREF_5)) | 3,981 | 5.40 | 5.53 | a |
|  | c1cc(C)nc(c12)cccc2N(CC3)CCN3CCc(c(C)cc4)c(c45)CCC(=O)N5 | ([Bromidge et al., 2010](#_ENREF_5)) | 3,981 | 5.40 | 5.38 | a |
|  | c1cc(C)nc(c12)cccc2N(CC3)CCN3CCc(c(C)cc4)c(c45)CCC(=O)N5C | ([Bromidge et al., 2010](#_ENREF_5)) | 794 | 6.10 | 5.67 | a |
|  | c1cc(C)nc(c12)cccc2N(CC3)CCN3CCc(ccc4)c(c45)ccc(=O)n5C | ([Bromidge et al., 2010](#_ENREF_5)) | 10,000 | 5.00 | 4.96 | b |
|  | c1cc(C)nc(c12)cccc2N(CC3)CCN3CCc(c(C)cc4)c(c45)ccc(=O)[nH]5 | ([Bromidge et al., 2010](#_ENREF_5)) | 25,119 | 4.60 | 4.81 | a |
|  | c1cc(C)nc(c12)cccc2N(CC3)CCN3CCc(c(C)cc4)c(c45)ccc(=O)n5C | ([Bromidge et al., 2010](#_ENREF_5)) | 3,981 | 5.40 | 5.05 | b |
|  | c1cc(C)nc(c12)cccc2N(CC3)CCN3CCc(c(F)cc4)c(c45)ccc(=O)n5C | ([Bromidge et al., 2010](#_ENREF_5)) | 31,623 | 4.50 | 4.97 | b |
|  | c1cc(C)nc(c12)cccc2N(CC3)CCN3CCc(c(Cl)cc4)c(c45)ccc(=O)n5C | ([Bromidge et al., 2010](#_ENREF_5)) | 5,012 | 5.30 | 5.08 | a |
|  | c1cc(C)nc(c12)cc(F)cc2N(CC3)CCN3CCc(c(C)cc4)c(c45)ccc(=O)n5C | ([Bromidge et al., 2010](#_ENREF_5)) | 2,512 | 5.60 | 5.28 | a |
|  | c1cc(C)nc(c12)cccc2N(C[C@H]3C)CCN3CCc(c(C)cc4)c(c45)ccc(=O)n5C | ([Bromidge et al., 2010](#_ENREF_5)) | 7,943 | 5.10 | 5.09 | a |
|  | c1cc(C)nc(c12)cc(F)cc2N(C[C@H]3C)CCN3CCc(ccc4)c(c45)ccc(=O)n5C | ([Bromidge et al., 2010](#_ENREF_5)) | 10,000 | 5.00 | 5.10 | b |
|  | c1cc(C)nc(c12)cccc2N(C[C@@H]3C)CCN3CCc(ccc4)c(c45)ccc(=O)n5C | ([Bromidge et al., 2010](#_ENREF_5)) | 5,012 | 5.30 | 5.53 | b |
|  | c1cc(C)nc(c12)cccc2N(C[C@@H]3C)CCN3CCc(c(C)cc4)c(c45)ccc(=O)n5C | ([Bromidge et al., 2010](#_ENREF_5)) | 1,584 | 5.80 | 5.60 | a |
|  | c1cc(C)nc(c12)cc(F)cc2N(C[C@@H]3C)CCN3CCc(ccc4)c(c45)ccc(=O)n5C | ([Bromidge et al., 2010](#_ENREF_5)) | 6,310 | 5.20 | 5.69 | b |
|  | c1cc(C)nc(c12)cc(F)cc2N(C[C@@H]3C)CCN3CCc(c(C)cc4)c(c45)ccc(=O)n5C | ([Bromidge et al., 2010](#_ENREF_5)) | 1,259 | 5.90 | 5.84 | a |
|  | c1cc(C)nc(c12)cccc2N(C3)CCN(C3(C)C)CCc(ccc4)c(c45)ccc(=O)n5C | ([Bromidge et al., 2010](#_ENREF_5)) | 2,512 | 5.60 | 6.12 | b |
|  | c1cc(C)nc(c12)cccc2C(CC3)CCN3CCc(c(C)cc4)c(c45)ccc(=O)n5C | ([Bromidge et al., 2010](#_ENREF_5)) | 1,995 | 5.70 | 5.52 | a |
|  | c1ccc(c2c13)cccc2N(S3(=O)=O)CCCN(CC4)CCN4c(cc5)ccc5F  **Fananserin** | ([Fletcher et al., 2002](#_ENREF_16)) | 200 | 6.70 | 6.75 | a |
|  | c1cccc(CC2)c1[C@H](O)C23CCN(CC3)CCc4ccccc4 | ([Fletcher et al., 2002](#_ENREF_16)) | 1,200 | 5.92 | 6.15 | b |
|  | c1cccc(CC2)c1OC23CCN(CC3)CCc(c(F)c4)ccc4F | ([Fletcher et al., 2002](#_ENREF_16)) | 110 | 6.96 | 6.76 | b,* |
|  | c1cccc(CC2)c1CC23CCN(CC3)CCc(c(F)c4)ccc4F | ([Fletcher et al., 2002](#_ENREF_16)) | 69 | 7.16 | 6.90 | b,* |
|  | c1cccc(CC2)c1S(=O)(=O)C23CCN(CC3)CCc(c(F)c4)ccc4F | ([Fletcher et al., 2002](#_ENREF_16)) | 570 | 6.24 | 6.01 | b |
|  | c1ccccc1S(=O)(=O)C2CCN(CC2)CCc(c(F)c3)ccc3F | ([Fletcher et al., 2002](#_ENREF_16)) | 1,000 | 6.00 | 5.87 | b |
|  | c1ccccc1S(=O)(=O)C2CCN(CC2)CCc3ccccc3 | ([Fletcher et al., 2002](#_ENREF_16)) | 1,800 | 5.74 | 5.75 | b |
|  | c1ccccc1S(=O)(=O)C2CCN(CC2)CCCc3ccccc3 | ([Fletcher et al., 2002](#_ENREF_16)) | 2,500 | 5.60 | 5.58 | a |
|  | c1cc(C#N)ccc1S(=O)(=O)C(CC2)CCN2CCc(cc3)c(F)cc3F | ([Fletcher et al., 2002](#_ENREF_16)) | 560 | 6.25 | 6.06 | a |
|  | c1ccc(C#N)cc1S(=O)(=O)C(CC2)CCN2CCc(c(F)c3)ccc3F | ([Fletcher et al., 2002](#_ENREF_16)) | 1,900 | 5.72 | 5.64 | b |
|  | c1cccc(c1C#N)S(=O)(=O)C(CC2)CCN2CCc(c(F)c3)ccc3F | ([Fletcher et al., 2002](#_ENREF_16)) | 4,000 | 5.40 | 5.37 | a |
|  | Fc1ccc(cc1)S(=O)(=O)C(CC2)CCN2CCc(cc3)c(F)cc3F | ([Fletcher et al., 2002](#_ENREF_16)) | 710 | 6.15 | 6.25 | a |
|  | Clc1ccc(cc1)S(=O)(=O)C(CC2)CCN2CCc(cc3)c(F)cc3F | ([Fletcher et al., 2002](#_ENREF_16)) | 150 | 6.82 | 6.42 | a,* |
|  | O=C(N)c1ccc(cc1)S(=O)(=O)C(CC2)CCN2CCc(cc3)c(F)cc3F | ([Fletcher et al., 2002](#_ENREF_16)) | 1,300 | 5.89 | 5.83 | b |
|  | O=C(N)c1cccc(c1)S(=O)(=O)C(CC2)CCN2CCc(cc3)c(F)cc3F | ([Fletcher et al., 2002](#_ENREF_16)) | 7,143 | 5.15 | 5.55 | b |
|  | c1ccccc1S(=O)(=O)C(CC2)CCN2CC(=O)c(cc3)ccc3F | ([Fletcher et al., 2002](#_ENREF_16)) | 6,800 | 5.17 | 5.25 | b |
|  | C1NC[C@@H]([C@@H]12)CN(C2)c(n3)n(c(c34)cccc4)C(CC5)CCN5C6(CO)CCCCCCC6  **HPCOM** | ([Hayashi et al., 2010](#_ENREF_18)) | 3,700 | 5.43 | 5.52 | a |
|  | o1cccc1-c2ccc(cc2)OCCCN3CCCCC3 | ([Levoin et al., 2011](#_ENREF_20)) | 90 | 7.05 | 6.65 | a,* |
|  | C1CCO[C@H]1c2ccc(cc2)OCCCN3CCCCC3 | ([Levoin et al., 2011](#_ENREF_20)) | 2,747 | 5.56 | 5.77 | a |
|  | [nH]1ccnc1-c2ccc(cc2)OCCCN3CCCCC3 | ([Levoin et al., 2011](#_ENREF_20)) | 10,000 | 5.00 | 5.28 | a |
|  | c1cnn(C)c1-c2ccc(cc2)OCCCN3CCCCC3 | ([Levoin et al., 2011](#_ENREF_20)) | 190 | 6.72 | 6.50 | a |
|  | o1cncc1-c2ccc(cc2)OCCCN3CCCCC3 | ([Levoin et al., 2011](#_ENREF_20)) | 1,194 | 5.92 | 5.56 | a |
|  | O1CCN=C1c2ccc(cc2)OCCCN3CCCCC3 | ([Levoin et al., 2011](#_ENREF_20)) | 10,000 | 5.00 | 5.61 | a |
|  | C1CCC=C1c2ccc(cc2)OCCCN3CCCCC3 | ([Levoin et al., 2011](#_ENREF_20)) | 230 | 6.64 | 6.55 | b |
|  | C1CCCCN1c2ccc(cc2)OCCCN3CCCCC3 | ([Levoin et al., 2011](#_ENREF_20)) | 841 | 6.07 | 6.03 | a |
|  | O1CCCC[C@H]1c2ccc(cc2)OCCCN3CCCCC3 | ([Levoin et al., 2011](#_ENREF_20)) | 1,125 | 5.95 | 6.22 | a |
|  | C1COCCC1c2ccc(cc2)OCCCN3CCCCC3 | ([Levoin et al., 2011](#_ENREF_20)) | 2,220 | 5.65 | 5.94 | b |
|  | COc1ccccc1-c2ccc(cc2)OCCCN3CCCCC3 | ([Levoin et al., 2011](#_ENREF_20)) | 76 | 7.12 | 7.05 | a,* |
|  | C1CCCCN1C(=O)c2ccccc2-c3ccc(cc3)OCCCN4CCCCC4 | ([Levoin et al., 2011](#_ENREF_20)) | 818 | 6.09 | 6.04 | a |
|  | CN(C)[C@H]1CC[C@@H](CC1)c2ccc(cc2)OCCCN3CCCCC3 | ([Levoin et al., 2011](#_ENREF_20)) | 10,000 | 5.00 | 5.17 | a |
|  | O=S(=O)(N)NCCC(CC1)CCN1c(ncn2)c(c23)cc(OC)c(c3)OC | ([Patel et al., 2009](#_ENREF_30)) | 601 | 6.22 | 6.14 | b |
|  | O=S(=O)(N)NCCC(CC1)CCN1c(ncn2)c(c23)ccc(c3)OC | ([Patel et al., 2009](#_ENREF_30)) | 120 | 6.92 | 6.22 | b,* |
|  | O=S(=O)(N)NCCC(CC1)CCN1c(ncn2)c(c23)ccc(Cl)c3 | ([Patel et al., 2009](#_ENREF_30)) | 3,400 | 5.47 | 5.50 | b |
|  | O=S(=O)(N)NCCC(CC1)CCN1c(ncn2)c(c23)cc(Cl)cc3 | ([Patel et al., 2009](#_ENREF_30)) | 5,420 | 5.27 | 5.35 | a |
|  | O=S(=O)(N)NCCC(CC1)CCN1c(c(c23)cc(OC)c(c3)OC)nc(n2)/C=C/c4cccnc4 | ([Patel et al., 2009](#_ENREF_30)) | 276 | 6.56 | 6.23 | b |
|  | O=S(=O)(N)NCCC(CC1)CCN1C2=NC(=NCC2)n3ccnc3C | ([Patel et al., 2009](#_ENREF_30)) | 6,867 | 5.16 | 4.98 | a |
|  | COc1cccc(c1OC)[C@H](O)C2CCN(CC2)CCc(cc3)ccc3F  **M100907** | ([Fish et al., 2005](#_ENREF_14)) | 1,100 | 5.96 | 6.28 | b |
|  | c1ccccc1S(=O)(=O)C2CCN(CC2)CCc(c(F)c3)ccc3F | ([Fish et al., 2005](#_ENREF_14)) | 1,000 | 6.00 | 5.88 | b |
|  | c1cc(F)ccc1S(=O)(=O)C2CCN(CC2)CCc(cc3)c(F)cc3F | ([Fish et al., 2005](#_ENREF_14)) | 710 | 6.15 | 6.25 | a |
|  | c1ccccc1S(=O)(=O)C2CCN(CC2)CC(=O)c(c(F)c3)ccc3F | ([Fish et al., 2005](#_ENREF_14)) | 6,800 | 5.17 | 5.28 | a |
|  | c1ccccc1S(=O)(=O)C2(F)CCN(CC2)CCc(c(F)c3)ccc3F | ([Fish et al., 2005](#_ENREF_14)) | 2,446 | 5.61 | 5.75 | b |
|  | c1cc(F)ccc1S(=O)(=O)C2(F)CCN(CC2)CCc(cc3)c(F)cc3F | ([Fish et al., 2005](#_ENREF_14)) | 5,561 | 5.25 | 5.73 | a |
|  | c1ccc(F)cc1S(=O)(=O)C2(F)CCN(CC2)CCc(cc3)c(F)cc3F | ([Fish et al., 2005](#_ENREF_14)) | 1,525 | 5.82 | 5.73 | a |
|  | Fc1ccccc1S(=O)(=O)C2(F)CCN(CC2)CCc(c(F)c3)ccc3F | ([Fish et al., 2005](#_ENREF_14)) | 398 | 6.40 | 5.85 | a |
|  | c1cc(Cl)ccc1S(=O)(=O)C2(F)CCN(CC2)CCc(c(F)c3)ccc3F | ([Fish et al., 2005](#_ENREF_14)) | 397 | 6.40 | 5.80 | a |
|  | c1cc(C#N)ccc1S(=O)(=O)C2(F)CCN(CC2)CCc(cc3)c(F)cc3F | ([Fish et al., 2005](#_ENREF_14)) | 2,702 | 5.57 | 5.49 | b |
|  | C1CCN1c(cc2)ccc2S(=O)(=O)C3(F)CCN(CC3)CCc(cc4)c(F)cc4F | ([Fish et al., 2005](#_ENREF_14)) | 9,971 | 5.00 | 5.29 | a |
|  | Fc1ccc(cc1)S(=O)(=O)N2CCN(CC2)CCc(cc3)c(F)cc3F | ([Ladduwahetty et al., 2006](#_ENREF_19)) | 4,869 | 5.31 | 5.54 | a |
|  | N#Cc1c[nH]c(c12)c(ccc2)C(=O)N3CCN(CC3)CCc(cc4)c(F)cc4F  **EMD** | ([Ladduwahetty et al., 2006](#_ENREF_19)) | 1,268 | 5.89 | 5.68 | b |
|  | Fc1ccc(cc1)Cn(c(c23)cccc2)c(n3)N(CC4)CCC4N(C)c([nH]5)nccc5=O | ([Coon et al., 2009](#_ENREF_8)) | 7,258 | 5.14 | 5.61 | a |
|  | Fc1ccc(cc1)Cn(c(c23)cccc2)c(n3)N(CC4)CCC4N(C)C | ([Coon et al., 2009](#_ENREF_8)) | 57 | 7.24 | 6.71 | a,* |
|  | Cc1ccc(cc1)Cn(c(c23)cccc2)c(n3)N(CC4)CCC4N(C)C | ([Coon et al., 2009](#_ENREF_8)) | 73 | 7.14 | 6.88 | a,* |
|  | COc1ccc(cc1)Cn(c(c23)cccc2)c(n3)N(CC4)CCC4N(C)C | ([Coon et al., 2009](#_ENREF_8)) | 158 | 6.80 | 6.74 | b |
|  | COc1ccc(cc1)Cn(c(c23)cccc2)c(n3)N(CC4)CCC4N(C)CC5CCOCC5 | ([Coon et al., 2009](#_ENREF_8)) | 102 | 6.99 | 7.40 | b,* |
|  | Fc1ccc(cc1)Cn(c(c23)cccc2)c(n3)N(CC4)CCC4N(C)CCO | ([Coon et al., 2009](#_ENREF_8)) | 116 | 6.94 | 6.61 | b,* |
|  | Fc1ccc(cc1)Cn(c(c23)cccc2)c(n3)N(CC4)CCC4N(C)CCN(C)C | ([Coon et al., 2009](#_ENREF_8)) | 776 | 6.11 | 6.69 | a |
|  | Fc1ccc(cc1)Cn(c(c23)cccc2)c(n3)N(CC4)CCC4NCC5CCOCC5 | ([Coon et al., 2009](#_ENREF_8)) | 426 | 6.37 | 6.22 | a |
|  | COc1ccc(cc1)Cn(c(c23)cccc2)c(n3)N(CC4)CCC4NCC5CCOCC5 | ([Coon et al., 2009](#_ENREF_8)) | 1,461 | 5.83 | 6.24 | a |
|  | Fc1ccc(cc1)Cn(c(c23)cccc2)c(n3)N(CC4)CCC4n5cccc5 | ([Coon et al., 2009](#_ENREF_8)) | 157 | 6.80 | 6.47 | a |
|  | Fc1ccc(cc1)Cn(c(c23)cccc2)c(n3)N(CC4)CCC4n5ccnc5 | ([Coon et al., 2009](#_ENREF_8)) | 137 | 6.86 | 6.35 | a,* |
|  | Fc1ccc(cc1)Cn(c(c23)cccc2)c(n3)N(CC4)CCC4n5cccn5 | ([Coon et al., 2009](#_ENREF_8)) | 3,536 | 5.45 | 5.94 | a |
|  | Fc1ccc(cc1)Cn(c(c23)cccc2)c(n3)N(CC4)CCC4n5cc(cn5)C | ([Coon et al., 2009](#_ENREF_8)) | 3,849 | 5.41 | 5.92 | a |
|  | C1CCCN1CCc(sc(c23)cccc2)c3[C@H](C)c4ccccn4 | ([Moree et al., 2010](#_ENREF_26)) | 1,100 | 5.96 | 5.98 | b |
|  | c1cccc(c12)sc(CCN3CCC3)c2[C@@H](C)c4ncccc4 | ([Moree et al., 2010](#_ENREF_26)) | 4,200 | 5.38 | 5.38 | a |
|  | c1cccc(c12)sc(CCN(C)C)c2[C@H](C)c3ccccn3 | ([Moree et al., 2010](#_ENREF_26)) | 1,800 | 5.74 | 5.70 | b |
|  | c1cccc(c12)sc(CCN(C)C)c2[C@@H](C)c(cc3)ncc3F | ([Moree et al., 2010](#_ENREF_26)) | 2,500 | 5.60 | 5.82 | b |
|  | c1ccccc1-c(cc2)ccc2C(=O)Nc(cc3)cnc3N4CC[C@@H](C4)NC | ([Dyck et al., 2006](#_ENREF_11)) | 200 | 6.69 | 6.22 | b |
|  | C1COCCC1Cn2cc(c(c23)cccc3CC)-c4scc(n4)CN5CCCC5 | ([Ratcliffe et al., 2011](#_ENREF_32)) | 1,000 | 6.00 | 6.21 | b |
|  | CCN(CC)Cc(n1)csc1-c(c(c23)cccc2CC)cn3CC4CCOCC4 | ([Ratcliffe et al., 2011](#_ENREF_32)) | 1,259 | 5.90 | 5.81 | a |
|  | C1COCCC1Cn2cc(c(c23)cccc3F)-c4scc(n4)CN5CCCC5 | ([Ratcliffe et al., 2011](#_ENREF_32)) | 1,585 | 5.80 | 5.97 | b |
|  | CCN(CC)Cc(n1)csc1-c(c(c23)cccc2F)cn3CC4CCOCC4 | ([Ratcliffe et al., 2011](#_ENREF_32)) | 794 | 6.10 | 5.84 | b |
|  | C1COCCC1Cn2cc(c(c23)cccc3Cl)-c4scc(n4)CN5CCCC5 | ([Ratcliffe et al., 2011](#_ENREF_32)) | 794 | 6.10 | 6.00 | a |
|  | CCN(CC)Cc(n1)csc1-c(c(c23)cccc2Cl)cn3CC4CCOCC4 | ([Ratcliffe et al., 2011](#_ENREF_32)) | 631 | 6.20 | 6.04 | a |
|  | OCCNCc(n1)c(CC)sc1-c(c(c23)cccc2Cl)cn3CC4CCOCC4 | ([Ratcliffe et al., 2011](#_ENREF_32)) | 631 | 6.20 | 6.05 | b |
|  | C1COCCC1Cn2cc(c(c23)cccc3Cl)-c4sc(CC)c(n4)CCNCCO | ([Ratcliffe et al., 2011](#_ENREF_32)) | 316 | 6.50 | 6.56 | a |
|  | C1COCCC1Cn2cc(c(c23)cccc3Cl)-c4sc(CC)c(n4)C[C@@H](C)NCCO | ([Ratcliffe et al., 2011](#_ENREF_32)) | 251 | 6.60 | 6.67 | b |
|  | C1COCCC1Cn2cc(c(c23)cccc3Cl)-c4sc(Cl)c(n4)C[C@@H](C)NCCO | ([Ratcliffe et al., 2011](#_ENREF_32)) | 631 | 6.20 | 6.44 | a |
|  | C1CCCCC1Cn2cc(c(c23)cccc3Cl)-c4scc(n4)CNCCOC | ([Ratcliffe et al., 2011](#_ENREF_32)) | 251 | 6.60 | 6.53 | a |
|  | C1COCCC1Cn2cc(c(c23)cccc3Cl)-c4scc(n4)CNCCOC | ([Ratcliffe et al., 2011](#_ENREF_32)) | 1,000 | 6.00 | 5.91 | a |
|  | OCCNCc(n1)csc1-c(c(c23)cccc2Cl)cn3CC4CCOCC4 | ([Ratcliffe et al., 2011](#_ENREF_32)) | 1,995 | 5.70 | 5.58 | a |
|  | C1COCCC1Cn2cc(c(c23)cccc3Cl)-c4scc(n4)CCNCCO | ([Ratcliffe et al., 2011](#_ENREF_32)) | 794 | 6.10 | 6.19 | a |
|  | C1COCCC1Cn2cc(c(c23)cccc3Cl)-c4scc(n4)C[C@H](C)NCCO | ([Ratcliffe et al., 2011](#_ENREF_32)) | 1,000 | 6.00 | 6.22 | b |
|  | C1COCCC1Cn2cc(c(c23)cccc3Cl)-c4scc(n4)CN(CC5)CCC5O | ([Ratcliffe et al., 2011](#_ENREF_32)) | 2,512 | 5.60 | 5.71 | a |
|  | C1COCCC1Cn2cc(c(c23)cccc3Cl)-c4scc(n4)CN(CC5)CCC5OC | ([Ratcliffe et al., 2011](#_ENREF_32)) | 1,585 | 5.80 | 5.96 | a |
|  | C1COCCC1Cn2cc(c(c23)cccc3Cl)-c4scc(n4)CN5CCOCC5 | ([Ratcliffe et al., 2011](#_ENREF_32)) | 3,981 | 5.40 | 5.85 | b |
|  | OCCN(CCO)Cc(n1)csc1-c(c(c23)cccc2Cl)cn3CC4CCOCC4 | ([Ratcliffe et al., 2011](#_ENREF_32)) | 2,512 | 5.60 | 5.93 | b |
|  | c1c(Cl)ccc(c12)[nH]c(=O)cc2NC3CCN(CC3)Cc(c4)ccc(c45)OCO5 | ([Blackburn et al., 2006](#_ENREF_3)) | 112 | 6.95 | 7.24 | a,* |
|  | c1cc(Cl)cc(c12)c(cc(=O)n2C)NC3CCN(CC3)Cc(c4)ccc(c45)cccc5 | ([Blackburn et al., 2006](#_ENREF_3)) | 215 | 6.67 | 6.70 | a |
|  | COc(c1)ccc(c12)n(C)c(=O)cc2NC3CCN(CC3)Cc(c4)ccc(c45)OCO5 | ([Blackburn et al., 2006](#_ENREF_3)) | 778 | 6.11 | 5.87 | a |
|  | FC(F)(F)c(c1)ccc(c12)n(C)c(=O)cc2NC3CCN(CC3)Cc(c4)ccc(c45)OCO5 | ([Blackburn et al., 2006](#_ENREF_3)) | 1,800 | 5.74 | 5.67 | a |
|  | c1ccccc1Cn2c(=O)cc(c(c23)cc(Cl)cc3)NC4CCN(CC4)Cc(c5)ccc(c56)OCO6 | ([Blackburn et al., 2006](#_ENREF_3)) | 280 | 6.55 | 6.45 | b |
|  | c1cnccc1Cn(c(c23)ccc(Cl)c2)c(=O)cc3NC4CCN(CC4)Cc(c5)ccc(c56)OCO6 | ([Blackburn et al., 2006](#_ENREF_3)) | 355 | 6.45 | 6.57 | b |
|  | c1cccnc1Cn2c(=O)cc(c(c23)cc(Cl)cc3)NC4CCN(CC4)Cc(c5)ccc(c56)cccc6 | ([Blackburn et al., 2006](#_ENREF_3)) | 88 | 7.06 | 6.90 | b,* |
|  | n1ccccc1Cn(c(c23)ccc(Cl)c2)c(=O)cc3NC4CCN(CC4)Cc(c5)ccc(c56)OCO6 | ([Blackburn et al., 2006](#_ENREF_3)) | 550 | 6.26 | 6.47 | b |
|  | c1cc(Cl)cc(c12)c(c(C#N)c(=O)[nH]2)NC3CCN(CC3)Cc(c4)ccc(c45)OCO5 | ([Blackburn et al., 2006](#_ENREF_3)) | 420 | 6.38 | 6.80 | b |
|  | c1cc(Cl)cc(c12)c(c(c(=O)n2C)C(=O)OC)NC3CCN(CC3)Cc(c4)ccc(c45)OCO5 | ([Blackburn et al., 2006](#_ENREF_3)) | 1,440 | 5.84 | 6.33 | b |
|  | c1cc(Cl)cc(c12)c(nc(=O)n2C)NC3CCN(CC3)Cc(c4)ccc(c45)cccc5 | ([Blackburn et al., 2006](#_ENREF_3)) | 160 | 6.79 | 6.54 | a |
|  | c1c(Cl)ccc(c12)n(C)c(=O)nc2NC3CCN(CC3)Cc(c4)ccc(c45)OCO5 | ([Blackburn et al., 2006](#_ENREF_3)) | 360 | 6.44 | 6.61 | b |
|  | c1cc(Cl)cc(c12)c(nc(=O)n2C)NC3CCN(CC3)Cc(c4)ccc(c45)nccc5 | ([Blackburn et al., 2006](#_ENREF_3)) | 1750 | 5.76 | 6.31 | a |
|  | FC(F)(F)Cn1c(=O)nc(c(c12)cc(Cl)cc2)NC3CCN(CC3)Cc(c4)ccc(c45)cccc5 | ([Blackburn et al., 2006](#_ENREF_3)) | 150 | 6.84 | 6.89 | b,* |
|  | c1c(Cl)ccc(c12)n(CC(F)(F)F)c(=O)nc2NC3CCN(CC3)Cc(c4)ccc(c45)OCO5 | ([Blackburn et al., 2006](#_ENREF_3)) | 445 | 6.35 | 6.63 | b |
|  | c1c(Cl)ccc(c12)n(CCCCC)c(=O)nc2NC3CCN(CC3)Cc(c4)ccc(c45)OCO5 | ([Blackburn et al., 2006](#_ENREF_3)) | 135 | 6.87 | 6.76 | b,* |
|  | c1cc(Cl)cc(c12)c(nc(=O)n2C)N[C@@H](C[C@@H]34)C[C@H](CC3)N4c(c5)ccc(c56)OCO6 | ([Blackburn et al., 2006](#_ENREF_3)) | 540 | 6.27 | 6.55 | b |
|  | c1c(Cl)ccc(c12)n(CC(F)(F)F)c(=O)nc2N[C@H](C[C@H]34)C[C@@H](CC3)N4c(c5)ccc(c56)OCO6 | ([Blackburn et al., 2006](#_ENREF_3)) | 120 | 6.92 | 6.55 | b,* |
|  | c1c(Cl)ccc(c12)n(C)c(=O)nc2N[C@H](C[C@@H]34)C[C@H](CC3)N4c(c5)ccc(c56)OCO6 | ([Blackburn et al., 2006](#_ENREF_3)) | 310 | 6.51 | 6.16 | b |
|  | c1ccc(Cl)cc1COc(c(Cl)c2)ccc2C(=O)N3CC(=O)N(C)CC3 | ([Montgomery et al., 2009](#_ENREF_25)) | 37,200 | 4.43 | 4.28 | a |
|  | c1ccc(Cl)cc1COc(c(c2)C)ccc2C(=O)N3CCN(CC3)C(=O)[C@H]4OCCC4 | ([Montgomery et al., 2009](#_ENREF_25)) | 23,900 | 4.62 | 5.24 | b |
|  | c1cccc(c12)n(CC(=O)NC)c(=O)n2C3CCN(CC3)CC[C@@H](C(C)C)Oc4cc(OC)ccc4C | ([Palin et al., 2009](#_ENREF_29)) | 56 | 7.25 | 7.52 | a,* |
|  | c1cccc(c12)n(CC(=O)NC)c(=O)n2C3CCN(CC3)[C@@H]4CC[C@@H](CC4)C(C)C | ([Palin et al., 2009](#_ENREF_29)) | 4,200 | 5.38 | 5.41 | a |
|  | C1C[C@@H](N2)C[C@@H](C[C@H]12)Oc(cc(c3)C#N)nc3-c4ccccc4 | ([Angus et al., 2011](#_ENREF_1)) | 119 | 6.92 | 6.75 | a,* |
|  | C1C[C@@H](N2)C[C@@H](C[C@H]12)Oc(ccc3)nc3-c4ccccc4C#N | ([Angus et al., 2011](#_ENREF_1)) | 7,080 | 5.15 | 5.69 | a |
|  | C1C[C@@H](N2)C[C@@H](C[C@H]12)Oc(cc(c3)C#N)cc3-c4ccccn4 | ([Angus et al., 2011](#_ENREF_1)) | 1,621 | 5.79 | 6.14 | b |
|  | C1C[C@@H](N2)C[C@@H](C[C@H]12)Oc(cc(c3)C#N)nc3-c4ccccc4C#N | ([Angus et al., 2011](#_ENREF_1)) | 251 | 6.60 | 6.14 | a |
|  | C1C[C@@H](N2)C[C@@H](C[C@H]12)Oc(cc(c3)C#N)cc3-c4ncccc4C#N | ([Angus et al., 2011](#_ENREF_1)) | 3,802 | 5.42 | 6.00 | b |
|  | COc(c1)cnc(c12)c(ccn2)NCc(nn3)n(c34)nc(cc4)-c(c5)cc(F)cc5F | ([Boezio et al., 2009](#_ENREF_4)) | 200 | 6.70 | 6.72 | a |
|  | COc(c1)cnc(c12)c(ccn2)NCc(nn3)n(c34)nc(cc4)-c5cc(ns5)C | ([Boezio et al., 2009](#_ENREF_4)) | 1,600 | 5.79 | 5.67 | b |
|  | N#Cc1ccc(cc1)-c(cc2)cc(c23)cc(o3)CCN4CCC[C@H]4C  **ABT-239** | ([Liu et al., 2010](#_ENREF_23)) | 400 | 6.40 | 6.36 | a |
|  | C1CC[C@@H](C)N1CCc(cc2)cc(c23)ccc(n3)-c(cc4)ccc4C#N | ([Liu et al., 2010](#_ENREF_23)) | 780 | 6.11 | 6.04 | a |
|  | n1cccc(c12)cc(cc2)CCN3[C@H](C)CCC3 | ([Liu et al., 2010](#_ENREF_23)) | 10,000 | 5.00 | 5.40 | b |
|  | C1CC[C@@H](C)N1CCc(cc2)cc(c23)ccc(n3)C4CC4 | ([Liu et al., 2010](#_ENREF_23)) | 11,000 | 4.96 | 5.28 | b |
|  | CC(C)(C)c(n1)ccc(c12)cc(cc2)CCN3[C@H](C)CCC3 | ([Liu et al., 2010](#_ENREF_23)) | 1,700 | 5.77 | 5.45 | a |
|  | C1CC[C@@H](C)N1CCc(cc2)cc(c23)ccc(n3)-c4nccs4 | ([Liu et al., 2010](#_ENREF_23)) | 2,900 | 5.54 | 5.75 | a |
|  | C1CC[C@@H](C)N1CCc(cc2)cc(c23)ccc(n3)C4=NCCS4 | ([Liu et al., 2010](#_ENREF_23)) | 6,300 | 5.20 | 5.34 | b |
|  | C1CC[C@@H](C)N1CCc(cc2)cc(c23)ccc(n3)-c4c(C)nc(s4)C | ([Liu et al., 2010](#_ENREF_23)) | 5,100 | 5.29 | 5.22 | a |
|  | C1CC[C@@H](C)N1CCc(cc2)cc(c23)ccc(n3)-c4c(C)nc(o4)C | ([Liu et al., 2010](#_ENREF_23)) | 10,000 | 5.00 | 5.13 | a |
|  | C1CC[C@@H](C)N1CCc(cc2)cc(c23)ccc(n3)-c4cc(Br)no4 | ([Liu et al., 2010](#_ENREF_23)) | 220 | 6.66 | 5.94 | a |
|  | C1CC[C@@H](C)N1CCc(cc2)cc(c23)ccc(n3)-c4c(c(no4)C)C(=O)OCC | ([Liu et al., 2010](#_ENREF_23)) | 1,500 | 5.82 | 5.57 | a |
|  | C1CC[C@@H](C)N1CCc(cc2)cc(c23)ccc(n3)-c4cc(no4)C(=O)OCC | ([Liu et al., 2010](#_ENREF_23)) | 4,200 | 5.38 | 5.58 | b |
|  | C1CC[C@@H](C)N1CCc(cc2)cc(c23)ccc(n3)-c4[nH]ncc4 | ([Liu et al., 2010](#_ENREF_23)) | 10,000 | 5.00 | 5.11 | b |
|  | C1CC[C@@H](C)N1CCc(cc2)cc(c23)ccc(n3)-c4c[nH]nc4 | ([Liu et al., 2010](#_ENREF_23)) | 10,000 | 5.00 | 5.22 | b |
|  | C1CC[C@@H](C)N1CCc(cc2)cc(c23)ccc(n3)-c4c(C)n[nH]c4 | ([Liu et al., 2010](#_ENREF_23)) | 4,400 | 5.36 | 5.22 | b |
|  | C1CC[C@@H](C)N1CCc(cc2)cc(c23)ccc(n3)-c4c(C)n(C)nc4 | ([Liu et al., 2010](#_ENREF_23)) | 10,000 | 5.00 | 5.27 | a |
|  | C1CC[C@@H](C)N1CCc(cc2)cc(c23)ccc(n3)-c4c(C)nn(c4)C | ([Liu et al., 2010](#_ENREF_23)) | 6,200 | 5.21 | 5.36 | b |
|  | C1CC[C@@H](C)N1CCc(cc2)cc(c23)ccc(n3)-c4c(C)[nH]nc4C | ([Liu et al., 2010](#_ENREF_23)) | 10,000 | 5.00 | 5.35 | b |
|  | C1CC[C@@H](C)N1CCc(cc2)cc(c23)ccc(n3)-c4cocc4 | ([Liu et al., 2010](#_ENREF_23)) | 2,100 | 5.68 | 5.60 | b |
|  | C1CC[C@@H](C)N1CCc(cc2)cc(c23)ccc(n3)-c4cscc4 | ([Liu et al., 2010](#_ENREF_23)) | 1,200 | 5.92 | 5.99 | a |
|  | C1CC[C@@H](C)N1CCc(cc2)cc(c23)ccc(n3)-c4cccs4 | ([Liu et al., 2010](#_ENREF_23)) | 620 | 6.21 | 5.94 | a |
|  | C1CC[C@@H](C)N1CCc(cc2)cc(c23)ccc(n3)-c4ccc(s4)C#N | ([Liu et al., 2010](#_ENREF_23)) | 480 | 6.32 | 6.09 | a |
|  | C1CC[C@@H](C)N1CCc(cc2)cc(c23)ccc(n3)-c4ccc(s4)/C(=N/[H])OCC | ([Liu et al., 2010](#_ENREF_23)) | 1,200 | 5.92 | 6.23 | a |
|  | C1CC[C@@H](C)N1CCc(cc2)cc(c23)ccc(n3)-c4ccccn4 | ([Liu et al., 2010](#_ENREF_23)) | 4,500 | 5.35 | 5.65 | a |
|  | C1CC[C@@H](C)N1CCc(cc2)cc(c23)ccc(n3)-c4cccnc4 | ([Liu et al., 2010](#_ENREF_23)) | 3,000 | 5.52 | 5.42 | b |
|  | C1CC[C@@H](C)N1CCc(cc2)cc(c23)ccc(n3)-c4ccncc4 | ([Liu et al., 2010](#_ENREF_23)) | 6,900 | 5.16 | 5.41 | a |
|  | C1CC[C@@H](C)N1CCc(cc2)cc(c23)ccc(n3)-c(cn4)ccc4Cl | ([Liu et al., 2010](#_ENREF_23)) | 870 | 6.06 | 6.00 | a |
|  | C1CC[C@@H](C)N1CCc(cc2)cc(c23)ccc(n3)-c(cn4)ccc4C | ([Liu et al., 2010](#_ENREF_23)) | 6,600 | 5.18 | 5.48 | a |
|  | C1CC[C@@H](C)N1CCc(cc2)cc(c23)ccc(n3)-c(c(n4)C)ccc4C | ([Liu et al., 2010](#_ENREF_23)) | 5,000 | 5.30 | 5.35 | a |
|  | C1CC[C@@H](C)N1CCc(cc2)cc(c23)ccc(n3)-c(c(n4)C)cc(C#N)c4C | ([Liu et al., 2010](#_ENREF_23)) | 2,100 | 5.68 | 5.65 | b |
|  | C1CC[C@@H](C)N1CCc(cc2)cc(c23)ccc(n3)-c(c(n4)C)cc(c4C)C(=O)N | ([Liu et al., 2010](#_ENREF_23)) | 10,000 | 5.00 | 5.14 | a |
|  | C1CC[C@@H](C)N1CCc(cc2)cc(c23)ccc(n3)-c4c(C(F)(F)F)nccc4O | ([Liu et al., 2010](#_ENREF_23)) | 10,000 | 5.00 | 4.88 | a |
|  | C1CC[C@@H](C)N1CCc(cc2)cc(c23)ccc(n3)-c4cnccn4 | ([Liu et al., 2010](#_ENREF_23)) | 6,200 | 5.21 | 5.63 | b |
|  | C1CC[C@@H](C)N1CCc(cc2)cc(c23)ccc(n3)-c4cncnc4 | ([Liu et al., 2010](#_ENREF_23)) | 10,000 | 5.00 | 5.48 | b |
|  | C1CC[C@@H](C)N1CCc(cc2)cc(c23)ccc(n3)-c(cn4)cnc4OC | ([Liu et al., 2010](#_ENREF_23)) | 5,600 | 5.25 | 5.30 | a |
|  | C1CC[C@@H](C)N1CCc(cc2)cc(c23)ccc(n3)-c(cn4)c(C)nc4N5CCCC5 | ([Liu et al., 2010](#_ENREF_23)) | 2,600 | 5.58 | 5.54 | b |
|  | C1CC[C@@H](C)N1CCc(cc2)cc(c23)ccc(n3)N(CC4)CCC4C | ([Liu et al., 2010](#_ENREF_23)) | 5,600 | 5.25 | 5.94 | b |
|  | C1CC[C@@H](C)N1CCc(cc2)cc(c23)ccc(n3)-c(cc4)ccc4N5CCCCC5 | ([Liu et al., 2010](#_ENREF_23)) | 690 | 6.16 | 6.30 | a |
|  | C1CC[C@@H](C)N1CCc(cc2)cc(c23)ccc(n3)-c(cc4)ccc4-n(cc5)ccc5=O | ([Liu et al., 2010](#_ENREF_23)) | 5,000 | 5.30 | 5.58 | a |
|  | C1CC[C@@H](C)N1CCc(cc2)cc(c23)ccc(n3)-c4cnc(s4)-c5ccccc5 | ([Liu et al., 2010](#_ENREF_23)) | 340 | 6.47 | 6.44 | a |
|  | C1CC[C@@H](C)N1CCc(cc2)cc(c23)ccc(n3)-c4c(C)nc(s4)-c5cccs5 | ([Liu et al., 2010](#_ENREF_23)) | 930 | 6.03 | 6.00 | b |
|  | C1CC[C@@H](C)N1CCc(cc2)cc(c23)ccc(n3)-c4c(C)nc(s4)-c5cnccn5 | ([Liu et al., 2010](#_ENREF_23)) | 7,900 | 5.10 | 5.06 | a |
|  | C1CC[C@@H](C)N1CCc(cc2)cc(c23)ccc(n3)-c4c(C)nc(s4)N5CCOCC5 | ([Liu et al., 2010](#_ENREF_23)) | 5,100 | 5.29 | 5.41 | b |
|  | C1CC[C@@H](C)N1CCc(cc2)cc(c23)ccc(n3)-c4c(C)nc(s4)-c5ncccc5 | ([Liu et al., 2010](#_ENREF_23)) | 2,800 | 5.55 | 5.47 | a |
|  | C1CC[C@@H](C)N1CCc(cc2)cc(c23)ccc(n3)-c4c(C)nc(s4)-c5cnccc5 | ([Liu et al., 2010](#_ENREF_23)) | 1,500 | 5.82 | 5.94 | a |
|  | C1CC[C@@H](C)N1CCc(cc2)cc(c23)ccc(n3)-c4c(C)nc(s4)-c5ccncc5 | ([Liu et al., 2010](#_ENREF_23)) | 1,200 | 5.92 | 6.07 | a |
|  | C1CC[C@@H](C)N1CCc(cc2)cc(c23)ccc(n3)-c4csc(n4)-c5csc(n5)C | ([Liu et al., 2010](#_ENREF_23)) | 3,100 | 5.51 | 5.50 | b |
|  | C1CC[C@@H](C)N1CCc(cc2)cc(c23)ccc(n3)-c4csc(n4)-c5cc(on5)C | ([Liu et al., 2010](#_ENREF_23)) | 710 | 6.15 | 6.02 | b |
|  | C1CC[C@@H](C)N1CCc(cc2)cc(c23)ccc(n3)-c4csc(n4)-c(c5)cc(Cl)nc5Cl | ([Liu et al., 2010](#_ENREF_23)) | 5,100 | 5.29 | 5.33 | b |
|  | C1CC[C@@H](C)N1CCc(cc2)cc(c23)ccc(n3)-c4csc(n4)-c5ccccc5 | ([Liu et al., 2010](#_ENREF_23)) | 400 | 6.40 | 6.19 | a |
|  | C1CC[C@@H](C)N1CCc(cc2)cc(c23)ccc(n3)-c4csc(n4)-c(cc5)cnc5OCC(F)(F)F | ([Liu et al., 2010](#_ENREF_23)) | 380 | 6.42 | 6.46 | a |
|  | C1CC[C@@H](C)N1CCc(cc2)cc(c23)ccc(n3)-c4ccc(s4)-c5sc(C)cc5 | ([Liu et al., 2010](#_ENREF_23)) | 500 | 6.30 | 6.47 | a |
|  | C1CC[C@@H](C)N1CCc(cc2)cc(c23)ccc(n3)-c4c(C)n(nc4)-c(cc5Cl)ccc5Cl | ([Liu et al., 2010](#_ENREF_23)) | 150 | 6.82 | 6.49 | a,* |
|  | C1CC[C@@H](C)N1CCc(cc2)cc(c23)ccc(n3)-c4c(C)n(nc4)-c5ncccc5 | ([Liu et al., 2010](#_ENREF_23)) | 5,000 | 5.30 | 5.33 | a |
|  | C1CC[C@@H](C)N1CCc(cc2)cc(c23)ccc(n3)-c4c(C)n(nc4)-c(n5)cc(C(F)(F)F)cc5C | ([Liu et al., 2010](#_ENREF_23)) | 4,200 | 5.38 | 5.52 | b |
|  | C1CC[C@@H](C)N1CCc(cc2)cc(c23)ccc(n3)-c4c(C)n(nc4)-c(cc5)nnc5OCC | ([Liu et al., 2010](#_ENREF_23)) | 2,600 | 5.58 | 5.35 | a |
|  | C1CC[C@@H](C)N1CCc(cc2)cc(c23)ccc(n3)-c4c(C)n(nc4)-c(cc5)nnc5N(C)C | ([Liu et al., 2010](#_ENREF_23)) | 4,500 | 5.35 | 5.20 | b |
|  | C1CC[C@@H](C)N1CCc(cc2)cc(c23)ccc(n3)-c4c(C)n(nn4)-c(c(F)c5)ccc5F | ([Liu et al., 2010](#_ENREF_23)) | 720 | 6.14 | 6.27 | a |
|  | C1CC[C@@H](C)N1CCc(cc2)cc(c23)ccc(n3)-c4c(C)c(no4)-c(cc5)ccc5Cl | ([Liu et al., 2010](#_ENREF_23)) | 440 | 6.36 | 6.08 | a |
|  | C1CC[C@@H](C)N1CCc(cc2)cc(c23)ccc(n3)-c(c4C)c(no4)-c(cc5)ccc5Cl | ([Liu et al., 2010](#_ENREF_23)) | 400 | 6.40 | 6.28 | a |
|  | C1CC[C@@H](C)N1CCc(cc2)cc(c23)ccc(n3)-c4c(Cl)n(C)nc4-c5ccccc5 | ([Liu et al., 2010](#_ENREF_23)) | 1,500 | 5.82 | 5.60 | a |
|  | C1CC[C@@H](C)N1CCc(cc2)cc(c23)ccc(n3)-c4c(C)onc4-c5ccccc5 | ([Liu et al., 2010](#_ENREF_23)) | 1,600 | 5.80 | 5.52 | a |
|  | C1CC[C@@H](C)N1CCc(cc2)cc(c23)ccc(n3)-c(s4)nc(c45)cccc5 | ([Liu et al., 2010](#_ENREF_23)) | 480 | 6.32 | 6.13 | a |
|  | C1CC[C@@H](C)N1CCc(cc2)cc(c23)ccc(n3)-c(s4)c(C)n(c45)c6c(n5)cccc6 | ([Liu et al., 2010](#_ENREF_23)) | 430 | 6.37 | 6.46 | a |
|  | C1CC[C@@H](C)N1CCc(cc2)cc(c23)ccc(n3)-c(s4)c(C)n(c45)ncn5 | ([Liu et al., 2010](#_ENREF_23)) | 710 | 6.15 | 5.64 | a |
|  | C1CC[C@@H](C)N1CCc(cc2)cc(c23)ccc(n3)-c(c4)nc(n45)SCC5 | ([Liu et al., 2010](#_ENREF_23)) | 10,000 | 5.00 | 5.48 | a |
|  | C1CC[C@@H](C)N1CCc(cc2)cc(c23)ccc(n3)-c(c(n4)C)n(c45)cccc5 | ([Liu et al., 2010](#_ENREF_23)) | 2,000 | 5.70 | 5.54 | b |
|  | C1CC[C@@H](C)N1CCc(cc2)cc(c23)ccc(n3)-c(cc4)cc(c45)nccn5 | ([Liu et al., 2010](#_ENREF_23)) | 2,100 | 5.68 | 5.66 | b |
|  | C1CC[C@@H](C)N1CCc(cc2)cc(c23)ccc(n3)-c(c(n4)C)cc(c45)cccn5 | ([Liu et al., 2010](#_ENREF_23)) | 10,000 | 5.00 | 5.20 | b |
|  | C1CC[C@@H](C)N1CCc(cc2)cc(c23)ccc(n3)-c(cc4)cc(c45)COCO5 | ([Liu et al., 2010](#_ENREF_23)) | 910 | 6.04 | 5.70 | b |
|  | C1CC[C@@H](C)N1CCc(cc2)cc(c23)ccc(n3)-c(cn4)cc(c45)OCO5 | ([Liu et al., 2010](#_ENREF_23)) | 10,000 | 5.00 | 5.14 | a |
|  | C1CC[C@@H](C)N1CCc(cc2)cc(c23)ccc(n3)-c(cn4)c(C)n(c45)nc(c5)C | ([Liu et al., 2010](#_ENREF_23)) | 2,200 | 5.66 | 5.74 | a |
|  | C1CC[C@@H](C)N1CCc(cc2)cc(c23)ccc(n3)-c(cc4)nc(n45)ncn5 | ([Liu et al., 2010](#_ENREF_23)) | 5,200 | 5.28 | 5.50 | a |
|  | Nc1ccnc(c1)N(CC2)CCC2(c3ccccc3)C(=O)N4CCN(CC4)C(=O)OCC | ([Owen et al., 2009](#_ENREF_28)) | 169 | 6.77 | 6.87 | a |
|  | Nc1ccnc(c1)N(CC2)CCC2(c3ccccc3)C(=O)NCc(cc4)ccc4OC | ([Owen et al., 2009](#_ENREF_28)) | 201 | 6.70 | 6.79 | a |
|  | Nc1ccnc(c1)N(CC2)CCC2(c3ccccc3)NC(=O)c(cc4)c(OC)cc4OC | ([Owen et al., 2009](#_ENREF_28)) | 82 | 7.09 | 7.37 | a,* |
|  | Nc1ccnc(c1)N(CC2)CCC2(c3ccccc3)NC(=O)c(cc4)cc(Cl)c4OC | ([Owen et al., 2009](#_ENREF_28)) | 343 | 6.47 | 6.36 | a |
|  | Nc1ccnc(c1)N(CC2)CCC2(c3ccccc3)NC(=O)c4ncccc4 | ([Owen et al., 2009](#_ENREF_28)) | 548 | 6.26 | 6.31 | a |
|  | c1c(N)ccnc1N(CC2)CCC23c4c([C@H](C3)C(=O)N(C)C)cccc4 | ([Owen et al., 2009](#_ENREF_28)) | 4,710 | 5.33 | 5.24 | a |
|  | c1c(N)ccnc1N(C2)CC2(C)c3cccc(c3)OC | ([Owen et al., 2009](#_ENREF_28)) | 1,840 | 5.73 | 5.85 | b |
|  | c1c(N)ccnc1N2CCC(CC2)N(c(nc3)ccc3C)C(=O)c(cc4)ccc4OC | ([Owen et al., 2009](#_ENREF_28)) | 5,030 | 5.30 | 5.05 | a |
|  | c1c(N)ccnc1N2CCC(CC2)N(c3ccccn3)C(=O)c(c4)cccc4OC | ([Owen et al., 2009](#_ENREF_28)) | 9,690 | 5.01 | 4.88 | a |
|  | c1c(N)ccnc1N2CCC(CC2)n(c(c34)nccc4)c(=O)n3-c(cc5)ccc5OC | ([Owen et al., 2009](#_ENREF_28)) | 865 | 6.06 | 5.65 | b |
|  | c1c(N)ccnc1N2CCC(CC2)n(c(c34)nccc4)c(=O)n3-c5ccccc5 | ([Owen et al., 2009](#_ENREF_28)) | 903 | 6.04 | 5.98 | a |
|  | c1c(N)ccnc1N2CCC(CC2)n(c(c34)nccc4)c(=O)n3Cc5ccccc5 | ([Owen et al., 2009](#_ENREF_28)) | 518 | 6.29 | 6.46 | a |
|  | c1c(N)ccnc1N2CCC(CC2)n(c(c34)nccc4)c(=O)n3CC5CC5 | ([Owen et al., 2009](#_ENREF_28)) | 746 | 6.13 | 6.08 | a |
|  | c1cccc(c12)c(ccc2)N(CC3)CCN3CCCOc(c4)ccc(c45)CNC5 | ([Favor et al., 2010](#_ENREF_12)) | 779 | 6.11 | 6.01 | a |
|  | c1cccc(c12)c(ccc2)N(CC3)CCN3CCCCOc(c4)ccc(c45)CNC5 | ([Favor et al., 2010](#_ENREF_12)) | 1,560 | 5.81 | 5.73 | a |
|  | c1cc(F)cc(c12)c(ccc2)N(CC3)CCN3CCCOc(c4)ccc(c45)CNC5 | ([Favor et al., 2010](#_ENREF_12)) | 931 | 6.03 | 5.82 | b |
|  | c1cc(F)cc(c12)c(ccc2)N(CC3)CCN3CCCCOc(c4)ccc(c45)CNC5 | ([Favor et al., 2010](#_ENREF_12)) | 1,680 | 5.77 | 6.00 | b |
|  | c1cccc(c12)c(ccc2)N(CC3)CCN3CCCOc(c4)ccc(c45)CNC5=O | ([Favor et al., 2010](#_ENREF_12)) | 2,810 | 5.55 | 5.60 | b |
|  | c1cccc(c12)c(ccc2)N(CC3)CCN3CCCCOc(c4)ccc(c45)CNC5=O | ([Favor et al., 2010](#_ENREF_12)) | 1,850 | 5.73 | 5.62 | a |
|  | c1cc(F)cc(c12)c(ccc2)N(CC3)CCN3CCCOc(c4)ccc(c45)CNC5=O | ([Favor et al., 2010](#_ENREF_12)) | 3,610 | 5.44 | 5.55 | a |
|  | c1ccc(F)c(c12)c(ccc2)N(CC3)CCN3CCCOc(c4)ccc(c45)CNC5=O | ([Favor et al., 2010](#_ENREF_12)) | 1,290 | 5.89 | 5.71 | a |
|  | c1ccc(F)c(c12)c(ccc2)N(CC3)CCN3CCCCOc(c4)ccc(c45)CNC5=O | ([Favor et al., 2010](#_ENREF_12)) | 2,060 | 5.69 | 5.69 | a |
|  | c1c(F)ccc(c12)c(ccc2)N(CC3)CCN3CCCOc(c4)ccc(c45)CNC5=O | ([Favor et al., 2010](#_ENREF_12)) | 439 | 6.36 | 6.21 | a |
|  | c1c(F)ccc(c12)c(ccc2)N(CC3)CCN3CCCCOc(c4)ccc(c45)CNC5=O | ([Favor et al., 2010](#_ENREF_12)) | 969 | 6.01 | 6.03 | a |
|  | c1c(F)c(F)cc(c12)c(ccc2)N(CC3)CCN3CCCOc(c4)ccc(c45)CNC5=O | ([Favor et al., 2010](#_ENREF_12)) | 907 | 6.04 | 6.15 | b |
|  | c1c(F)c(F)cc(c12)c(ccc2)N(CC3)CCN3CCCCOc(c4)ccc(c45)CNC5=O | ([Favor et al., 2010](#_ENREF_12)) | 1,860 | 5.73 | 5.82 | a |
|  | CN(C)C(=N/[H])\c(cc1)ccc1C(=O)Nc(cc2)c(cc2Cl)C(=O)Nc(nc3)ccc3Cl | ([Zhang et al., 2009](#_ENREF_33)) | 110 | 6.96 | 6.43 | a,* |
|  | CN(C)C(=N/[H])\c(cc1)ccc1C(=O)Nc(cc2)c(cc2OC)C(=O)Nc(nc3)ccc3Cl | ([Zhang et al., 2009](#_ENREF_33)) | 1,800 | 5.74 | 6.01 | b |
|  | CN(C)C(=N/[H])\c(cc1)cc(F)c1C(=O)Nc(cc2)c(cc2Cl)C(=O)Nc(nc3)ccc3Cl | ([Zhang et al., 2009](#_ENREF_33)) | 420 | 6.38 | 6.51 | b |
|  | CN(C)C(=N/[H])\c(cc1)cc(F)c1C(=O)Nc(cc2)c(cc2OC)C(=O)Nc(nc3)ccc3Cl | ([Zhang et al., 2009](#_ENREF_33)) | 2,100 | 5.68 | 5.84 | a |
|  | C1CCCCN1c(cc(cc2)/C(=N/[H])N(C)C)c2C(=O)Nc(cc3)c(cc3OC)C(=O)Nc(nc4)ccc4Cl | ([Zhang et al., 2009](#_ENREF_33)) | 200 | 6.70 | 6.62 | b |
|  | Cc1cc(Cl)c(c(Cl)c1)OCCOc2ncc(cc2)[C@H]3CCNC[C@@H]3C(=O)N(C4CC4)Cc5cc(ccc5Cl)CCOC | ([Aspiotis et al., 2011](#_ENREF_2)) | 200 | 6.70 | 6.27 | b |
|  | Cc1cc(Cl)c(c(Cl)c1)OCCOc2ccc(cc2)[C@H]3CCNC[C@@H]3C(=O)N(C4CC4)Cc(c5)cc(CCCOC)cc5OCC6(CC#N)CC6 | ([Aspiotis et al., 2011](#_ENREF_2)) | 60 | 7.22 | 6.48 | b,* |
|  | Cc1cc(Cl)c(c(Cl)c1)OCCOc2ccc(cc2)[C@H]3CCNC[C@@H]3C(=O)N(C4CC4)Cc(c5)cc(CCCOC)cc5OCCOC | ([Aspiotis et al., 2011](#_ENREF_2)) | 80 | 7.10 | 7.30 | a,* |
|  | Cc1cc(Cl)c(c(Cl)c1)OCCOc2ccc(cc2)[C@H]3CCNC[C@@H]3C(=O)N(C4CC4)Cc(c5)cc(CCCOC)cc5OCCN(C)C | ([Aspiotis et al., 2011](#_ENREF_2)) | 180 | 6.74 | 6.75 | b |
|  | Cc1cc(Cl)c(c(Cl)c1)OCCOc2ccc(cc2)[C@H]3CCNC[C@@H]3C(=O)N(C4CC4)Cc(c5)cc(CCCOC)cc5OCCCNS(=O)(=O)C | ([Aspiotis et al., 2011](#_ENREF_2)) | 80 | 7.10 | 7.46 | a,* |
|  | Cc1cc(Cl)c(c(Cl)c1)OCCOc2ccc(cc2)[C@H]3CCNC[C@@H]3C(=O)N(C4CC4)Cc(c5)cc(CCCOC)cc5OCCCNC(=O)NCC | ([Aspiotis et al., 2011](#_ENREF_2)) | 150 | 6.82 | 6.54 | b,* |
|  | Cc1cc(Cl)c(c(Cl)c1)OCCOc2ccc(cc2)[C@H]3CCNC[C@@H]3C(=O)N(C4CC4)Cc(c5)cc(CCCOC)cc5OCC(C)(C)O | ([Aspiotis et al., 2011](#_ENREF_2)) | 130 | 6.89 | 6.69 | a,* |
|  | Cc1cc(Cl)c(c(Cl)c1)OCCOc2ccc(cc2)[C@H]3CCNC[C@@H]3C(=O)N(C4CC4)Cc(c5)cc(CCCOC)cc5OC[C@@H]6C[C@@H]6C(=O)OCC | ([Aspiotis et al., 2011](#_ENREF_2)) | 80 | 7.10 | 6.78 | b |
|  | Cc1cc(Cl)c(c(Cl)c1)OCCOc2ccc(cc2)[C@H]3CCNC[C@@H]3C(=O)N(C4CC4)Cc(c5)cc(CCCOC)cc5OC[C@@H]6C[C@@H]6C(=O)O | ([Aspiotis et al., 2011](#_ENREF_2)) | 1,100 | 5.96 | 6.20 | a |
|  | Cc1cc(Cl)c(c(Cl)c1)OCCOc2ccc(cc2)[C@H]3CCNC[C@@H]3C(=O)N(C4CC4)Cc(c5)cc(CCCOC)cc5OC[C@@H]6C[C@@H]6C(=O)OCC(=O)N(C)C | ([Aspiotis et al., 2011](#_ENREF_2)) | 200 | 6.70 | 6.83 | a |
|  | Cc1cc(Cl)c(c(Cl)c1)OCCOc2ccc(cc2)[C@@H](CC3)[C@H](CN3C(=O)OCc4c(C)oc(o4)=O)C(=O)N(C5CC5)Cc(c6)cc(CCCOC)cc6OC[C@@H]7C[C@@H]7C(=O)O | ([Aspiotis et al., 2011](#_ENREF_2)) | 3,700 | 5.43 | 5.29 | a |
|  | Cc1cc(Cl)c(c(Cl)c1)OCCOc2ccc(cc2)[C@H]3CCNC[C@@H]3C(=O)N(C4CC4)Cc(c5)cc(CCCOC)cc5OCC6(CC6)CC(=O)O | ([Aspiotis et al., 2011](#_ENREF_2)) | 1,000 | 6.00 | 6.07 | a |
|  | Cc1cc(Cl)c(c(Cl)c1)OCCOc2ccc(cc2)[C@H]3CCNC[C@@H]3C(=O)N(C4CC4)Cc(c5)cc(CCCOC)cc5OCC(C)(C)C(=O)O | ([Aspiotis et al., 2011](#_ENREF_2)) | 600 | 6.22 | 6.09 | b |
|  | Cc1cc(Cl)c(c(Cl)c1)OCCOc2ccc(cc2)[C@H]3CCNC[C@@H]3C(=O)N(C4CC4)Cc(c5)cc(CCCOC)cc5OCCC(C)(C)C(=O)O | ([Aspiotis et al., 2011](#_ENREF_2)) | 400 | 6.40 | 6.71 | a |
|  | Cc1cc(Cl)c(c(Cl)c1)OCCOc2ccc(cc2)[C@H]3CCNC[C@@H]3C(=O)N(C4CC4)Cc(c5)cc(CCCOC)cc5OCCCC(C)(C)C(=O)O | ([Aspiotis et al., 2011](#_ENREF_2)) | 200 | 6.70 | 6.47 | b |
|  | Cc1cc(Cl)c(c(Cl)c1)OCCOc2ccc(cc2)[C@H]3CCNC[C@@H]3C(=O)N(C4CC4)Cc(c5)cc(CCCOC)cc5OCCC(CC)(CC)C(=O)O | ([Aspiotis et al., 2011](#_ENREF_2)) | 400 | 6.40 | 5.98 | b |
|  | Cc1cc(Cl)c(c(Cl)c1)OCCOc2ccc(cc2)[C@H]3CCNC[C@@H]3C(=O)N(C4CC4)Cc(c5)cc(CCCOC)cc5OCCC6(C(=O)O)CCCC6 | ([Aspiotis et al., 2011](#_ENREF_2)) | 500 | 6.30 | 6.49 | b |
|  | Cc1cc(Cl)c(c(Cl)c1)OCCOc2ccc(cc2)[C@H]3CCNC[C@@H]3C(=O)N(C4CC4)Cc(c5)cc(CCCOC)cc5OCC[C@](C)(C(=O)O)OC | ([Aspiotis et al., 2011](#_ENREF_2)) | 2,300 | 5.64 | 5.80 | a |
|  | Cc1cc(Cl)c(c(Cl)c1)OCCOc2ccc(cc2)[C@H]3CCNC[C@@H]3C(=O)N(C4CC4)Cc(c5)cc(CCCOC)cc5OC[C@@H]6[C@@H](C6)C(=O)NS(=O)(=O)C | ([Aspiotis et al., 2011](#_ENREF_2)) | 500 | 6.30 | 6.53 | b |
|  | Cc1cc(Cl)c(c(Cl)c1)OCCOc2ccc(cc2)[C@H]3CCNC[C@@H]3C(=O)N(C4CC4)Cc(c5)cc(CCCOC)cc5OCC6(CC6)Cc7n[nH]nn7 | ([Aspiotis et al., 2011](#_ENREF_2)) | 500 | 6.30 | 6.02 | a |
|  | C1SCCN1C(=O)[C@@H](N)[C@@H]2CC[C@@H](CC2)NC(=O)c(cc3F)ccc3F | ([Caldwell et al., 2004](#_ENREF_7)) | 5,900 | 5.23 | 4.78 | a |
|  | C1CCCN1C(=O)[C@@H](N)[C@@H]2CC[C@@H](CC2)NC(=O)c(cc3F)ccc3F | ([Caldwell et al., 2004](#_ENREF_7)) | 29,000 | 4.54 | 4.74 | a |
|  | C1C[C@H](F)CN1C(=O)[C@@H](N)[C@@H]2CC[C@@H](CC2)NC(=O)c(cc3F)ccc3F | ([Caldwell et al., 2004](#_ENREF_7)) | 19,000 | 4.72 | 4.76 | a |
|  | FC1(F)CN(CC1)C(=O)[C@@H](N)[C@@H]2CC[C@@H](CC2)NC(=O)c(cc3F)ccc3F | ([Caldwell et al., 2004](#_ENREF_7)) | 16,000 | 4.80 | 4.79 | b |
|  | C1CSCN1C(=O)[C@@H](N)[C@@H]2CC[C@@H](CC2)NC(=O)c3ccccc3C(F)(F)F | ([Caldwell et al., 2004](#_ENREF_7)) | 39,000 | 4.41 | 4.32 | a |
|  | C1CCCN1C(=O)[C@@H](N)[C@@H]2CC[C@@H](CC2)NC(=O)OCc3ccccc3 | ([Caldwell et al., 2004](#_ENREF_7)) | 40,000 | 4.40 | 4.38 | a |
|  | C1C[C@H](F)CN1C(=O)[C@@H](N)[C@@H]2CC[C@@H](CC2)NC(=O)OCc3ccccc3 | ([Caldwell et al., 2004](#_ENREF_7)) | 37,000 | 4.43 | 4.35 | a |
|  | C1C[C@@H](F)CN1C(=O)[C@@H](N)[C@@H]2CC[C@@H](CC2)NC(=O)OCc3ccccc3 | ([Caldwell et al., 2004](#_ENREF_7)) | 43,000 | 4.37 | 4.77 | b |
|  | FC1(F)CN(CC1)C(=O)[C@@H](N)[C@@H]2CC[C@@H](CC2)NC(=O)OCc3ccccc3 | ([Caldwell et al., 2004](#_ENREF_7)) | 42,000 | 4.38 | 4.23 | a |
|  | C1CSCN1C(=O)[C@@H](N)[C@@H]2CC[C@@H](CC2)NS(=O)(=O)c(cc3)ccc3OC(F)(F)F | ([Caldwell et al., 2004](#_ENREF_7)) | 2,800 | 5.55 | 5.59 | a |
|  | C1CCCN1C(=O)[C@@H](N)[C@@H]2CC[C@@H](CC2)NS(=O)(=O)c(cc3)ccc3OC(F)(F)F | ([Caldwell et al., 2004](#_ENREF_7)) | 5,700 | 5.24 | 5.14 | b |
|  | C1C[C@H](F)CN1C(=O)[C@@H](N)[C@@H]2CC[C@@H](CC2)NS(=O)(=O)c(cc3)ccc3OC(F)(F)F | ([Caldwell et al., 2004](#_ENREF_7)) | 5,500 | 5.26 | 5.08 | b |
|  | C1C[C@@H](F)CN1C(=O)[C@@H](N)[C@@H]2CC[C@@H](CC2)NS(=O)(=O)c(cc3)ccc3OC(F)(F)F | ([Caldwell et al., 2004](#_ENREF_7)) | 6,000 | 5.22 | 5.30 | b |
|  | FC1(F)CN(CC1)C(=O)[C@@H](N)[C@@H]2CC[C@@H](CC2)NS(=O)(=O)c(cc3)ccc3OC(F)(F)F | ([Caldwell et al., 2004](#_ENREF_7)) | 5,300 | 5.28 | 5.24 | b |
|  | C1CCCN1C(=O)[C@@H](N)[C@@H]2CC[C@@H](CC2)NS(=O)(=O)c(cc3)c(F)cc3F | ([Caldwell et al., 2004](#_ENREF_7)) | 35,000 | 4.46 | 4.48 | b |
|  | C1C[C@H](F)CN1C(=O)[C@@H](N)[C@@H]2CC[C@@H](CC2)NS(=O)(=O)c(cc3)c(F)cc3F | ([Caldwell et al., 2004](#_ENREF_7)) | 49,000 | 4.31 | 4.45 | a |
|  | C1C[C@@H](F)CN1C(=O)[C@H](N)[C@H]2CC[C@H](CC2)NS(=O)(=O)c(cc3)c(F)cc3F | ([Caldwell et al., 2004](#_ENREF_7)) | 52,000 | 4.28 | 4.30 | a |
|  | c1coc(c12)ccc(c2)-c3ccc(nn3)N(C4)[C@@H](C5)CN([C@H]45)C | ([Li et al., 2010b](#_ENREF_22)) | 8,800 | 5.06 | 4.89 | a |
|  | c1csc(c12)ccc(c2)-c3ccc(nn3)N(C4)[C@@H](C5)CN([C@H]45)C | ([Li et al., 2010b](#_ENREF_22)) | 1,800 | 5.75 | 5.60 | b |
|  | [nH]1ccc(c12)cc(cc2)-c3nnc(cc3)N(C4)[C@@H](C5)CN([C@H]45)C | ([Li et al., 2010b](#_ENREF_22)) | 21,000 | 4.68 | 4.86 | b |
|  | FC(F)(F)c([nH]1)cc(c12)cc(cc2)-c3nnc(cc3)N(C4)[C@@H](C5)CN([C@H]45)C | ([Li et al., 2010b](#_ENREF_22)) | 2,200 | 5.66 | 5.47 | a |
|  | [nH]1ccc(c12)ccc(c2)-c3nnc(cc3)N(C4)[C@@H](C5)CN([C@H]45)C | ([Li et al., 2010b](#_ENREF_22)) | 12,000 | 4.92 | 5.17 | a |
|  | [nH]1c(C)cc(c12)cc(cc2)-c3nnc(cc3)N(C4)[C@@H](C5)CN([C@H]45)C | ([Li et al., 2010b](#_ENREF_22)) | 12,000 | 4.92 | 5.38 | a |
|  | c1ccccc1-c2cnc(cn2)N(C3)[C@@H](C4)CN([C@H]34)C | ([Li et al., 2010b](#_ENREF_22)) | 26,000 | 4.58 | 5.21 | a |
|  | c1coc(c12)ccc(c2)-c3cnc(cn3)N(C4)[C@@H](C5)CN([C@H]45)C | ([Li et al., 2010b](#_ENREF_22)) | 5,300 | 5.28 | 4.92 | b |
|  | c1csc(c12)ccc(c2)-c3cnc(cn3)N(C4)[C@@H](C5)CN([C@H]45)C | ([Li et al., 2010b](#_ENREF_22)) | 1,200 | 5.92 | 5.63 | b |
|  | [nH]1ccc(c12)cc(cc2)-c3ncc(nc3)N(C4)[C@@H](C5)CN([C@H]45)C | ([Li et al., 2010b](#_ENREF_22)) | 14,000 | 4.85 | 4.91 | b |
|  | FC(F)(F)c([nH]1)cc(c12)cc(cc2)-c3ncc(nc3)N(C4)[C@@H](C5)CN([C@H]45)C | ([Li et al., 2010b](#_ENREF_22)) | 1,900 | 5.72 | 5.83 | b |
|  | [nH]1ccc(c12)ccc(c2)-c3ncc(nc3)N(C4)[C@@H](C5)CN([C@H]45)C | ([Li et al., 2010b](#_ENREF_22)) | 9,000 | 5.05 | 5.22 | b |
|  | c1ccccc1-c2cnc(nc2)N(C3)[C@@H](C4)CN([C@H]34)C | ([Li et al., 2010b](#_ENREF_22)) | 22,000 | 4.66 | 5.26 | a |
|  | c1coc(c12)ccc(c2)-c3cnc(nc3)N(C4)[C@@H](C5)CN([C@H]45)C | ([Li et al., 2010b](#_ENREF_22)) | 1,300 | 5.89 | 5.55 | a |
|  | c1csc(c12)ccc(c2)-c3cnc(nc3)N(C4)[C@@H](C5)CN([C@H]45)C | ([Li et al., 2010b](#_ENREF_22)) | 560 | 6.25 | 5.75 | a |
|  | [nH]1ccc(c12)cc(cc2)-c3cnc(nc3)N(C4)[C@@H](C5)CN([C@H]45)C | ([Li et al., 2010b](#_ENREF_22)) | 4,300 | 5.37 | 5.49 | b |
|  | FC(F)(F)c([nH]1)cc(c12)cc(cc2)-c3cnc(nc3)N(C4)[C@@H](C5)CN([C@H]45)C | ([Li et al., 2010b](#_ENREF_22)) | 1,850 | 5.73 | 5.41 | b |
|  | c1c[nH]c(c12)cccc2-c3cnc(nc3)N(C4)[C@@H](C5)CN([C@H]45)C | ([Li et al., 2010b](#_ENREF_22)) | 13,000 | 4.89 | 4.87 | a |
|  | c1ccccc1-c2ccc(cn2)N(C3)[C@@H](C4)CN([C@H]34)C | ([Li et al., 2010b](#_ENREF_22)) | 10,000 | 5.00 | 5.47 | b |
|  | c1coc(c12)ccc(c2)-c3ccc(cn3)N(C4)[C@@H](C5)CN([C@H]45)C | ([Li et al., 2010b](#_ENREF_22)) | 2,700 | 5.57 | 5.14 | b |
|  | c1csc(c12)ccc(c2)-c3ccc(cn3)N(C4)[C@@H](C5)CN([C@H]45)C | ([Li et al., 2010b](#_ENREF_22)) | 550 | 6.26 | 5.90 | a |
|  | c1n[nH]c(c12)ccc(c2)-c3ccc(cn3)N(C4)[C@@H](C5)CN([C@H]45)C | ([Li et al., 2010b](#_ENREF_22)) | 46,000 | 4.34 | 4.96 | b |
|  | [nH]1ccc(c12)cc(cc2)-c3ccc(nc3)N(C4)[C@@H](C5)CN([C@H]45)C | ([Li et al., 2010b](#_ENREF_22)) | 11,000 | 4.96 | 5.38 | a |
|  | FC(F)(F)c([nH]1)cc(c12)cc(cc2)-c3ncc(cc3)N(C4)[C@@H](C5)CN([C@H]45)C | ([Li et al., 2010b](#_ENREF_22)) | 2,000 | 5.70 | 5.52 | a |
|  | c1c[nH]c(c12)cccc2-c3ccc(cn3)N(C4)[C@@H](C5)CN([C@H]45)C | ([Li et al., 2010b](#_ENREF_22)) | 32,000 | 4.49 | 4.51 | a |
|  | [nH]1ccc(c12)ccc(c2)-c3ncc(cc3)N(C4)[C@@H](C5)CN([C@H]45)C | ([Li et al., 2010b](#_ENREF_22)) | 2,900 | 5.54 | 5.47 | b |
|  | COc1cc(N)c(Cl)cc1C(=O)N[C@H]2CCN(C[C@H]2OC)CCCOc(cc3)ccc3F  **Cisapride** | ([Deacon et al., 2007](#_ENREF_9)) | 80 | 7.10 | 7.16 | a,* |
|  | c1cccc(c12)CCc3c(cccc3)N2CCCNC  **Desipramine** | ([Deacon et al., 2007](#_ENREF_9)) | 812 | 6.09 | 5.72 | a |
|  | c1ccccc1[C@H](CCNC)Oc(cc2)ccc2C(F)(F)F  **Fluoxetine** | ([Deacon et al., 2007](#_ENREF_9)) | 1,688 | 5.77 | 5.79 | b |
|  | c1cc(F)ccc1C(=O)CCCN(CC2)CCC2(O)c(cc3)ccc3Cl  **Haloperidol** | ([Deacon et al., 2007](#_ENREF_9)) | 354 | 6.45 | 6.54 | a |
|  | c1cccc(c12)Nc3c(cc(s3)C)C(=N2)N4CCN(C)CC4  **Olanzapine** | ([Deacon et al., 2007](#_ENREF_9)) | 7,300 | 5.14 | 5.34 | a |
|  | c1ccccc1CCC(=O)c2ccccc2OC[C@@H](O)CNCCC  **Propafenone** | ([Deacon et al., 2007](#_ENREF_9)) | 337 | 6.47 | 6.34 | a |
|  | CN1CCCC[C@@H]1CCN2c(cccc3)c3Sc(c24)ccc(c4)SC  **Thioridazine** | ([Deacon et al., 2007](#_ENREF_9)) | 737 | 6.13 | 6.10 | b |
|  | COc(c1)c(OC)ccc1CCN(C)CCC[C@@](C#N)(C(C)C)c(cc2)cc(OC)c2OC  **Verapamil** | ([Deacon et al., 2007](#_ENREF_9)) | 1,674 | 5.78 | 5.78 | b |
|  | c1cccc(c12)N(CCCN(C)C)c3c(CC2)cccc3  **Imipramine** | ([Murphy et al., 2006](#_ENREF_27)) | 4,600 | 5.34 | 5.91 | b |
|  | C=C[C@@H]1C[C@@H](CC2)[C@@H](C[C@H]12)[C@@H](O)c3ccc(N)c(c34)ccc(c4)OC  **Quinidine** | ([Murphy et al., 2006](#_ENREF_27)) | 6,000 | 5.22 | 5.21 | a |
|  | c1cc(F)cc(c12)onc2C(CC3)CCN3c(c(n4)C)c(=O)n(c45)CCCC5  **Risperidone** | ([Murphy et al., 2006](#_ENREF_27)) | 1,800 | 5.74 | 5.68 | a |
|  | c1ccccc1C(O)(c2ccccc2)C(CC3)CCN3CCC[C@H](O)c(cc4)ccc4C(C)(C)C  **Terfenadine** | ([Murphy et al., 2006](#_ENREF_27)) | 130 | 6.89 | 6.48 | b,* |
|  | c1cccc(c12)C(=CCCN(C)C)c3c(CC2)cccc3  **Amitriptyline** | ([Murphy et al., 2006](#_ENREF_27)) | 7,200 | 5.14 | 5.44 | a |
|  | c1cccc(c12)n(C)c(n2)N(C)CCN(C)S(=O)(=O)c(cc3)ccc3NS(=O)(=O)C  **Way123398** | ([Finlayson et al., 2001](#_ENREF_13)) | 687 | 6.16 | 6.06 | a |
|  | Clc1cnc(cc1)NC(=O)c2c(cccc2)NC(=O)c(cc3)ccc3/C(=N/[H])N(C)C | ([Pinto et al., 2010](#_ENREF_31)) | 100 | 7.00 | 6.48 | b,* |
|  | Clc1cnc(cc1)NC(=O)c2c(ccc(Cl)c2)NC(=O)c(cc3)ccc3/C(=N/[H])N(C)C | ([Pinto et al., 2010](#_ENREF_31)) | 100 | 7.00 | 6.91 | a,* |
|  | Clc1cnc(cc1)NC(=O)c2c(ccc(Cl)c2)NC(=O)c(cc3)ccc3/C(=N/[H])N4CCCCC4 | ([Pinto et al., 2010](#_ENREF_31)) | 200 | 6.70 | 6.36 | b |
|  | o1c(C)ccc1-c(n2)nc(-n3c(C)cc(n3)C)cc2NC(=O)COc4ccccc4CCN5CCOCC5 | ([Zhang et al., 2008](#_ENREF_34)) | 180 | 6.74 | 6.76 | b |
|  | COc1ccccc1Oc(c2)cccc2CN3CCC(CC3)NC(=O)C4(CCNCC4)c5ccccc5 | ([Ghosh et al., 2006](#_ENREF_17)) | 800 | 6.10 | 6.09 | b |
|  | CNCc1cnc(C)cc1Oc2ccccc2Oc3ccccc3 | ([Fish et al., 2008](#_ENREF_15)) | 5,800 | 5.24 | 5.45 | a |
|  | c1cc(Cl)cc(c12)CC(CCN(C)C)=C2Cc3nccnc3 | ([Li et al., 2010a](#_ENREF_21)) | 1,400 | 5.85 | 6.01 | b |
|  | c1cc(F)cc(c12)CC(CCN(C)C)=C2Cc3nccnc3 | ([Li et al., 2010a](#_ENREF_21)) | 950 | 6.02 | 5.95 | b |
|  | c1cc(Cl)cc(c12)CC(CCN(C)C)=C2Cc3nccnc3OC | ([Li et al., 2010a](#_ENREF_21)) | 1,300 | 5.89 | 5.86 | b |
|  | c1cc(Cl)cc(c12)CC(CCN(C)C)=C2Cc3nnccc3 | ([Li et al., 2010a](#_ENREF_21)) | 380 | 6.42 | 6.07 | b |
|  | c1cc(C)cc(c12)CC(CCN(C)C)=C2[C@@H](C)c3nccnc3 | ([Li et al., 2010a](#_ENREF_21)) | 5,100 | 5.29 | 5.48 | a |
|  | c1cc(F)cc(c12)CC(CCN(C)C)=C2[C@@H](C)c3nccs3 | ([Li et al., 2010a](#_ENREF_21)) | 1,200 | 5.92 | 5.69 | b |
|  | c1cc(Cl)ccc1-c2nc(no2)COc(c3)ccc(c34)cccc4N(CC5)CCN5C | ([Lowe et al., 2007](#_ENREF_24)) | 1,280 | 5.89 | 5.79 | b |
|  | c1cc(Cl)ccc1-c2nc(no2)COc(cc3)cc(c34)[C@H](CC4)N(CC5)CCN5C(C)C | ([Lowe et al., 2007](#_ENREF_24)) | 98 | 7.01 | 7.04 | a,* |
|  | c1cc(Cl)ccc1-c2nc(no2)COc(c3)ccc(c34)CC[C@H]4N5CCN(CC5)[C@@H]6C[C@@H](C)[C@@H](C6)C | ([Lowe et al., 2007](#_ENREF_24)) | 261 | 6.58 | 6.60 | a |
|  | c1cc(Cl)ccc1-c2nc(no2)COc(cc3)cc(c34)[C@H](CC4)N(CC5)CCN5C6(CO)CCCC6 | ([Lowe et al., 2007](#_ENREF_24)) | 205 | 6.69 | 6.57 | b |
|  | c1cccc(F)c1-c2nc(no2)COc(c3)ccc(c34)CC[C@H]4N5CCN(CC5)C6CCCC6 | ([Lowe et al., 2007](#_ENREF_24)) | 693 | 6.16 | 6.17 | a |
|  | Fc1ccccc1-c2nc(no2)COc(cc3)cc(c34)[C@H](CC4)N(CC5)CCN5C6(CO)CCCC6 | ([Lowe et al., 2007](#_ENREF_24)) | 709 | 6.15 | 6.19 | b |
|  | c1cc(Cl)ccc1-c2nc(no2)COc(c3)ccc(c34)CC[C@H]4CCCNC5(CO)CCCC5 | ([Lowe et al., 2007](#_ENREF_24)) | 525 | 6.28 | 6.17 | a |
|  | c1cc(Cl)ccc1-c2nc(no2)COc(cc3)cc(c34)[C@@H](CCC4)CCCN5CCC[C@H](C5)CO | ([Lowe et al., 2007](#_ENREF_24)) | 271 | 6.57 | 6.47 | a |
|  | c1cccc(F)c1-c2nc(no2)COc(cc3)cc(c34)[C@@H](CCC4)CCCN5CCC[C@H](C5)CO | ([Lowe et al., 2007](#_ENREF_24)) | 1,300 | 5.89 | 5.98 | a |
|  | Fc1ccccc1-c2ccc(nc2)COc(cc3)cc(c34)[C@@H](CCC4)CCCN5CCC[C@H](C5)CO | ([Lowe et al., 2007](#_ENREF_24)) | 410 | 6.39 | 6.53 | a |
|  | c1cccc(F)c1-c2nc(no2)COc3cc(ccc3)CN(CC)[C@@H]4CN(CC4)[C@@H]5C[C@@H](C)[C@@H](C5)C | ([Lowe et al., 2007](#_ENREF_24)) | 222 | 6.65 | 6.67 | b |
|  | n1c(C)ccc(c12)cc(cc2)CCN3[C@H](C)CCC3 | ([Liu et al., 2010](#_ENREF_23)) | 54,000 | 4.27 | 4.99 | a |

**References**

Angus, D., Bingham, M., Buchanan, D., Dunbar, N., Gibson, L., Goodwin, R., et al. (2011). The identification, and optimisation of hERG selectivity, of a mixed NET/SERT re-uptake inhibitor for the treatment of pain. *Bioorg Med Chem Lett* 21(1)**,** 271-275. doi: 10.1016/j.bmcl.2010.11.021.

Aspiotis, R., Chen, A., Cauchon, E., Dube, D., Falgueyret, J.P., Gagne, S., et al. (2011). The discovery and synthesis of potent zwitterionic inhibitors of renin. *Bioorg Med Chem Lett* 21(8)**,** 2430-2436. doi: 10.1016/j.bmcl.2011.02.067.

Blackburn, C., LaMarche, M.J., Brown, J., Che, J.L., Cullis, C.A., Lai, S., et al. (2006). Identification and characterization of amino-piperidinequinolones and quinazolinones as MCHr1 antagonists. *Bioorg Med Chem Lett* 16(10)**,** 2621-2627. doi: 10.1016/j.bmcl.2006.02.044.

Boezio, A.A., Berry, L., Albrecht, B.K., Bauer, D., Bellon, S.F., Bode, C., et al. (2009). Discovery and optimization of potent and selective triazolopyridazine series of c-Met inhibitors. *Bioorg Med Chem Lett* 19(22)**,** 6307-6312. doi: 10.1016/j.bmcl.2009.09.096.

Bromidge, S.M., Arban, R., Bertani, B., Borriello, M., Capelli, A.M., Di-Fabio, R., et al. (2010). 5-{2-[4-(2-methyl-5-quinolinyl)-1-piperazinyl]ethyl}-2(1H)-quinolinones and 3,4-dihydro-2(1H)-quinolinones: dual-acting 5-HT1 receptor antagonists and serotonin reuptake inhibitors. Part 3. *Bioorg Med Chem Lett* 20(23)**,** 7092-7096. doi: 10.1016/j.bmcl.2010.09.085.

Bromidge, S.M., Bertani, B., Borriello, M., Bozzoli, A., Faedo, S., Gianotti, M., et al. (2009). 8-[2-(4-Aryl-1-piperazinyl)ethyl]-2H-1,4-benzoxazin-3(4H)-ones: dual-acting 5-HT1 receptor antagonists and serotonin reuptake inhibitors--part II. *Bioorg Med Chem Lett* 19(8)**,** 2338-2342. doi: 10.1016/j.bmcl.2009.02.056.

Caldwell, C.G., Chen, P., He, J., Parmee, E.R., Leiting, B., Marsilio, F., et al. (2004). Fluoropyrrolidine amides as dipeptidyl peptidase IV inhibitors. *Bioorg Med Chem Lett* 14(5)**,** 1265-1268. doi: 10.1016/j.bmcl.2003.12.040.

Coon, T., Moree, W.J., Li, B., Yu, J., Zamani-Kord, S., Malany, S., et al. (2009). Brain-penetrating 2-aminobenzimidazole H(1)-antihistamines for the treatment of insomnia. *Bioorg Med Chem Lett* 19(15)**,** 4380-4384. doi: 10.1016/j.bmcl.2009.05.086.

Deacon, M., Singleton, D., Szalkai, N., Pasieczny, R., Peacock, C., Price, D., et al. (2007). Early evaluation of compound QT prolongation effects: a predictive 384-well fluorescence polarization binding assay for measuring hERG blockade. *J Pharmacol Toxicol Methods* 55(3)**,** 238-247. doi: 10.1016/j.vascn.2006.09.003.

Diaz, G.J., Daniell, K., Leitza, S.T., Martin, R.L., Su, Z., McDermott, J.S., et al. (2004). The [3H]dofetilide binding assay is a predictive screening tool for hERG blockade and proarrhythmia: Comparison of intact cell and membrane preparations and effects of altering [K+]o. *J Pharmacol Toxicol Methods* 50(3)**,** 187-199. doi: 10.1016/j.vascn.2004.04.001.

Dyck, B., Markison, S., Zhao, L., Tamiya, J., Grey, J., Rowbottom, M.W., et al. (2006). A thienopyridazinone-based melanin-concentrating hormone receptor 1 antagonist with potent in vivo anorectic properties. *J Med Chem* 49(13)**,** 3753-3756. doi: 10.1021/jm051263c.

Favor, D.A., Powers, J.J., White, A.D., Fitzgerald, L.W., Groppi, V., and Serpa, K.A. (2010). 6-Alkoxyisoindolin-1-one based dopamine D2 partial agonists as potential antipsychotics. *Bioorg Med Chem Lett* 20(19)**,** 5666-5669. doi: 10.1016/j.bmcl.2010.08.023.

Finlayson, K., Turnbull, L., January, C.T., Sharkey, J., and Kelly, J.S. (2001). [3H]dofetilide binding to HERG transfected membranes: a potential high throughput preclinical screen. *Eur J Pharmacol* 430(1)**,** 147-148.

Fish, L.R., Gilligan, M.T., Humphries, A.C., Ivarsson, M., Ladduwahetty, T., Merchant, K.J., et al. (2005). 4-Fluorosulfonylpiperidines: selective 5-HT2A ligands for the treatment of insomnia. *Bioorg Med Chem Lett* 15(16)**,** 3665-3669. doi: 10.1016/j.bmcl.2005.05.104.

Fish, P.V., Ryckmans, T., Stobie, A., and Wakenhut, F. (2008). [4-(Phenoxy)pyridin-3-yl]methylamines: a new class of selective noradrenaline reuptake inhibitors. *Bioorg Med Chem Lett* 18(6)**,** 1795-1798. doi: 10.1016/j.bmcl.2008.02.036.

Fletcher, S.R., Burkamp, F., Blurton, P., Cheng, S.K., Clarkson, R., O'Connor, D., et al. (2002). 4-(Phenylsulfonyl)piperidines: novel, selective, and bioavailable 5-HT(2A) receptor antagonists. *J Med Chem* 45(2)**,** 492-503.

Ghosh, S., Elder, A., Guo, J., Mani, U., Patane, M., Carson, K., et al. (2006). Design, synthesis, and progress toward optimization of potent small molecule antagonists of CC chemokine receptor 8 (CCR8). *J Med Chem* 49(9)**,** 2669-2672. doi: 10.1021/jm050965z.

Hayashi, S., Nakata, E., Morita, A., Mizuno, K., Yamamura, K., Kato, A., et al. (2010). Discovery of {1-[4-(2-{hexahydropyrrolo[3,4-c]pyrrol-2(1H)-yl}-1H-benzimidazol-1-yl)piperidin- 1-yl]cyclooctyl}methanol, systemically potent novel non-peptide agonist of nociceptin/orphanin FQ receptor as analgesic for the treatment of neuropathic pain: design, synthesis, and structure-activity relationships. *Bioorg Med Chem* 18(21)**,** 7675-7699. doi: 10.1016/j.bmc.2010.07.034.

Ladduwahetty, T., Boase, A.L., Mitchinson, A., Quin, C., Patel, S., Chapman, K., et al. (2006). A new class of selective, non-basic 5-HT2A receptor antagonists. *Bioorg Med Chem Lett* 16(12)**,** 3201-3204. doi: 10.1016/j.bmcl.2006.03.050.

Levoin, N., Labeeuw, O., Calmels, T., Poupardin-Olivier, O., Berrebi-Bertrand, I., Lecomte, J.M., et al. (2011). Novel and highly potent histamine H3 receptor ligands. Part 1: withdrawing of hERG activity. *Bioorg Med Chem Lett* 21(18)**,** 5378-5383. doi: 10.1016/j.bmcl.2011.07.006.

Li, B.F., Moree, W.J., Yu, J., Coon, T., Zamani-Kord, S., Malany, S., et al. (2010a). Selectivity profiling of novel indene H(1)-antihistamines for the treatment of insomnia. *Bioorg Med Chem Lett* 20(8)**,** 2629-2633. doi: 10.1016/j.bmcl.2010.02.055.

Li, T., Bunnelle, W.H., Ryther, K.B., Anderson, D.J., Malysz, J., Helfrich, R., et al. (2010b). Syntheses and structure-activity relationship (SAR) studies of 2,5-diazabicyclo[2.2.1]heptanes as novel alpha7 neuronal nicotinic receptor (NNR) ligands. *Bioorg Med Chem Lett* 20(12)**,** 3636-3639. doi: 10.1016/j.bmcl.2010.04.105.

Liu, H., Altenbach, R.J., Diaz, G.J., Manelli, A.M., Martin, R.L., Miller, T.R., et al. (2010). In vitro studies on a class of quinoline containing histamine H3 antagonists. *Bioorg Med Chem Lett* 20(11)**,** 3295-3300. doi: 10.1016/j.bmcl.2010.04.045.

Lowe, J., Drozda, S., Qian, W., Peakman, M.C., Liu, J., Gibbs, J., et al. (2007). A novel, non-substrate-based series of glycine type 1 transporter inhibitors derived from high-throughput screening. *Bioorg Med Chem Lett* 17(6)**,** 1675-1678. doi: 10.1016/j.bmcl.2006.12.109.

Montgomery, J.I., Toogood, P.L., Hutchings, K.M., Liu, J., Narasimhan, L., Braden, T., et al. (2009). Discovery and SAR of benzyl phenyl ethers as inhibitors of bacterial phenylalanyl-tRNA synthetase. *Bioorg Med Chem Lett* 19(3)**,** 665-669. doi: 10.1016/j.bmcl.2008.12.054.

Moree, W.J., Jovic, F., Coon, T., Yu, J., Li, B.F., Tucci, F.C., et al. (2010). Novel benzothiophene H1-antihistamines for the treatment of insomnia. *Bioorg Med Chem Lett* 20(7)**,** 2316-2320. doi: 10.1016/j.bmcl.2010.01.134.

Murphy, S.M., Palmer, M., Poole, M.F., Padegimas, L., Hunady, K., Danzig, J., et al. (2006). Evaluation of functional and binding assays in cells expressing either recombinant or endogenous hERG channel. *J Pharmacol Toxicol Methods* 54(1)**,** 42-55. doi: 10.1016/j.vascn.2005.10.003.

Owen, D.R., Rodriguez-Lens, M., Corless, M.D., Gaulier, S.M., Horne, V.A., Kinloch, R.A., et al. (2009). 2,4-Diaminopyridine delta-opioid receptor agonists and their associated hERG pharmacology. *Bioorg Med Chem Lett* 19(6)**,** 1702-1706. doi: 10.1016/j.bmcl.2009.01.106.

Palin, R., Clark, J.K., Evans, L., Feilden, H., Fletcher, D., Hamilton, N.M., et al. (2009). Rapid access towards follow-up NOP receptor agonists using a knowledge based approach. *Bioorg Med Chem Lett* 19(22)**,** 6441-6446. doi: 10.1016/j.bmcl.2009.09.028.

Patel, S.D., Habeski, W.M., Cheng, A.C., de la Cruz, E., Loh, C., and Kablaoui, N.M. (2009). Quinazolin-4-piperidin-4-methyl sulfamide PC-1 inhibitors: alleviating hERG interactions through structure based design. *Bioorg Med Chem Lett* 19(12)**,** 3339-3343. doi: 10.1016/j.bmcl.2009.04.006.

Pinto, D.J., Smallheer, J.M., Cheney, D.L., Knabb, R.M., and Wexler, R.R. (2010). Factor Xa inhibitors: next-generation antithrombotic agents. *J Med Chem* 53(17)**,** 6243-6274. doi: 10.1021/jm100146h.

Ratcliffe, P., Adam, J.M., Baker, J., Bursi, R., Campbell, R., Clark, J.K., et al. (2011). Design, synthesis and structure-activity relationships of (indo-3-yl) heterocyclic derivatives as agonists of the CB1 receptor. Discovery of a clinical candidate. *Bioorg Med Chem Lett* 21(8)**,** 2541-2546. doi: 10.1016/j.bmcl.2011.02.023.

Zhang, P., Huang, W., Wang, L., Bao, L., Jia, Z.J., Bauer, S.M., et al. (2009). Discovery of betrixaban (PRT054021), N-(5-chloropyridin-2-yl)-2-(4-(N,N-dimethylcarbamimidoyl)benzamido)-5-methoxybenz amide, a highly potent, selective, and orally efficacious factor Xa inhibitor. *Bioorg Med Chem Lett* 19(8)**,** 2179-2185. doi: 10.1016/j.bmcl.2009.02.111.

Zhang, X., Rueter, J.K., Chen, Y., Moorjani, M., Lanier, M.C., Lin, E., et al. (2008). Synthesis of N-pyrimidinyl-2-phenoxyacetamides as adenosine A2A receptor antagonists. *Bioorg Med Chem Lett* 18(6)**,** 1778-1783. doi: 10.1016/j.bmcl.2008.02.032.

Zhu, B.Y., Jia, Z.J., Zhang, P., Su, T., Huang, W., Goldman, E., et al. (2006). Inhibitory effect of carboxylic acid group on hERG binding. *Bioorg Med Chem Lett* 16(21)**,** 5507-5512. doi: 10.1016/j.bmcl.2006.08.039.
