# Supplementary material for: Computational Tool for Fast in silico Evaluation of hERG K+ Channel Affinity
Source: Front Chem. 2017 Feb 23;5:7. doi: 10.3389/fchem.2017.00007 (PMC5408157; doi:10.3389/fchem.2017.00007)
Supplement: Supplementary file 2 [file Table2.DOCX]

**Table S2.** Experimental (Observed column) and predicted (Predicted column) activity p*Ki* (M) for compounds included in the external test set. (Note: * compounds used for the decoys generation).

| **Compounds** | | | **Observed *Ki* (nM)** | **Observed p*Ki*** | **Predicted p*Ki*** | **Note** |
| --- | --- | --- | --- | --- | --- | --- |
|  | c1cccc(c12)snc2N(CC3)CCN3CCc(c4)ccc(c45)CC[C@@H]5NC(=O)C(F)(F)F | ([Graham et al., 2008](#_ENREF_14)) | 101 | 6.99 | 6.67 | * |
|  | c1cccc(c12)c(ns2)N(CC3)CCN3CCc(c4)ccc(c45)[C@H](CC5)NC(=O)C(F)(F)F | ([Graham et al., 2008](#_ENREF_14)) | 143 | 6.84 | 6.81 | * |
|  | c1cccc(c12)snc2N(CC3)CCN3CCc(c4)ccc(c45)CC[C@@H]5NC(=O)N | ([Graham et al., 2008](#_ENREF_14)) | 616 | 6.21 | 6.17 |  |
|  | c1cccc(c12)snc2N(CC3)CCN3CCc(c4)ccc(c45)CC[C@@H]5NC(=O)C | ([Graham et al., 2008](#_ENREF_14)) | 885 | 6.05 | 6.36 |  |
|  | c1cccc(c12)snc2N(CC3)CCN3CCc(c4)ccc(c45)CC[C@@H]5NC(=O)CC | ([Graham et al., 2008](#_ENREF_14)) | 164 | 6.78 | 6.84 |  |
|  | c1cccc(c12)snc2N(CC3)CCN3CCc(c4)ccc(c45)CC[C@@H]5NC(=O)CCC | ([Graham et al., 2008](#_ENREF_14)) | 196 | 6.71 | 6.59 |  |
|  | c1cccc(c12)snc2N(CC3)CCN3CCc(c4)ccc(c45)CC[C@@H]5NC(=O)C6CC6 | ([Graham et al., 2008](#_ENREF_14)) | 35 | 7.46 | 6.87 | * |
|  | c1cccc(c12)snc2N(CC3)CCN3CCc(c4)ccc(c45)CC[C@@H]5NC(=O)C(C)C | ([Graham et al., 2008](#_ENREF_14)) | 278 | 6.56 | 6.34 |  |
|  | c1cccc(c12)snc2N(CC3)CCN3CCc(c4)ccc(c45)CC[C@@H]5NC(=O)c6ccccc6 | ([Graham et al., 2008](#_ENREF_14)) | 272 | 6.56 | 6.65 |  |
|  | c1cccc(c12)snc2N(CC3)CCN3CCc(c4)ccc(c45)CC[C@@H]5NC(=O)c(cc6)ccc6C | ([Graham et al., 2008](#_ENREF_14)) | 194 | 6.71 | 6.82 |  |
|  | c1cccc(c12)snc2N(CC3)CCN3CCc(c4)ccc(c45)CC[C@@H]5NC(=O)c(cc6)ccc6Cl | ([Graham et al., 2008](#_ENREF_14)) | 166 | 6.78 | 6.70 |  |
|  | c1cccc(c12)snc2N(CC3)CCN3CCc(c4)ccc(c45)CC[C@@H]5NC(=O)c(cc6)ccc6F | ([Graham et al., 2008](#_ENREF_14)) | 78 | 7.11 | 6.92 | * |
|  | c1cccc(c12)snc2N(CC3)CCN3CCc(c4)ccc(c45)CC[C@@H]5NC(=O)c6cnccc6 | ([Graham et al., 2008](#_ENREF_14)) | 358 | 6.45 | 6.58 |  |
|  | c1cccc(c12)snc2N(CC3)CCN3CCc(c4)ccc(c45)CC[C@@H]5NC(=O)Cc6ccccc6 | ([Graham et al., 2008](#_ENREF_14)) | 201 | 6.69 | 6.42 |  |
|  | c1cccc(c12)snc2N(CC3)CCN3CCc(c4)ccc(c45)CC[C@@H]5NC(=O)CCc6ccccc6 | ([Graham et al., 2008](#_ENREF_14)) | 511 | 6.29 | 6.15 |  |
|  | c1cccc(c12)snc2N(CC3)CCN3CCc(c4)ccc(c45)CC[C@@H]5NC(=O)c6ccco6 | ([Graham et al., 2008](#_ENREF_14)) | 35 | 7.46 | 7.18 | * |
|  | c1cccc(c12)snc2N(CC3)CCN3CCc(c4)ccc(c45)CC[C@@H]5NC(=O)c6ccno6 | ([Graham et al., 2008](#_ENREF_14)) | 150 | 6.82 | 6.90 | * |
|  | c1cccc(c12)c(ns2)N(CC3)CCN3CCc(c4)ccc(c45)[C@H](CC5)NC(=O)C | ([Graham et al., 2008](#_ENREF_14)) | 2,202 | 5.66 | 5.78 |  |
|  | c1cccc(c12)c(ns2)N(CC3)CCN3CCc(c4)ccc(c45)[C@H](CC5)NC(=O)CC | ([Graham et al., 2008](#_ENREF_14)) | 184 | 6.73 | 6.78 |  |
|  | c1cccc(c12)c(ns2)N(CC3)CCN3CCc(c4)ccc(c45)[C@H](CC5)NC(=O)CCC | ([Graham et al., 2008](#_ENREF_14)) | 269 | 6.57 | 6.59 |  |
|  | c1cccc(c12)c(ns2)N(CC3)CCN3CCc(c4)ccc(c45)[C@H](CC5)NC(=O)C6CC6 | ([Graham et al., 2008](#_ENREF_14)) | 137 | 6.86 | 6.91 | * |
|  | c1cccc(c12)c(ns2)N(CC3)CCN3CCc(c4)ccc(c45)[C@H](CC5)NC(=O)C(C)C | ([Graham et al., 2008](#_ENREF_14)) | 219 | 6.66 | 6.74 |  |
|  | c1cccc(c12)c(ns2)N(CC3)CCN3CCc(c4)ccc(c45)[C@H](CC5)NC(=O)c(cc6)ccc6F | ([Graham et al., 2008](#_ENREF_14)) | 135 | 6.87 | 7.05 | * |
|  | c1cccc(c12)c(ns2)N(CC3)CCN3CCc(c4)ccc(c45)[C@H](CC5)NC(=O)c6cccnc6 | ([Graham et al., 2008](#_ENREF_14)) | 202 | 7.15 | 6.79 |  |
|  | Fc1ccc(cc1)Cn(c(c23)cccc2)c(n3)N(CC4)CCC4n5ccc(n5)C | ([Lavrador-Erb et al., 2010](#_ENREF_17)) | 3,849 | 5.41 | 5.67 |  |
|  | C1CCN(C)C[C@@H]1c(n2)n(c(c23)cccc3)CCOc4ccccc4 | ([Lavrador-Erb et al., 2010](#_ENREF_17)) | 5,291 | 5.28 | 5.90 |  |
|  | c1cccc(c12)n(CCOCC)c(n2)[C@H]3CN(C)CCC3 | ([Lavrador-Erb et al., 2010](#_ENREF_17)) | 1,262 | 5.90 | 5.66 |  |
|  | C1CCO[C@@H]1Cn(c(c23)cccc2)c(n3)[C@H]4CN(C)CCC4 | ([Lavrador-Erb et al., 2010](#_ENREF_17)) | 638 | 6.19 | 6.16 |  |
|  | Fc1ccc(cc1)Cn(c(c23)cccc2)c(n3)[C@H]4CN(C(C)C)CCC4 | ([Lavrador-Erb et al., 2010](#_ENREF_17)) | 1,373 | 5.86 | 5.77 |  |
|  | Fc1ccc(cc1)Cn(c(c23)cccc2)c(n3)[C@H]4CN(CCC4)C5CCCCC5 | ([Lavrador-Erb et al., 2010](#_ENREF_17)) | 926 | 6.03 | 5.83 |  |
|  | COc1ccc(cc1)Cn(c(c23)cccc2)c(n3)[C@H]4CN(C(C)C)CCC4 | ([Lavrador-Erb et al., 2010](#_ENREF_17)) | 2,649 | 5.58 | 5.95 |  |
|  | COc1ccc(cc1)Cn(c(c23)cccc2)c(n3)[C@H]4CN(CCC4)C5CCCCC5 | ([Lavrador-Erb et al., 2010](#_ENREF_17)) | 1,840 | 5.73 | 6.21 |  |
|  | s1cc(C)nc1Cn(c(c23)cccc2)c(n3)[C@H]4CN(CCC4)C5CCCCC5 | ([Lavrador-Erb et al., 2010](#_ENREF_17)) | 1,183 | 5.93 | 6.48 |  |
|  | Fc1ccc(cc1)Cn(c(c23)cccc2)c(n3)[C@H]4CN(C)CCC4 | ([Lavrador-Erb et al., 2010](#_ENREF_17)) | 3,860 | 5.41 | 5.55 |  |
|  | Fc1ccc(cc1)Cn(c(c23)cccc2)c(n3)[C@@H]4CN(C)CCC4 | ([Lavrador-Erb et al., 2010](#_ENREF_17)) | 6,515 | 5.19 | 5.55 |  |
|  | c1cccnc1Cn(c(c23)cccc2)c(n3)[C@H]4CN(C)CCC4 | ([Lavrador-Erb et al., 2010](#_ENREF_17)) | 10,336 | 4.99 | 4.76 |  |
|  | c1ccc(C(F)(F)F)cc1-c(c2)nc(C#N)nc2CCCN3CCCCC3 | ([Rankovic et al., 2010](#_ENREF_23)) | 160 | 6.79 | 7.04 |  |
|  | CN(C)CCN(C)C(=O)c(c1)cc(C(F)(F)F)cc1-c(c2)nc(C#N)nc2CCC | ([Rankovic et al., 2010](#_ENREF_23)) | 710 | 6.15 | 6.33 |  |
|  | CCCc1nc(C#N)nc(c1)-c2cc(C(F)(F)F)cc(c2)C(=O)N(CCN(C)C)Cc3ccccc3 | ([Rankovic et al., 2010](#_ENREF_23)) | 120 | 6.92 | 6.35 | * |
|  | CCCc1nc(C#N)nc(c1)-c2cc(C(F)(F)F)cc(c2)C(=O)N(C)Cc3ccccc3 | ([Rankovic et al., 2010](#_ENREF_23)) | 370 | 6.43 | 6.09 |  |
|  | c1ccc(C(F)(F)F)cc1-c(c2)nc(C#N)nc2CCCO | ([Rankovic et al., 2010](#_ENREF_23)) | 31,000 | 4.51 | 4.84 |  |
|  | c1ccc(C(F)(F)F)cc1-c(c2)nc(C#N)nc2CCC(=O)NC(CC)CC | ([Rankovic et al., 2010](#_ENREF_23)) | 3,160 | 5.50 | 5.29 |  |
|  | c1ccc(C(F)(F)F)cc1-c(c2)nc(C#N)nc2CCCNC(CC)CC | ([Rankovic et al., 2010](#_ENREF_23)) | 320 | 6.49 | 6.78 |  |
|  | c1ccc(C(F)(F)F)cc1-c(c2)nc(C#N)nc2CCCNC | ([Rankovic et al., 2010](#_ENREF_23)) | 630 | 6.20 | 6.43 |  |
|  | c1ccc(C(F)(F)F)cc1-c(c2)nc(C#N)nc2CCCNCC(F)(F)F | ([Rankovic et al., 2010](#_ENREF_23)) | 500 | 6.30 | 6.41 |  |
|  | c1ccc(C(F)(F)F)cc1-c(c2)nc(C#N)nc2CCCN(CC3)CCN3C | ([Rankovic et al., 2010](#_ENREF_23)) | 1,000 | 6.00 | 6.00 |  |
|  | c1ccc(C(F)(F)F)cc1-c(c2)nc(C#N)nc2CCCN3CCOCC3 | ([Rankovic et al., 2010](#_ENREF_23)) | 320 | 6.49 | 6.45 |  |
|  | c1ccc(C(F)(F)F)cc1-c(c2)nc(C#N)nc2CCCNCCOC | ([Rankovic et al., 2010](#_ENREF_23)) | 560 | 6.25 | 6.21 |  |
|  | c1ccc(C(F)(F)F)cc1-c(c2)nc(C#N)nc2CCCN(CC3)CCC3O | ([Rankovic et al., 2010](#_ENREF_23)) | 1,120 | 5.95 | 5.97 |  |
|  | c1ccc(C(F)(F)F)cc1-c(c2)nc(C#N)nc2CCCNc3ncccc3 | ([Rankovic et al., 2010](#_ENREF_23)) | 400 | 6.39 | 6.12 |  |
|  | c1ccc(C(F)(F)F)cc1-c(c2)nc(C#N)nc2CCCNCC(=O)N | ([Rankovic et al., 2010](#_ENREF_23)) | 3,980 | 5.40 | 5.47 |  |
|  | c1ccc(C(F)(F)F)cc1-c(c2)nc(C#N)nc2CCCNC(C)(C)C(=O)N | ([Rankovic et al., 2010](#_ENREF_23)) | 3,160 | 5.50 | 5.46 |  |
|  | c1ccc(C(F)(F)F)cc1-c(c2)nc(C#N)nc2CCCNC3(CC3)C(=O)N | ([Rankovic et al., 2010](#_ENREF_23)) | 1,580 | 5.80 | 5.75 |  |
|  | c1ccc(C(F)(F)F)cc1-c(c2)nc(C#N)nc2CCCNC3(C(=O)N)CCC3 | ([Rankovic et al., 2010](#_ENREF_23)) | 2,510 | 5.60 | 5.49 |  |
|  | c1cccc(c12)ccc(c2)CO[C@H]3CCNC3 | ([Andrews et al., 2009b](#_ENREF_3)) | 15,700 | 4.80 | 4.85 |  |
|  | c1cccc(c12)ccc(c2)CO[C@@H]3CCNC3 | ([Andrews et al., 2009b](#_ENREF_3)) | 16,400 | 4.78 | 4.62 |  |
|  | c1cc(C#N)cc(c12)ccc(c2)CO[C@H]3CCNC | ([Andrews et al., 2009b](#_ENREF_3)) | 4,500 | 5.35 | 5.51 |  |
|  | c1cc(F)cc(c12)ccc(c2)CO[C@@H]3CCNC3 | ([Andrews et al., 2009b](#_ENREF_3)) | 10,300 | 4.99 | 4.94 |  |
|  | c1cccc(c12)ccc(c2)S(=O)(=O)N(CCCC)C3CCNCC3 | ([Andrews et al., 2009b](#_ENREF_3)) | 716 | 6.14 | 6.31 |  |
|  | c1cccc(c12)ccc(c2)S(=O)(=O)N(CCCC)[C@H]3CCNC3 | ([Andrews et al., 2009b](#_ENREF_3)) | 412 | 6.38 | 6.27 |  |
|  | c1cccc(c12)cc(cc2)OCC3CCNCC3 | ([Andrews et al., 2009b](#_ENREF_3)) | 3,800 | 5.42 | 5.66 |  |
|  | c1c(C)ccc(c12)ccc(c2)OCC3CCNCC3 | ([Andrews et al., 2009b](#_ENREF_3)) | 3,500 | 5.46 | 5.61 |  |
|  | c1cc(F)cc(c12)ccc(c2)CCN3CCNCC3 | ([Andrews et al., 2009b](#_ENREF_3)) | 8,500 | 5.07 | 5.06 |  |
|  | c1cc(F)cc(c12)ccc(c2C)CCN3CCNCC3 | ([Andrews et al., 2009b](#_ENREF_3)) | 10,100 | 5.00 | 5.25 |  |
|  | c1cccc(c12)ccc(c2)C(=O)N(CCC)C3CCNCC3 | ([Andrews et al., 2009a](#_ENREF_2)) | 12,700 | 4.90 | 4.92 |  |
|  | c1cccc(c12)ccc(c2)C(=O)N(CC(C)C)[C@@H]3CCNC3 | ([Andrews et al., 2009a](#_ENREF_2)) | 7,000 | 5.15 | 5.19 |  |
|  | c1cccc(c12)ccc(c2)CN(S(=O)(=O)C)[C@@H]3CCNC3 | ([Andrews et al., 2009a](#_ENREF_2)) | 4,200 | 5.38 | 5.31 |  |
|  | c1cc(F)cc(c12)ccc(c2C)CN(S(=O)(=O)C)[C@@H]3CCNC3 | ([Andrews et al., 2009a](#_ENREF_2)) | 1,600 | 5.79 | 5.50 |  |
|  | c1cccc(c12)ccc(c2)CN(S(=O)(=O)C)C3CCNCC3 | ([Andrews et al., 2009a](#_ENREF_2)) | 3,200 | 5.49 | 5.23 |  |
|  | c1cccc(c12)ccc(c2)CN(S(=O)(=O)C)[C@H]3CNCC3 | ([Andrews et al., 2009a](#_ENREF_2)) | 11,000 | 4.96 | 5.08 |  |
|  | c1cccc(c12)ccc(c2)CN(C(=O)CC)C3CCNCC3 | ([Andrews et al., 2009a](#_ENREF_2)) | 12,000 | 4.92 | 5.16 |  |
|  | c1cccc(c12)ccc(c2)CN(C(=O)C)[C@H]3CCNC3 | ([Andrews et al., 2009a](#_ENREF_2)) | 8,000 | 5.09 | 5.32 |  |
|  | c1ccncc1-c2ccccc2O[C@H](C34)CC(C4)CNC3 | ([Lowe et al., 2010](#_ENREF_19)) | 2,160 | 5.66 | 5.47 |  |
|  | c1c(O)cncc1-c2ccccc2O[C@H]([C@H]34)C[C@H](C4)CNC3 | ([Lowe et al., 2010](#_ENREF_19)) | 1,580 | 5.80 | 5.94 |  |
|  | Fc1ncc(cc1)-c2ccccc2O[C@H]([C@H]34)C[C@H](C4)CNC3 | ([Lowe et al., 2010](#_ENREF_19)) | 1,780 | 5.75 | 5.98 |  |
|  | c1cc(F)ccc1S(=O)(=O)C2CCN(CC2)CCc(cc3)c(F)cc3F | ([Ladduwahetty et al., 2010](#_ENREF_15)) | 710 | 6.15 | 6.04 |  |
|  | c1cc(F)ccc1S(=O)(=O)C2(F)CCN(CC2)CCc(cc3)c(F)cc3F | ([Ladduwahetty et al., 2010](#_ENREF_15)) | 5,561 | 5.25 | 5.72 |  |
|  | c1ccccc1S(=O)(=O)c(cc2)ccc2/C=C/c(cc3)c(F)cc3F | ([Ladduwahetty et al., 2010](#_ENREF_15)) | 4,342 | 5.36 | 5.51 |  |
|  | c1cc(C(=O)N)ccc1S(=O)(=O)c(cc2)ccc2/C=C/c(cc3)c(F)cc3F | ([Ladduwahetty et al., 2010](#_ENREF_15)) | 7,919 | 5.10 | 5.44 |  |
|  | c1cccc(CO)c1S(=O)(=O)c(cc2)ccc2/C=C/c(cc3)ccc3F | ([Ladduwahetty et al., 2010](#_ENREF_15)) | 5,953 | 5.22 | 5.52 |  |
|  | c1cccc([C@@H](O)C)c1S(=O)(=O)c(cc2)ccc2/C=C/c(cc3)ccc3F | ([Ladduwahetty et al., 2010](#_ENREF_15)) | 4,000 | 5.40 | 5.69 |  |
|  | c1cccc([C@H](C)O)c1S(=O)(=O)c(cc2)ccc2/C=C/c(cc3)ccc3F | ([Ladduwahetty et al., 2010](#_ENREF_15)) | 8,252 | 5.08 | 4.83 |  |
|  | C1C[C@@H](CN)CN1c(c(F)c2)c(OC)c(c23)n(C4CC4)cc(c3=O)C(=O)O | ([Murphy et al., 2007](#_ENREF_21)) | 24,000 | 4.62 | 4.70 |  |
|  | C1C[C@@H]([C@@H](N)C)CN1c(c(F)c2)c(C)c(c23)n(C4CC4)cc(c3=O)C(=O)O | ([Murphy et al., 2007](#_ENREF_21)) | 51,000 | 4.29 | 4.71 |  |
|  | CCC1(CN)CN(C1)c(c(F)c2)c(C)c(c23)n(C4CC4)cc(c3=O)C(=O)O | ([Murphy et al., 2007](#_ENREF_21)) | 45,000 | 4.35 | 4.92 |  |
|  | c1cccc(c12)c(OC)ccc2S(=O)(=O)N(c(c34)cccc4)C[C@@H]3C(=O)N5CCNCCC5 | ([Reid et al., 2010](#_ENREF_26)) | 1,259 | 5.90 | 5.93 |  |
|  | c1cccc(c12)S(=O)(=O)c3c2ccc(c3)N(C45)CCN(CC4)CC5 | ([Schrimpf et al., 2012](#_ENREF_28)) | 470 | 6.33 | 6.05 |  |
|  | c1cccc(c12)oc3c(c2=O)cc(cc3)N(C4)C[C@@H]([C@H]45)CN(C5)C | ([Schrimpf et al., 2012](#_ENREF_28)) | 1,100 | 5.96 | 5.78 |  |
|  | c1cc(F)ccc1S(=O)(=O)C2(F)CCN(CC2)CCc(cc3)c(F)cc3F | ([Wilson et al., 2007](#_ENREF_30)) | 5,600 | 5.25 | 5.75 |  |
|  | c1cc(F)ccc1S(=O)(=O)c(nc2)ccc2CCc(cc3)ccc3F | ([Wilson et al., 2007](#_ENREF_30)) | 6,900 | 5.16 | 5.38 |  |
|  | c1cc(F)ccc1S(=O)(=O)c(cn2)ccc2CCc(cc3)ccc3F | ([Wilson et al., 2007](#_ENREF_30)) | 8,200 | 5.09 | 4.79 |  |
|  | c1cc(F)ccc1S(=O)(=O)c(nc2)ccc2/C=C/c(cc3)ccc3F | ([Wilson et al., 2007](#_ENREF_30)) | 3,300 | 5.48 | 5.22 |  |
|  | c1cc(F)ccc1S(=O)(=O)c(cn2)ccc2/C=C/c(cc3)ccc3F | ([Wilson et al., 2007](#_ENREF_30)) | 9,500 | 4.72 | 4.93 |  |
|  | c1cccc(F)c1S(=O)(=O)c(cn2)ccc2/C=C/c3ccncc3 | ([Wilson et al., 2007](#_ENREF_30)) | 4,200 | 5.38 | 5.61 |  |
|  | c1cccc(F)c1S(=O)(=O)c(cn2)ccc2/C=C/c(c(Cl)c3)ccc3F | ([Wilson et al., 2007](#_ENREF_30)) | 4,800 | 5.32 | 5.01 |  |
|  | c1cccc(F)c1S(=O)(=O)c(cn2)ccc2/C=C/c(c(c3)C)ccc3F | ([Wilson et al., 2007](#_ENREF_30)) | 5,000 | 5.30 | 5.13 |  |
|  | c1cccc(F)c1S(=O)(=O)c(cn2)ccc2/C=C/c(c(c3)C#N)ccc3F | ([Wilson et al., 2007](#_ENREF_30)) | 1,500 | 5.82 | 5.88 |  |
|  | c1cccc(F)c1S(=O)(=O)c(cn2)ccc2/C=C/c(c(c3)O)ccc3F | ([Wilson et al., 2007](#_ENREF_30)) | 2,100 | 5.68 | 5.44 |  |
|  | c1cccc(F)c1S(=O)(=O)c(cn2)ccc2/C=C/c3ccccc3O | ([Wilson et al., 2007](#_ENREF_30)) | 1,400 | 5.85 | 5.66 |  |
|  | c1cccc(F)c1S(=O)(=O)c(cn2)ccc2/C=C/c(ccc3F)c(c3)OC | ([Wilson et al., 2007](#_ENREF_30)) | 1,400 | 5.85 | 5.68 |  |
|  | c1cccc(F)c1[S@@](=O)c(cn2)ccc2/C=C/c(cc3)ccc3F | ([Wilson et al., 2007](#_ENREF_30)) | 2,900 | 5.54 | 5.57 |  |
|  | c1ccccc1[S@@](=O)c(cn2)ccc2/C=C/c(cc3)ccc3F | ([Wilson et al., 2007](#_ENREF_30)) | 3,900 | 5.41 | 5.58 |  |
|  | c1ccccc1[S@@](=O)c(cn2)ccc2/C=C/c(c(F)c3)ccc3F | ([Wilson et al., 2007](#_ENREF_30)) | 4,200 | 5.38 | 5.63 |  |
|  | c1ccccc1[S@](=O)c(cn2)ccc2/C=C/c(c(F)c3)ccc3F | ([Wilson et al., 2007](#_ENREF_30)) | 6,500 | 5.19 | 5.35 |  |
|  | N#Cc1ccc(cc1)-c(cc2)cc(c23)cc(o3)CCN4[C@H](C)CCC4 | ([Black et al., 2008](#_ENREF_5)) | 195 | 6.71 | 6.56 |  |
|  | N#Cc1cncc(c1)-c(cc2)cc(c23)cc(o3)CCN4[C@H](C)CCC4 | ([Black et al., 2008](#_ENREF_5)) | 1,122 | 5.95 | 6.32 |  |
|  | N#Cc1ccc(cc1)-c(cc2)cc(c23)ccc(c3)CCN4[C@H](C)CCC | ([Black et al., 2008](#_ENREF_5)) | 120 | 6.92 | 6.78 | * |
|  | COc1ncc(cn1)-c(cc2)cc(c23)ccc(c3)CCN4[C@H](C)CCC | ([Black et al., 2008](#_ENREF_5)) | 1,504 | 5.82 | 5.77 |  |
|  | O=c1cccnn1-c(cc2)cc(c23)ccc(c3)CCN4[C@H](C)CCC | ([Black et al., 2008](#_ENREF_5)) | 5,679 | 5.25 | 5.09 |  |
|  | C1CC[C@@H](C)N1[C@@H](C2)C[C@H]2c(cc3)ccc3-c(cc4)ccc4C#N | ([Black et al., 2008](#_ENREF_5)) | 107 | 6.97 | 6.77 | * |
|  | C1CC[C@@H](C)N1[C@@H](C2)C[C@H]2c(cc3)ccc3-c4cncnc4 | ([Black et al., 2008](#_ENREF_5)) | 2,630 | 5.58 | 5.69 |  |
|  | C1CC[C@@H](C)N1[C@@H](C2)C[C@H]2c(cc3)ccc3-c(cn4)cnc4OC | ([Black et al., 2008](#_ENREF_5)) | 1,180 | 5.93 | 5.97 |  |
|  | CC(C)(C)COc(cn1)cc2c1Oc(c3[C@]24N=C(N)OC4)ccc(c3)-c5c(F)nccc5 | ([Dineen et al., 2014](#_ENREF_10)) | 2,950 | 5.53 | 5.17 |  |
|  | CC(C)(C)COc(cc1)cc2c1Oc(c3[C@]24N=C(N)OC4)ncc(c3)-c5cncnc5 | ([Dineen et al., 2014](#_ENREF_10)) | 3,810 | 5.42 | 5.63 |  |
|  | c1ccnc(F)c1-c(c2)ccc(c2[C@]34N=C(N)OC4)Oc5c3cc(cn5)-c(cc6)ccc6C | ([Dineen et al., 2014](#_ENREF_10)) | 1,240 | 5.91 | 5.50 |  |
|  | c1ccnc(F)c1-c(c2)ccc(c2[C@]34N=C(N)OC4)Oc5c3cc(cn5)-c(cc6)ccc6C#N | ([Dineen et al., 2014](#_ENREF_10)) | 210 | 6.68 | 6.39 |  |
|  | CC(C)(C)C#Cc(cn1)cc2c1Oc(c3[C@]24N=C(N)OC4)ccc(c3)-c5c(F)nccc5 | ([Dineen et al., 2014](#_ENREF_10)) | 1,160 | 5.94 | 5.67 |  |
|  | C1C[C@H](F)CN1c(nc2)cc3c2Oc(c4[C@@]35N=C(N)OC5)ccc(c4)-c6cccnc6F | ([Chen et al., 2015](#_ENREF_9)) | 7,800 | 5.11 | 5.34 |  |
|  | n1c(C)ccc(c12)c(ccc2)N(CC3)CCN3CCc4cccc(c45)n6c(CO5)ncc6 | ([Bromidge et al., 2010](#_ENREF_7)) | 1,585 | 5.80 | 6.24 |  |
|  | n1c(C)ccc(c12)c(ccc2)N(CC3)CCN3CCc4cccc(c45)n6c(CO5)nc(c6)C | ([Bromidge et al., 2010](#_ENREF_7)) | 398 | 6.40 | 6.64 |  |
|  | n1c(C)ccc(c12)c(ccc2)N(CC3)CCN3CCc4cccc(c45)n6c(CO5)nc(C)c6C | ([Bromidge et al., 2010](#_ENREF_7)) | 398 | 6.40 | 6.62 |  |
|  | n1c(C)ccc(c12)c(ccc2)N(CC3)CCN3CCc4cccc(c45)n6c(CO5)nc(c6)C(F)(F)F | ([Bromidge et al., 2010](#_ENREF_7)) | 25 | 7.60 | 7.34 | * |
|  | n1c(C)ccc(c12)c(ccc2)N(CC3)CCN3CCc4cccc(c45)n6c(CO5)cnc6 | ([Bromidge et al., 2010](#_ENREF_7)) | 1,000 | 6.00 | 6.23 |  |
|  | n1c(C)ccc(c12)c(ccc2)N(CC3)CCN3CCc4cccc(c45)n6c(CO5)nnc6C | ([Bromidge et al., 2010](#_ENREF_7)) | 12,589 | 4.90 | 5.17 |  |
|  | n1c(C)ccc(c12)c(ccc2)N(CC3)CCN3CCc4cccc(c45)n6c(CO5)c(nn6)C | ([Bromidge et al., 2010](#_ENREF_7)) | 2,512 | 5.60 | 5.50 |  |
|  | n1c(C)ccc(c12)c(ccc2)N(CC3)CCN3CCc4cccc(c45)n6c(CO5)nnn6 | ([Bromidge et al., 2010](#_ENREF_7)) | 501 | 6.30 | 6.17 |  |
|  | n1c(C)ccc(c12)c(ccc2)N(CC3)CCN3CCc4cccc(c45)n6c(CO5)c(nc6)C(=O)OCC | ([Bromidge et al., 2010](#_ENREF_7)) | 7,943 | 5.10 | 5.43 |  |
|  | n1c(C)ccc(c12)c(ccc2)N(CC3)CCN3CCc4cccc(c45)n6c(CO5)c(nc6)C(=O)C | ([Bromidge et al., 2010](#_ENREF_7)) | 6,310 | 5.20 | 5.18 |  |
|  | n1c(C)ccc(c12)c(ccc2)N(CC3)CCN3CCc4cccc(c45)n6c(CO5)c(nc6)/C(C)=N/OC | ([Bromidge et al., 2010](#_ENREF_7)) | 2,512 | 5.60 | 5.37 |  |
|  | n1c(C)ccc(c12)c(ccc2)N(CC3)CCN3CCc4cccc(c45)n6c(CO5)c(nc6)C(=O)N(C)C | ([Bromidge et al., 2010](#_ENREF_7)) | 3,981 | 5.40 | 5.25 |  |
|  | n1c(C)ccc(c12)c(ccc2)N(CC3)CCN3CCc4cccc(c45)n6c(CO5)c(C#N)nc6 | ([Bromidge et al., 2010](#_ENREF_7)) | 1,000 | 6.00 | 6.24 |  |
|  | c1nc(C(=O)N)c(CO2)n1c(c23)cccc3CCN(CC4)CCN4c5cccc(c56)nc(C)cc6 | ([Bromidge et al., 2010](#_ENREF_7)) | 31,623 | 4.50 | 5.17 |  |
|  | CC(C)NC(=O)c(nc1)c(CO2)n1c(c23)cccc3CCN(CC4)CCN4c5cccc(c56)nc(C)cc6 | ([Bromidge et al., 2010](#_ENREF_7)) | 2,512 | 5.60 | 5.23 |  |
|  | C1CC1NC(=O)c(nc2)c(CO3)n2c(c34)cccc4CCN(CC5)CCN5c6cccc(c67)nc(C)cc7 | ([Bromidge et al., 2010](#_ENREF_7)) | 631 | 6.20 | 6.54 |  |
|  | C1CCC1NC(=O)c(nc2)c(CO3)n2c(c34)cccc4CCN(CC5)CCN5c6cccc(c67)nc(C)cc7 | ([Bromidge et al., 2010](#_ENREF_7)) | 501 | 6.30 | 6.68 |  |
|  | C1CCCC1NC(=O)c(nc2)c(CO3)n2c(c34)cccc4CCN(CC5)CCN5c6cccc(c67)nc(C)cc7 | ([Bromidge et al., 2010](#_ENREF_7)) | 200 | 6.70 | 6.48 |  |
|  | C1CC1CNC(=O)c(nc2)c(CO3)n2c(c34)cccc4CCN(CC5)CCN5c6cccc(c67)nc(C)cc7 | ([Bromidge et al., 2010](#_ENREF_7)) | 200 | 6.70 | 6.91 |  |
|  | C1CCN1C(=O)c(nc2)c(CO3)n2c(c34)cccc4CCN(CC5)CCN5c6cccc(c67)nc(C)cc7 | ([Bromidge et al., 2010](#_ENREF_7)) | 501 | 6.30 | 6.59 |  |
|  | C1CCCN1C(=O)c(nc2)c(CO3)n2c(c34)cccc4CCN(CC5)CCN5c6cccc(c67)nc(C)cc7 | ([Bromidge et al., 2010](#_ENREF_7)) | 1,259 | 5.90 | 5.74 |  |
|  | C1CCCCN1C(=O)c(nc2)c(CO3)n2c(c34)cccc4CCN(CC5)CCN5c6cccc(c67)nc(C)cc7 | ([Bromidge et al., 2010](#_ENREF_7)) | 1,000 | 6.00 | 6.18 |  |
|  | C1COCCN1C(=O)c(nc2)c(CO3)n2c(c34)cccc4CCN(CC5)CCN5c6cccc(c67)nc(C)cc7 | ([Bromidge et al., 2010](#_ENREF_7)) | 31,623 | 4.50 | 5.04 |  |
|  | C1COCCC1Cn2cc(c(c23)cccc3Cl)-c4noc(n4)CN5CCN(CC5)CC(=O)N **LBP1** | ([Adam et al., 2012](#_ENREF_1)) | 19,953 | 4.70 | 5.23 |  |
|  | Clc1c(Cl)cc(cc1)[C@@]23[C@@H](CNC2)[C@@H]3COCC | ([Micheli et al., 2010](#_ENREF_20)) | 12,589 | 4.90 | 5.18 |  |
|  | Fc1cc(F)c(cc1)[C@H]2CN(C(C)(C)C)C[C@@H]2C(=O)N(C[C@H]3C)C[C@H](C)[C@@]3(O)c(cc4)ccc4F | ([Lansdell et al., 2010](#_ENREF_16)) | 7,910 | 5.10 | 5.58 |  |
|  | Fc1cc(F)c(cc1)[C@H]2CN(C(C)(C)C)C[C@@H]2C(=O)N(C[C@H]3C)C[C@H](C)[C@@]3(O)c4c(F)cccc4F | ([Lansdell et al., 2010](#_ENREF_16)) | 5,000 | 5.30 | 5.52 |  |
|  | Fc1cc(F)c(cc1)[C@H]2CN(C(C)(C)C)C[C@@H]2C(=O)N(C[C@H]3C)C[C@H](C)[C@@]3(O)c4ncccc4 | ([Lansdell et al., 2010](#_ENREF_16)) | 5,840 | 5.23 | 5.59 |  |
|  | Fc1cc(F)c(cc1)[C@H]2CNC[C@@H]2C(=O)N(C[C@H]3C)C[C@H](C)[C@@]3(O)c4ccccc | ([Lansdell et al., 2010](#_ENREF_16)) | 6,650 | 5.18 | 5.51 |  |
|  | Fc1cc(F)c(cc1)[C@H]2CN(C3CCC3)C[C@@H]2C(=O)N(C[C@H]4C)C[C@H](C)[C@@]4(O)c5ccccc5 | ([Lansdell et al., 2010](#_ENREF_16)) | 5,870 | 5.23 | 5.57 |  |
|  | Fc1cc(F)c(cc1)[C@H]2CN(c3ccccn3)C[C@@H]2C(=O)N(C[C@H]4C)C[C@H](C)[C@@]4(O)c5ccccc5 | ([Lansdell et al., 2010](#_ENREF_16)) | 4,650 | 5.33 | 5.52 |  |
|  | Fc1cc(F)c(cc1)[C@H]2CN(c3cccnc3)C[C@@H]2C(=O)N(C[C@H]4C)C[C@H](C)[C@@]4(O)c5ccccc5 | ([Lansdell et al., 2010](#_ENREF_16)) | 5,070 | 5.29 | 5.46 |  |
|  | n1c(N)nc(C)cc1-c2c(Cl)cc(Cl)cc2OCCCC(F)(F)F | ([Zehnder et al., 2011](#_ENREF_32)) | 11,600 | 4.94 | 5.33 |  |
|  | c1cccc(c12)[nH]c(-c3ccccc3)c2C4CCN(CC4)CCc5ccccc5 | ([Rowley et al., 2001](#_ENREF_27)) | 80 | 7.09 | 6.94 | * |
|  | c1cccc(c12)[nH]c(-c3ccccc3)c2[C@@H](C[C@H]45)C[C@H](CC5)N4CCc6ccccc6 | ([Rowley et al., 2001](#_ENREF_27)) | 18 | 7.74 | 7.21 | * |
|  | c1cccc(c12)[nH]c(-c3ccccc3)c2C4CCNCC4 | ([Rowley et al., 2001](#_ENREF_27)) | 5,700 | 5.24 | 5.60 |  |
|  | c1cccc(c12)[nH]c(-c3ccccc3)c2[C@H]4CCCNC4 | ([Rowley et al., 2001](#_ENREF_27)) | 4,900 | 5.31 | 5.49 |  |
|  | c1cccc(c12)[nH]c(-c3ccccc3)c2[C@H]4CCCN(C4)C | ([Rowley et al., 2001](#_ENREF_27)) | 9,600 | 5.02 | 5.29 |  |
|  | c1cccc(c12)[nH]c(-c3ccccc3)c2[C@H]4CCCCN4C | ([Rowley et al., 2001](#_ENREF_27)) | 7,000 | 5.15 | 5.37 |  |
|  | c1cccc(c12)[nH]c(-c3ccccc3)c2[C@H]4CCCN(C4)Cc5ccccc5 | ([Rowley et al., 2001](#_ENREF_27)) | 290 | 6.54 | 6.56 |  |
|  | c1cccc(c12)[nH]c(-c3ccccc3)c2[C@H]4CCCN(C4)CCc5ccccc5 | ([Rowley et al., 2001](#_ENREF_27)) | 530 | 6.28 | 6.45 |  |
|  | c1cc(F)cc(c12)[nH]c(-c3ccccc3)c2[C@@H]4CNCC[C@H]4F | ([Rowley et al., 2001](#_ENREF_27)) | 4,000 | 5.40 | 5.88 |  |
|  | c1cc(Cl)cc(c12)[nH]c(-c3ccccc3)c2[C@@H]4CNCC[C@H]4F | ([Rowley et al., 2001](#_ENREF_27)) | 1,400 | 5.85 | 6.04 |  |
|  | Fc1cccc(c12)[nH]c(-c3ccccc3)c2[C@H]4C[C@H](F)CNC4 | ([Rowley et al., 2001](#_ENREF_27)) | 3,000 | 5.52 | 5.35 |  |
|  | Clc1cccc(c12)[nH]c(-c3ccccc3)c2[C@H]4C[C@H](F)CNC4 | ([Rowley et al., 2001](#_ENREF_27)) | 2,100 | 5.68 | 5.51 |  |
|  | C1NCC[C@@H](F)[C@@H]1c2c([nH]c(c23)cc(F)cc3)-c4cccc(c45)cccc5 | ([Rowley et al., 2001](#_ENREF_27)) | 110 | 6.96 | 6.65 | * |
|  | C1NCC[C@@H](F)[C@@H]1c2c([nH]c(c23)cc(F)cc3)-c(c4)ccc(c45)cccc5 | ([Rowley et al., 2001](#_ENREF_27)) | 500 | 6.30 | 6.48 |  |
|  | c1cc(F)cc(c12)[nH]c(-c3cccnc3)c2[C@H]4[C@H](F)CCNC4 | ([Rowley et al., 2001](#_ENREF_27)) | 2,500 | 5.60 | 5.91 |  |
|  | c1cc(F)cc(c12)[nH]c(-c3cscc3)c2[C@H]4[C@H](F)CCNC4 | ([Rowley et al., 2001](#_ENREF_27)) | 1,500 | 5.82 | 6.01 |  |
|  | c1cc(F)cc(c12)[nH]c(C3CCCCC3)c2[C@H]4[C@H](F)CCNC4 | ([Rowley et al., 2001](#_ENREF_27)) | 5,400 | 5.27 | 5.51 |  |
|  | c1cc(F)cc(c12)[nH]c(C(=O)OC)c2[C@H]3[C@H](F)CCNC3 | ([Rowley et al., 2001](#_ENREF_27)) | 8,200 | 5.09 | 5.57 |  |
|  | C1NCC[C@@H](F)[C@@H]1c2c([nH]c(c23)cc(F)cc3)C(=O)Nc4ccccc4 | ([Rowley et al., 2001](#_ENREF_27)) | 930 | 6.03 | 6.36 |  |
|  | c1cc(F)cc(c12)[nH]c(-c(cc3)ccc3F)c2[C@H]4[C@H](F)CCNC4 | ([Rowley et al., 2001](#_ENREF_27)) | 1,600 | 5.80 | 5.99 |  |
|  | c1cc(F)cc(c12)[nH]c(-c(cc3)ccc3F)c2[C@H]4CCCNC4 | ([Rowley et al., 2001](#_ENREF_27)) | 1,300 | 5.89 | 6.21 |  |
|  | c1cc([N+](=O)[O-])ccc1OCCNCCc(cc2)ccc2[N+](=O)[O-] | ([Yu et al., 2015](#_ENREF_31)) | 47 | 7.33 | 6.78 | * |
|  | c1cc([N+](=O)[O-])ccc1OCCN(C)CCc(cc2)ccc2[N+](=O)[O-] | ([Yu et al., 2015](#_ENREF_31)) | 24 | 7.62 | 7.23 | * |
|  | c1cc([N+](=O)[O-])ccc1OCCN(CC)CCc(cc2)ccc2[N+](=O)[O-] | ([Yu et al., 2015](#_ENREF_31)) | 4.2 | 8.38 | 7.79 | * |
|  | c1cc([N+](=O)[O-])ccc1OCCN(CCF)CCc(cc2)ccc2[N+](=O)[O-] | ([Yu et al., 2015](#_ENREF_31)) | 65 | 7.19 | 6.95 | * |
|  | c1cc([N+](=O)[O-])ccc1OCCN(CC#N)CCc(cc2)ccc2[N+](=O)[O-] | ([Yu et al., 2015](#_ENREF_31)) | 203 | 6.69 | 6.38 |  |
|  | c1cc([N+](=O)[O-])ccc1OCCN(CC(=O)[O-])CCc(cc2)ccc2[N+](=O)[O-] | ([Yu et al., 2015](#_ENREF_31)) | 588 | 6.23 | 6.07 |  |
|  | c1cc([N+](=O)[O-])ccc1OCCN(COC)CCc(cc2)ccc2[N+](=O)[O-] | ([Yu et al., 2015](#_ENREF_31)) | 251 | 6.60 | 6.72 |  |
|  | c1cc([N+](=O)[O-])ccc1OCCN(COC(F)(F)F)CCc(cc2)ccc2[N+](=O)[O-] | ([Yu et al., 2015](#_ENREF_31)) | 355 | 6.45 | 6.43 |  |
|  | c1cc([N+](=O)[O-])ccc1OCCCCN(C)CCc(cc2)ccc2[N+](=O)[O-] | ([Yu et al., 2015](#_ENREF_31)) | 1.6 | 8.79 | 7.98 | * |
|  | c1cc([N+](=O)[O-])ccc1OCCCCN(C)Cc(cc2)ccc2[N+](=O)[O-] | ([Yu et al., 2015](#_ENREF_31)) | 66 | 7.18 | 6.86 | * |
|  | c1cc(Cl)c(Cl)cc1OCCN(C)CCc(cc2)cc(Cl)c2Cl | ([Yu et al., 2015](#_ENREF_31)) | 5,571 | 5.25 | 5.51 |  |
|  | c1ccccc1OCCN(C)CCc(cc2)ccc2OC | ([Yu et al., 2015](#_ENREF_31)) | 944 | 6.02 | 6.18 |  |
|  | c1cc(Cl)ccc1C(=O)C2CCN(CC2)CCc(cc3Cl)ccc3Cl | ([Yu et al., 2015](#_ENREF_31)) | 54 | 7.27 | 6.88 | * |
|  | c1cc(C)ccc1C(=O)C2CCN(CC2)CCCc3ccccc3 | ([Yu et al., 2015](#_ENREF_31)) | 60 | 7.22 | 6.78 | * |
|  | c1ccccc1C(=O)N2CCN(CC2)CCc3ccccc3 | ([Yu et al., 2015](#_ENREF_31)) | 5,418 | 5.27 | 5.56 |  |
|  | c1cc(Cl)ccc1C(=O)[C@H](CC2)CC[N@@+]2(C)CCc3ccccc3 | ([Yu et al., 2015](#_ENREF_31)) | 48 | 7.32 | 7.13 | * |
|  | CCCCCCCN(CC)CCCCc(cc1)ccc1Cl | ([Yu et al., 2015](#_ENREF_31)) | 31 | 7.51 | 7.29 | * |
|  | CCCCCCCN(CC)C/C=C/Cc1ccccc1 | ([Yu et al., 2015](#_ENREF_31)) | 119 | 6.92 | 6.78 | * |
|  | CCCCCCCN(CC)CC#Cc(cc1)ccc1C | ([Yu et al., 2015](#_ENREF_31)) | 3,282 | 5.48 | 5.69 |  |
|  | CCCN(CC)CC#Cc(cc1)ccc1C | ([Yu et al., 2015](#_ENREF_31)) | 1,196 | 5.92 | 5.71 |  |
|  | CCCCCCCN(CC)CC#CC(c1ccccc1)c2ccccc2 | ([Yu et al., 2015](#_ENREF_31)) | 97 | 7.01 | 7.04 | * |
|  | CCCCCCC[N+](CC)(CC)CC#Cc(cc1)ccc1Cl | ([Yu et al., 2015](#_ENREF_31)) | 1.8 | 8.74 | 7.98 | * |
|  | CCCCCCCCc1nc(ccc1)CCCCCCCC | ([Yu et al., 2015](#_ENREF_31)) | 36,870 | 4.43 | 5.02 |  |
|  | CCCCCCc([n+]1C)cccc1CCCCCC | ([Yu et al., 2015](#_ENREF_31)) | 12 | 7.92 | 7.69 | * |
|  | CCCCCCCc([n+]1C)cccc1CCCCCCC | ([Yu et al., 2015](#_ENREF_31)) | 0.28 | 9.55 | 8.26 | * |
|  | CCCCCCCCc([n+]1C)cccc1CCCCCCCC | ([Yu et al., 2015](#_ENREF_31)) | 3.5 | 8.45 | 7.61 | * |
|  | CCCCCCCCCc([n+]1C)cccc1CCCCCCCCC | ([Yu et al., 2015](#_ENREF_31)) | 3.4 | 8.47 | 7.69 | * |
|  | CCCCCCCCCCc([n+]1C)cccc1CCCCCCCCCC | ([Yu et al., 2015](#_ENREF_31)) | 17 | 7.77 | 7.28 | * |
|  | CCCCCCCCCCCCc([n+]1C)cccc1CCCCCCCCCCCC | ([Yu et al., 2015](#_ENREF_31)) | 108 | 6.97 | 6.67 | * |
|  | CCCCCCCCCC#CCc([n+]1C)cccc1CC#CCCCCCCCCC | ([Yu et al., 2015](#_ENREF_31)) | 1,179 | 5.93 | 5.60 |  |
|  | Clc1ccc(cc1)OCCCc2nc(ccc2)CCCOc(cc3)ccc3Cl | ([Yu et al., 2015](#_ENREF_31)) | 3,654 | 5.44 | 5.36 |  |
|  | Clc1ccc(cc1)OCCCc([n+]2C)cccc2CCCOc(cc3)ccc3Cl | ([Yu et al., 2015](#_ENREF_31)) | 6 | 8.22 | 7.71 | * |
|  | Clc1ccc(cc1)OCC#Cc([n+]2C)cccc2C#CCOc(cc3)ccc3Cl | ([Yu et al., 2015](#_ENREF_31)) | 2,460 | 5.61 | 5.24 |  |
|  | c1ccccc1-c2ccc(cc2)CCc([n+]3C)cccc3CCc4ccc(cc4)-c5ccccc5 | ([Yu et al., 2015](#_ENREF_31)) | 63 | 7.20 | 7.32 | * |
|  | C1CCCCC1C#Cc([n+]2C)cccc2C#CC3CCCCC3 | ([Yu et al., 2015](#_ENREF_31)) | 1,208 | 5.92 | 6.24 |  |
|  | c1ccccc1C#Cc([n+]2C)cccc2C#Cc3ccccc3 | ([Yu et al., 2015](#_ENREF_31)) | 498 | 6.30 | 6.63 |  |
|  | c1ccccc1-c2ccc(cc2)C#Cc([n+]3C)cccc3C#Cc(cc4)ccc4-c5ccccc5 | ([Yu et al., 2015](#_ENREF_31)) | 86 | 7.07 | 6.79 | * |
|  | Cc1ccccc1-c2ccc(cc2)C#Cc([n+]3C)cccc3C#Cc(cc4)ccc4-c5ccccc5C | ([Yu et al., 2015](#_ENREF_31)) | 577 | 6.24 | 6.03 |  |
|  | c1c(C)cccc1-c2ccc(cc2)C#Cc([n+]3C)cccc3C#Cc(cc4)ccc4-c5cccc(c5)C | ([Yu et al., 2015](#_ENREF_31)) | 122 | 6.91 | 6.59 | * |
|  | COc(c1)cccc1-c2ccc(cc2)C#Cc([n+]3C)cccc3C#Cc(cc4)ccc4-c5cccc(c5)OC | ([Yu et al., 2015](#_ENREF_31)) | 176 | 6.75 | 6.41 |  |
|  | c1c(Cl)cccc1-c2ccc(cc2)C#Cc([n+]3C)cccc3C#Cc(cc4)ccc4-c5cccc(Cl)c5 | ([Yu et al., 2015](#_ENREF_31)) | 139 | 6.86 | 6.44 | * |
|  | Cc1ccc(cc1)-c2ccc(cc2)C#Cc([n+]3C)cccc3C#Cc(cc4)ccc4-c(cc5)ccc5C | ([Yu et al., 2015](#_ENREF_31)) | 303 | 6.52 | 6.25 |  |
|  | COc1ccc(cc1)-c2ccc(cc2)C#Cc([n+]3C)cccc3C#Cc(cc4)ccc4-c(cc5)ccc5OC | ([Yu et al., 2015](#_ENREF_31)) | 247 | 6.61 | 6.27 |  |
|  | Oc1ccc(cc1)-c2ccc(cc2)C#Cc([n+]3C)cccc3C#Cc(cc4)ccc4-c(cc5)ccc5O | ([Yu et al., 2015](#_ENREF_31)) | 153 | 6.81 | 6.51 |  |
|  | Clc1ccc(cc1)-c2ccc(cc2)C#Cc([n+]3C)cccc3C#Cc(cc4)ccc4-c(cc5)ccc5Cl | ([Yu et al., 2015](#_ENREF_31)) | 71 | 7.15 | 7.24 | * |
|  | c1cccc(c12)n(c(n2)C)[C@H](C3)C[C@@H](N([C@@H]34)C)COC4 | ([Armour et al., 2006](#_ENREF_4)) | 42 | 7.38 | 7.07 | * |
|  | c1cc(C#N)ccc1-c(cc2)ccc2N3CC[C@@H]([C@@H]34)CN(C4)C | ([Zhao et al., 2009](#_ENREF_33)) | 282 | 6.55 | 6.29 |  |
|  | C1CNCCc(c12)cc(cc2)S(=O)(=O)N(C)C[C@@H](CO3)c(c34)cccc4 | ([Fish et al., 2009](#_ENREF_12)) | 2,990 | 5.52 | 5.59 |  |
|  | C1CNCCc(c12)cc(cc2)S(=O)(=O)N3CC(C3)Oc4ccccc4Cl | ([Fish et al., 2009](#_ENREF_12)) | 3,410 | 5.47 | 5.67 |  |
|  | C1CNCCc(c12)cc(cc2)S(=O)(=O)N3CC(C3)Oc(c4)cccc4Cl | ([Fish et al., 2009](#_ENREF_12)) | 3,960 | 5.40 | 5.74 |  |
|  | C1CNCCc(c12)cc(cc2)S(=O)(=O)N3CC(C3)Oc(cc4)ccc4Cl | ([Fish et al., 2009](#_ENREF_12)) | 2,090 | 5.68 | 6.06 |  |
|  | C1CNCCc(c12)cc(cc2)S(=O)(=O)N3C[C@@H](CC3)Oc4ccccc4Cl | ([Fish et al., 2009](#_ENREF_12)) | 3,430 | 5.46 | 5.78 |  |
|  | C1CNCCc(c12)cc(cc2)S(=O)(=O)N3C[C@H](CC3)Oc4ccccc4Cl | ([Fish et al., 2009](#_ENREF_12)) | 5,240 | 5.28 | 5.52 |  |
|  | C1CNCCc(c12)cc(cc2)S(=O)(=O)N3CCC(CC3)Oc4ccccc4Cl | ([Fish et al., 2009](#_ENREF_12)) | 2,900 | 5.54 | 5.74 |  |
|  | Fc1ccc(cc1)Cn(c(c23)cccc2)c(n3)[C@@H]4CCCN(C4)C | ([Ravula et al., 2012](#_ENREF_24)) | 3,900 | 5.41 | 5.57 |  |
|  | Fc1ccc(cc1)Cn(c(c23)cccc2)c(n3)[C@@H]4CCCN(C4)CC(=O)N(C)C | ([Ravula et al., 2012](#_ENREF_24)) | 2,600 | 5.58 | 5.79 |  |
|  | C1N(C)CCC[C@H]1c(n2)n(c(c23)cccc3)CCOc4ccccc4 | ([Ravula et al., 2012](#_ENREF_24)) | 5,900 | 5.23 | 5.58 |  |
|  | c1cc(Cl)ccc1Oc(cc2)ccc2Nc3ncnc(n3)N | ([Bregman et al., 2012](#_ENREF_6)) | 2,300 | 5.64 | 5.87 |  |
|  | c1cc(Cl)ccc1Oc(cc2)ccc2Nc3nccc(n3)N | ([Bregman et al., 2012](#_ENREF_6)) | 430 | 6.37 | 6.24 |  |
|  | c1cc(Cl)ccc1Oc(cc2)ccc2Nc3ccnc(n3)N | ([Bregman et al., 2012](#_ENREF_6)) | 320 | 6.49 | 6.10 |  |
|  | c1cc(Cl)ccc1Oc(cc2)ccc2Nc3cncc(c3)N | ([Bregman et al., 2012](#_ENREF_6)) | 160 | 6.79 | 6.46 |  |
|  | c1cc(Cl)ccc1Oc(cc2)ccc2Nc3nccc(c3)N | ([Bregman et al., 2012](#_ENREF_6)) | 50 | 7.30 | 6.98 | * |
|  | c1cc(Cl)ccc1Oc(cc2)ccc2Nc3cncc(n3)N | ([Bregman et al., 2012](#_ENREF_6)) | 2,900 | 5.54 | 5.37 |  |
|  | c1cc(Cl)ccc1N(C)c(cc2)ccc2Nc3nccc(n3)N | ([Bregman et al., 2012](#_ENREF_6)) | 800 | 6.09 | 5.61 |  |
|  | c1cccc(c1Cl)Oc(cc2)ccc2Nc3nccc(n3)N | ([Bregman et al., 2012](#_ENREF_6)) | 1,400 | 5.85 | 5.59 |  |
|  | c1cc(F)ccc1Oc(cc2)ccc2Nc3nccc(n3)N | ([Bregman et al., 2012](#_ENREF_6)) | 1,900 | 5.72 | 5.57 |  |
|  | c1cc(C)ccc1Oc(cc2)ccc2Nc3nccc(n3)N | ([Bregman et al., 2012](#_ENREF_6)) | 1,100 | 5.96 | 5.84 |  |
|  | C1CC1c(cc2)ccc2Oc(cc3)ccc3Nc4nccc(n4)N | ([Bregman et al., 2012](#_ENREF_6)) | 500 | 6.30 | 6.07 |  |
|  | c1cc(C#N)ccc1Oc(cc2)ccc2Nc3nccc(n3)N | ([Bregman et al., 2012](#_ENREF_6)) | 1,200 | 5.92 | 5.64 |  |
|  | c1cc(C(F)(F)F)ccc1Oc(cc2)ccc2Nc3nccc(n3)N | ([Bregman et al., 2012](#_ENREF_6)) | 900 | 6.05 | 5.73 |  |
|  | FC(F)(F)Oc(cc1)ccc1Oc(cc2)ccc2Nc3nccc(n3)N | ([Bregman et al., 2012](#_ENREF_6)) | 100 | 7.00 | 6.71 | * |
|  | c1cc(F)ccc1Oc(cc2)ccc2Nc3cncc(n3)N | ([Bregman et al., 2012](#_ENREF_6)) | 1,100 | 5.96 | 5.68 |  |
|  | c1cc(C)ccc1Oc(cc2)ccc2Nc3cncc(n3)N | ([Bregman et al., 2012](#_ENREF_6)) | 2,000 | 5.70 | 5.44 |  |
|  | c1cc(C(F)(F)F)ccc1Oc(cc2)ccc2Nc3cncc(n3)N | ([Bregman et al., 2012](#_ENREF_6)) | 2,800 | 5.55 | 5.82 |  |
|  | n1c(C)ccc(c12)c(ccc2)N3CCN(CC3)CCc(c4)cccc4NS(=O)(=O)C | ([Leslie et al., 2010](#_ENREF_18)) | 501 | 6.30 | 6.17 |  |
|  | n1c(C)ccc(c12)c(ccc2)N3CCN(CC3)CCc(c4)cccc4NC(=O)C | ([Leslie et al., 2010](#_ENREF_18)) | 3,981 | 5.40 | 5.76 |  |
|  | n1c(C)ccc(c12)c(ccc2)N3CCN(CC3)CCc(c4)cccc4NC(=O)c5ccccc5 | ([Leslie et al., 2010](#_ENREF_18)) | 50 | 7.30 | 6.86 | * |
|  | n1c(C)ccc(c12)c(ccc2)N3CCN(CC3)CCc(c4)cccc4NC(=O)c5nc(C)sc5 | ([Leslie et al., 2010](#_ENREF_18)) | 316 | 6.50 | 6.69 |  |
|  | n1c(C)ccc(c12)c(ccc2)N3CCN(CC3)CCc(c4)cccc4NC(=O)NCC | ([Leslie et al., 2010](#_ENREF_18)) | 631 | 6.20 | 6.01 |  |
|  | n1c(C)ccc(c12)c(ccc2)N3CCN(CC3)CCc(c4)cccc4NC(=O)Nc5ccccc5 | ([Leslie et al., 2010](#_ENREF_18)) | 316 | 6.50 | 6.19 |  |
|  | n1c(C)ccc(c12)c(ccc2)N3CCN(CC3)CCc(c4)cccc4N5C(=O)NCC5 | ([Leslie et al., 2010](#_ENREF_18)) | 12,589 | 4.90 | 5.26 |  |
|  | n1c(C)ccc(c12)c(ccc2)N3CCN(CC3)CCc(c4)cccc4N5C(=O)OCC5 | ([Leslie et al., 2010](#_ENREF_18)) | 1,995 | 5.70 | 6.12 |  |
|  | CC(C)COc(cc1)cc2c1Oc(c3[C@]24N=C(N)OC4)ccc(c3)-c5cncnc5 | ([Epstein et al., 2014](#_ENREF_11)) | 1,840 | 5.73 | 5.99 |  |
|  | CC(C)(C)COc(cc1)cc2c1Oc(c3[C@]24N=C(N)OC4)ccc(c3)-c5cncnc5 | ([Epstein et al., 2014](#_ENREF_11)) | 660 | 6.18 | 6.49 |  |
|  | CC(C)(C)C#Cc(cc1)cc2c1Oc(c3[C@]24N=C(N)OC4)ccc(c3)-c5cncnc5 | ([Epstein et al., 2014](#_ENREF_11)) | 150 | 6.82 | 6.61 | * |
|  | CC(C)(C)CCc(cc1)cc2c1Oc(c3[C@]24N=C(N)OC4)ccc(c3)-c5cncnc5 | ([Epstein et al., 2014](#_ENREF_11)) | 410 | 6.39 | 6.65 |  |
|  | OC(C)(C)C#Cc(cc1)cc2c1Oc(c3[C@]24N=C(N)OC4)ccc(c3)-c5cncnc5 | ([Epstein et al., 2014](#_ENREF_11)) | 14,000 | 4.85 | 5.25 |  |
|  | c1ncncc1-c(c2)ccc(c2[C@]34N=C(N)OC4)Oc5c3cc(cc5)C#CC(C)(C)OC | ([Epstein et al., 2014](#_ENREF_11)) | 1,420 | 5.85 | 5.62 |  |
|  | c1ncncc1-c(c2)ccc(c2[C@]34N=C(N)OC4)Oc5c3cc(cc5)C6=CCOCC6 | ([Epstein et al., 2014](#_ENREF_11)) | 10,700 | 4.97 | 5.20 |  |
|  | c1ncncc1-c(c2)ccc(c2[C@]34N=C(N)OC4)Oc5c3cc(cc5)OCC6CCCCC6 | ([Epstein et al., 2014](#_ENREF_11)) | 90 | 7.04 | 6.82 | * |
|  | CC(F)(C)COc(cc1)cc2c1Oc(c3[C@]24N=C(N)OC4)ccc(c3)-c5cncnc5 | ([Epstein et al., 2014](#_ENREF_11)) | 4,980 | 5.30 | 5.57 |  |
|  | c1ncncc1-c(c2)ccc(c2[C@]34N=C(N)OC4)Oc5c3cc(cc5)OCC(C)(C#N)C | ([Epstein et al., 2014](#_ENREF_11)) | 11,700 | 4.93 | 5.25 |  |
|  | CC(C)(C)COc(cc1)cc2c1Oc(c3[C@]24N=C(N)OC4)ccc(c3)-c5ncccc5 | ([Epstein et al., 2014](#_ENREF_11)) | 496 | 6.30 | 6.45 |  |
|  | CC(C)(C)COc(cc1)cc2c1Oc(c3[C@]24N=C(N)OC4)ccc(c3)-c5ccncc5 | ([Epstein et al., 2014](#_ENREF_11)) | 624 | 6.20 | 6.33 |  |
|  | CC(C)(C)COc(cc1)cc2c1Oc(c3[C@]24N=C(N)OC4)ccc(c3)-c5cnccc5 | ([Epstein et al., 2014](#_ENREF_11)) | 797 | 6.10 | 5.99 |  |
|  | Fc1ncc(cc1)-c(c2)ccc(c2[C@]34N=C(N)OC4)Oc5c3cc(cc5)OCC(C)(C)C | ([Epstein et al., 2014](#_ENREF_11)) | 259 | 6.59 | 6.34 |  |
|  | CC(C)(C)COc(cc1)cc2c1Oc(c3[C@]24N=C(N)OC4)ccc(c3)-c5cncc(c5)C#N | ([Epstein et al., 2014](#_ENREF_11)) | 145 | 6.84 | 6.62 | * |
|  | CC(C)(C)COc(cc1)cc2c1Oc(c3[C@]24N=C(N)OC4)ccc(c3)-c5cncc(F)c5 | ([Epstein et al., 2014](#_ENREF_11)) | 377 | 6.42 | 6.21 |  |
|  | c1ccnc(F)c1-c(c2)ccc(c2[C@]34N=C(N)OC4)Oc5c3cc(cc5F)C6=CCOCC6 | ([Epstein et al., 2014](#_ENREF_11)) | 3,280 | 5.48 | 5.19 |  |
|  | c1ccnc(F)c1-c(c2)ccc(c2[C@]34N=C(N)OC4)Oc5c3cc(cc5F)-c6ccccn6 | ([Epstein et al., 2014](#_ENREF_11)) | 2,770 | 5.56 | 5.92 |  |
|  | CC(C)CN(C1=O)C(=O)CN1Cc(cc2)ccc2-c(n3)cccc3CN4CCCCC4 | ([van der Stelt et al., 2011](#_ENREF_29)) | 3,162 | 5.50 | 5.79 |  |
|  | c1ccccc1Cn(c(C)c2C)c(c23)ncnc3OC4CCN(CC4)Cc5cscn5 | ([Chakka et al., 2012](#_ENREF_8)) | 2,500 | 5.60 | 5.98 |  |
|  | c1ccccc1Cn(cc2C)c(c23)ncnc3OC4CCN(CC4)Cc5cscn5 | ([Chakka et al., 2012](#_ENREF_8)) | 1,400 | 5.85 | 6.19 |  |
|  | c1ccccc1Cn(cc2F)c(c23)ncnc3OC4CCN(CC4)Cc5cscn5 | ([Chakka et al., 2012](#_ENREF_8)) | 2,500 | 5.60 | 5.29 |  |
|  | c1ccccc1Cn(cc2Cl)c(c23)ncnc3OC4CCN(CC4)Cc5cscn5 | ([Chakka et al., 2012](#_ENREF_8)) | 1,500 | 5.82 | 5.81 |  |
|  | c1ccccc1Cn(cc2)c(c23)nc(C(F)(F)F)nc3OC4CCN(CC4)Cc5cscn5 | ([Chakka et al., 2012](#_ENREF_8)) | 990 | 6.01 | 5.64 |  |
|  | Fc1ccccc1Cn(cc2)c(c23)ncnc3OC4CCN(CC4)Cc5cscn5 | ([Chakka et al., 2012](#_ENREF_8)) | 1,500 | 5.82 | 5.69 |  |
|  | c1c(F)cccc1Cn(cc2)c(c23)ncnc3OC4CCN(CC4)Cc5cscn5 | ([Chakka et al., 2012](#_ENREF_8)) | 1,100 | 5.96 | 5.45 |  |
|  | c1c(Cl)cccc1Cn(cc2)c(c23)ncnc3OC4CCN(CC4)Cc5cscn5 | ([Chakka et al., 2012](#_ENREF_8)) | 1,200 | 5.92 | 5.34 |  |
|  | Cc1ccccc1Cn(cc2)c(c23)ncnc3OC4CCN(CC4)Cc5cscn5 | ([Chakka et al., 2012](#_ENREF_8)) | 3,200 | 5.49 | 5.84 |  |
|  | c1c(C)cccc1Cn(cc2)c(c23)ncnc3OC4CCN(CC4)Cc5cscn5 | ([Chakka et al., 2012](#_ENREF_8)) | 930 | 6.03 | 5.76 |  |
|  | Cc1ccc(cc1)Cn(cc2)c(c23)ncnc3OC4CCN(CC4)Cc5cscn5 | ([Chakka et al., 2012](#_ENREF_8)) | 2,750 | 5.56 | 5.27 |  |
|  | FC(F)(F)c1ccccc1Cn(cc2)c(c23)ncnc3OC4CCN(CC4)Cc5cscn5 | ([Chakka et al., 2012](#_ENREF_8)) | 2,460 | 5.61 | 5.92 |  |
|  | FC(F)(F)c(c1)cccc1Cn(cc2)c(c23)ncnc3OC4CCN(CC4)Cc5cscn5 | ([Chakka et al., 2012](#_ENREF_8)) | 600 | 6.22 | 6.03 |  |
|  | Fc1c(F)cccc1Cn(cc2)c(c23)ncnc3OC4CCN(CC4)Cc5cscn5 | ([Chakka et al., 2012](#_ENREF_8)) | 2,300 | 5.64 | 5.22 |  |
|  | Fc1c(Cl)cccc1Cn(cc2)c(c23)ncnc3OC4CCN(CC4)Cc5cscn5 | ([Chakka et al., 2012](#_ENREF_8)) | 950 | 6.02 | 6.06 |  |
|  | Fc1c(C(F)(F)F)cccc1Cn(cc2)c(c23)ncnc3OC4CCN(CC4)Cc5cscn5 | ([Chakka et al., 2012](#_ENREF_8)) | 300 | 6.52 | 6.18 |  |
|  | Fc1cccc(F)c1Cn(cc2)c(c23)ncnc3OC4CCN(CC4)Cc5cscn5 | ([Chakka et al., 2012](#_ENREF_8)) | 2,300 | 5.64 | 5.33 |  |
|  | c1ccccc1Cn(cc2)c(c23)ncnc3OC4CCN(CC4)Cc5ccccc5 | ([Chakka et al., 2012](#_ENREF_8)) | 1,200 | 5.92 | 5.63 |  |
|  | c1ccccc1Cn(cc2)c(c23)ncnc3OC4CCN(CC4)Cc(n5)cccc5C | ([Chakka et al., 2012](#_ENREF_8)) | 2,400 | 5.62 | 5.42 |  |
|  | c1ccccc1Cn(cc2)c(c23)ncnc3OC4CCN(CC4)Cc(cc5)ncc5C | ([Chakka et al., 2012](#_ENREF_8)) | 980 | 6.01 | 6.46 |  |
|  | c1ccccc1Cn(cc2)c(c23)ncnc3OC4CCN(CC4)Cc5nccc(c5)C | ([Chakka et al., 2012](#_ENREF_8)) | 2,200 | 5.66 | 5.34 |  |
|  | c1ccccc1Cn(cc2)c(c23)ncnc3OC4CCN(CC4)Cc5ncccc5C | ([Chakka et al., 2012](#_ENREF_8)) | 730 | 6.14 | 5.81 |  |
|  | c1ccccc1Cn(cc2)c(c23)ncnc3OC4CCN(CC4)Cc(n5)cccc5F | ([Chakka et al., 2012](#_ENREF_8)) | 2,900 | 5.54 | 5.79 |  |
|  | c1ccccc1Cn(cc2)c(c23)ncnc3OC4CCN(CC4)Cc(cc5)ncc5 | ([Chakka et al., 2012](#_ENREF_8)) | 1,100 | 5.96 | 6.28 |  |
|  | Fc1ccccc1Cn(cc2)c(c23)ncnc3OC4CCN(CC4)Cc5ccccn5 | ([Chakka et al., 2012](#_ENREF_8)) | 1,800 | 5.74 | 6.03 |  |
|  | Fc1cccc(c1F)Cn(cc2)c(c23)ncnc3OC4CCN(CC4)Cc5ccccn5 | ([Chakka et al., 2012](#_ENREF_8)) | 1,000 | 6.00 | 5.73 |  |
|  | Clc1cccc(c1F)Cn(cc2)c(c23)ncnc3OC4CCN(CC4)Cc5ccccn5 | ([Chakka et al., 2012](#_ENREF_8)) | 680 | 6.17 | 5.67 |  |
|  | FC(F)(F)c1cccc(c1F)Cn(cc2)c(c23)ncnc3OC4CCN(CC4)Cc5ccccn5 | ([Chakka et al., 2012](#_ENREF_8)) | 320 | 6.49 | 6.13 |  |
|  | c1cc(Cl)ccc1CC(=O)N[C@H](Cc(cc2)ccc2Cl)C(=O)N[C@H](C[C@H]34)C[C@H](N4)CC3 | ([Napier et al., 2011](#_ENREF_22)) | 4,169 | 5.38 | 5.02 |  |
|  | c1cc(Cl)ccc1C2(CC2)C(=O)N[C@H](Cc(cc3)ccc3Cl)C(=O)N[C@H](C[C@H]45)C[C@H](N5)CC4 | ([Napier et al., 2011](#_ENREF_22)) | 1,585 | 5.80 | 5.56 |  |
|  | Nc1nccc(c12)cc(cc2)CNCCc3ccccc3 | ([Ray et al., 2011](#_ENREF_25)) | 3,162 | 5.50 | 5.21 |  |
|  | c1cnc(N)c(c12)ccc(c2)O[C@H]3CCCNC3 | ([Ray et al., 2011](#_ENREF_25)) | 19,953 | 4.70 | 5.03 |  |
|  | c1cnc(N)c(c12)ccc(c2)O[C@H](C3)CCCN3Cc4ccccc4 | ([Ray et al., 2011](#_ENREF_25)) | 794 | 6.10 | 5.87 |  |
|  | c1nccc(c12)c(ccc2)S(=O)(=O)N3CCCNCC3  **Fasudil** | ([Ray et al., 2011](#_ENREF_25)) | 25,119 | 4.60 | 4.98 |  |
|  | O=c1[nH]ccc(c12)c(ccc2)S(=O)(=O)N3CCCNCC3  **HO-Fasudil** | ([Ray et al., 2011](#_ENREF_25)) | 7,943 | 5.10 | 5.38 |  |
|  | [nH]1ccc(c12)cc(cc2)-c3ccc(cn3)N(C4)C[C@@H]([C@@H]45)N(C5)C **A-859261** | ([Gao et al., 2012](#_ENREF_13)) | 1,900 | 5.72 | 5.96 |  |

**References**

Adam, J.M., Clark, J.K., Davies, K., Everett, K., Fields, R., Francis, S., et al. (2012). Low brain penetrant CB1 receptor agonists for the treatment of neuropathic pain. *Bioorg Med Chem Lett* 22(8)**,** 2932-2937. doi: 10.1016/j.bmcl.2012.02.048.

Andrews, M., Brown, A., Chiva, J.Y., Fradet, D., Gordon, D., Lansdell, M., et al. (2009a). Design and optimisation of selective serotonin re-uptake inhibitors with high synthetic accessibility: Part 2. *Bioorg Med Chem Lett* 19(20)**,** 5893-5897. doi: 10.1016/j.bmcl.2009.08.066.

Andrews, M., Brown, A., Chiva, J.Y., Fradet, D., Gordon, D., Lansdell, M., et al. (2009b). Design and optimization of selective serotonin re-uptake inhibitors with high synthetic accessibility Part 1. *Bioorg Med Chem Lett* 19(8)**,** 2329-2332. doi: 10.1016/j.bmcl.2009.02.054.

Armour, D.R., de Groot, M.J., Price, D.A., Stammen, B.L.C., Wood, A., Perros, M., et al. (2006). The discovery of tropane-derived CCR5 receptor antagonists. *Chem Biol Drug Des* 67(4)**,** 305-308. doi: 10.1111/j.1747-0285.2006.00376.x.

Black, L.A., Liu, H., Diaz, G.J., Fox, G.B., Browman, K.E., Wetter, J., et al. (2008). Minimization of potential hERG liability in histamine H-3 receptor antagonists. *Inflammation Research* 57**,** S45-S46. doi: 10.1007/s00011-007-0622-2.

Bregman, H., Nguyen, H.N., Feric, E., Ligutti, J., Liu, D., McDermott, J.S., et al. (2012). The discovery of aminopyrazines as novel, potent Na(v)1.7 antagonists: Hit-to-lead identification and SAR. *Bioorg Med Chem Lett* 22(5)**,** 2033-2042. doi: 10.1016/j.bmcl.2012.01.023.

Bromidge, S.M., Arban, R., Bertani, B., Bison, S., Borriello, M., Cavanni, P., et al. (2010). Design and Synthesis of Novel Tricyclic Benzoxazines as Potent 5-HT1A/B/D Receptor Antagonists Leading to the Discovery of 6-{2-[4-(2-methyl-5-quinolinyl)-1-piperazinyl]ethyl}-4H-imidazo-[5,1-c][1,4]benzoxazine-3-carboxamide (GSK588045). *J Med Chem* 53(15)**,** 5827-5843. doi: 10.1021/jm100482n.

Chakka, N., Bregman, H., Du, B.F., Nguyen, H.N., Buchanan, J.L., Feric, E., et al. (2012). Discovery and hit-to-lead optimization of pyrrolopyrimidines as potent, state-dependent Na(v)1.7 antagonists. *Bioorg Med Chem Lett* 22(5)**,** 2052-2062. doi: 10.1016/j.bmcl.2012.01.015.

Chen, J.J., Liu, Q.Y., Yuan, C., Gore, V., Lopez, P., Ma, V., et al. (2015). Development of 2-aminooxazoline 3-azaxanthenes as orally efficacious beta-secretase inhibitors for the potential treatment of Alzheimer's disease. *Bioorg Med Chem Lett* 25(4)**,** 767-774. doi: 10.1016/j.bmcl.2014.12.092.

Dineen, T.A., Chen, K., Cheng, A.C., Derakhchan, K., Epstein, O., Esmay, J., et al. (2014). Inhibitors of beta-Site Amyloid Precursor Protein Cleaving Enzyme (BACE1): Identification of (S)-7-(2-Fluoropyridin-3-yl)-3-((3-methyloxetan-3-yl)ethynyl)-5 ' H-spiro[chromeno[2,3-b]pyridine-5,4 '-oxazol]-2 '-amine (AMG-8718). *J Med Chem* 57(23)**,** 9811-9831. doi: 10.1021/jm5012676.

Epstein, O., Bryan, M.C., Cheng, A.C., Derakhchan, K., Dineen, T.A., Hickman, D., et al. (2014). Lead Optimization and Modulation of hERG Activity in a Series of Aminooxazoline Xanthene beta-Site Amyloid Precursor Protein Cleaving Enzyme (BACE1) Inhibitors. *J Med Chem* 57(23)**,** 9796-9810. doi: 10.1021/jm501266w.

Fish, P.V., Brown, A.D., Evrard, E., and Roberts, L.R. (2009). 7-Sulfonamido-3-benzazepines as potent and selective 5-HT2C receptor agonists: Hit-to-lead optimization. *Bioorg Med Chem Lett* 19(7)**,** 1871-1875. doi: 10.1016/j.bmcl.2009.02.071.

Gao, Y.J., Ravert, H.T., Valentine, H., Scheffel, U., Finley, P., Wong, D.F., et al. (2012). 5-(5-(6-[C-11]methyl-3,6-diazabicyclo[3.2.0]heptan-3-yl)pyridin-2-yl)-1H-indole as a potential PET radioligand for imaging cerebral alpha 7-nAChR in mice. *Bioorg Med Chem* 20(12)**,** 3698-3702. doi: 10.1016/j.bmc.2012.04.056.

Graham, J.M., Coughenour, L.L., Barr, B.M., Rock, D.L., and Nikam, S.S. (2008). 1-Aminoindanes as novel motif with potential atypical antipsychotic properties. *Bioorg Med Chem Lett* 18(2)**,** 489-493. doi: 10.1016/j.bmcl.2007.11.106.

Ladduwahetty, T., Gilligan, M., Humphries, A., Merchant, K.J., Fish, R., McAlister, G., et al. (2010). Non-basic ligands for aminergic GPCRs: The discovery and development diaryl sulfones as selective, orally bioavailable 5-HT2A receptor antagonists for the treatment of sleep disorders. *Bioorg Med Chem Lett* 20(12)**,** 3708-3712. doi: 10.1016/j.bmcl.2010.04.090.

Lansdell, M.I., Hepworth, D., Calabrese, A., Brown, A.D., Blagg, J., Burring, D.J., et al. (2010). Discovery of a Selective Small-Molecule Melanocortin-4 Receptor Agonist with Efficacy in a Pilot Study of Sexual Dysfunction in Humans. *J Med Chem* 53(8)**,** 3183-3197. doi: 10.1021/jm9017866.

Lavrador-Erb, K., Ravula, S.B., Yu, J.H., Zamani-Kord, S., Moree, W.J., Petroski, R.E., et al. (2010). The discovery and structure-activity relationships of 2-(piperidin-3-yl)-1H-benzimidazoles as selective, CNS penetrating H-1-antihistamines for insomnia. *Bioorg Med Chem Lett* 20(9)**,** 2916-2919. doi: 10.1016/j.bmcl.2010.03.027.

Leslie, C.P., Biagetti, M., Bison, S., Bromidge, S.M., Di Fabio, R., Donati, D., et al. (2010). Discovery of 1-(3-{2-[4-(2-Methyl-5-quinolinyl)-1-piperazinyl]ethyl}phenyl)-2-imidazolidinone (GSK163090), a Potent, Selective, and Orally Active 5-HT1A/B/D Receptor Antagonist. *J Med Chem* 53(23)**,** 8228-8240. doi: 10.1021/jm100714c.

Lowe, J.A., DeNinno, S.L., Coe, J.W., Zhang, L., Mente, S., Hurst, R.S., et al. (2010). A novel series of [3.2.1] azabicyclic biaryl ethers as alpha 3 beta 4 and alpha 6/4 beta 4 nicotinic receptor agonists. *Bioorg Med Chem Lett* 20(16)**,** 4749-4752. doi: 10.1016/j.bmcl.2010.06.142.

Micheli, F., Cavanni, P., Arban, R., Benedetti, R., Bertani, B., Bettati, M., et al. (2010). 1-(Aryl)-6-[alkoxyalkyl]-3-azabicyclo[3.1.0]hexanes and 6-(Aryl)-6-[alkoxyalkyl]-3-azabicyclo[3.1.0]hexanes: A New Series of Potent and Selective Triple Reuptake Inhibitors. *J Med Chem* 53(6)**,** 2534-2551. doi: 10.1021/jm901818u.

Murphy, S.T., Case, H.L., Ellsworth, E., Hagen, S., Huband, M., Joannides, T., et al. (2007). The synthesis and biological evaluation of novel series of nitrile-containing fluoroquinolones as antibacterial agents. *Bioorg Med Chem Lett* 17(8)**,** 2150-2155. doi: 10.1016/j.bmcl.2007.01.090.

Napier, S., Wishart, G., Arbuckle, W., Baker, J., Barn, D., Bingham, M., et al. (2011). The discovery of novel 8-azabicyclo[3.2.1]octan-3-yl)-3-(4-chlorophenyl) propanamides as vasopressin V-1A receptor antagonists. *Bioorg Med Chem Lett* 21(10)**,** 3163-3167. doi: 10.1016/j.bmcl.2011.02.096.

Rankovic, Z., Cai, J., Kerr, J., Fradera, X., Robinson, J., Mistry, A., et al. (2010). Optimisation of 2-cyano-pyrimidine inhibitors of cathepsin K: Improving selectivity over hERG. *Bioorg Med Chem Lett* 20(21)**,** 6237-6241. doi: 10.1016/j.bmcl.2010.08.101.

Ravula, S.B., Yu, J.H., Tran, J.A., Arellano, M., Tucci, F.C., Moree, W.J., et al. (2012). Lead optimization of 2-(piperidin-3-yl)-1H-benzimidazoles: Identification of 2-morpholin- and 2-thiomorpholin-2-yl-1H-benzimidazoles as selective and CNS penetrating H-1-antihistamines for insomnia. *Bioorg Med Chem Lett* 22(1)**,** 421-426. doi: 10.1016/j.bmcl.2011.10.115.

Ray, P., Wright, J., Adam, J., Bennett, J., Boucharens, S., Black, D., et al. (2011). Fragment-based discovery of 6-substituted isoquinolin-1-amine based ROCK-I inhibitors. *Bioorg Med Chem Lett* 21(1)**,** 97-101. doi: 10.1016/j.bmcl.2010.11.060.

Reid, M., Carlyle, I., Caulfield, W.L., Clarkson, T.R., Cusick, F., Epemolu, O., et al. (2010). The discovery and SAR of indoline-3-carboxamides-A new series of 5-HT6 antagonists. *Bioorg Med Chem Lett* 20(12)**,** 3713-3716. doi: 10.1016/j.bmcl.2010.04.085.

Rowley, M., Hallett, D.J., Goodacre, S., Moyes, C., Crawforth, J., Sparey, T.J., et al. (2001). 3-(4-fluoropiperidin-3-yl)-2-phenylindoles as high affinity, selective, and orally bioavailable h5-HT2A receptor antagonist. *J Med Chem* 44(10)**,** 1603-1614. doi: 10.1021/jm0004998.

Schrimpf, M.R., Sippy, K.B., Briggs, C.A., Anderson, D.J., Li, T., Ji, J.G., et al. (2012). SAR of alpha 7 nicotinic receptor agonists derived from tilorone: Exploration of a novel nicotinic pharmacophore. *Bioorg Med Chem Lett* 22(4)**,** 1633-1638. doi: 10.1016/j.bmcl.2011.12.126.

van der Stelt, M., Cals, J., Broeders-Josten, S., Cottney, J., van der Doelen, A.A., Hermkens, M., et al. (2011). Discovery and Optimization of 1-(4-(Pyridin-2-yl)benzyl)imidazolidine-2,4-dione Derivatives As a Novel Class of Selective Cannabinoid CB2 Receptor Agonists. *J Med Chem* 54(20)**,** 7350-7362. doi: 10.1021/jm200916p.

Wilson, K.J., van Niel, M.B., Cooper, L., Bloomfield, D., O'Connor, D., Fish, R., et al. (2007). 2,5-Disubstituted pyridines: The discovery of a novel series of 5-HT2A ligands. *Bioorg Med Chem Lett* 17(9)**,** 2643-2648. doi: 10.1016/j.bmcl.2007.01.098.

Yu, Z., van Veldhoven, J.P.D., Louvel, J., 't Hart, I.M.E., Rook, M.B., van der Heyden, M.A.G., et al. (2015). Structure-Affinity Relationships (SARs) and Structure-Kinetics Relationships (SKRs) of K(v)11.1 Blockers. *J Med Chem* 58(15)**,** 5916-5929. doi: 10.1021/acs.jmedchem.5b00518.

Zehnder, L., Bennett, M., Meng, J., Huang, B.W., Ninkovic, S., Wang, F., et al. (2011). Optimization of Potent, Selective, and Orally Bioavailable Pyrrolodinopyrimidine-Containing Inhibitors of Heat Shock Protein 90. Identification of Development Candidate 2-Amino-4-{4-chloro-2-[2-(4-fluoro-1H-pyrazol-1-yl)ethoxy]-6-methylphenyl}-N-(2,2-difluoropropyl)-5,7-dihydro-6H-pyrrolo [3,4-d]pyrimidine-6-carboxamide. *J Med Chem* 54(9)**,** 3368-3385. doi: 10.1021/jm200128m.

Zhao, C., Sun, M.H., Bennani, Y.L., Miller, T.R., Witte, D.G., Esbenshade, T.A., et al. (2009). Design of a New Histamine H-3 Receptor Antagonist Chemotype: (3aR,6aR)-5-Alkyl-1-aryl-octahydropyrrolo[3,4-b]pyrroles, Synthesis, and Structure-Activity Relationships. *J Med Chem* 52(15)**,** 4640-4649. doi: 10.1021/jm900480x.
