# Supplementary material for: Computational Tool for Fast in silico Evaluation of hERG K+ Channel Affinity
Source: Front Chem. 2017 Feb 23;5:7. doi: 10.3389/fchem.2017.00007 (PMC5408157; doi:10.3389/fchem.2017.00007)
Supplement: Supplementary file 3 [file Table3.DOCX]

**Table S3** Active compounds used for generating a decoys set.

| **Compounds** | |
| --- | --- |
| **1** | c1cc(OC)ccc1CCN(CC2)CCC2Nc(n3)n(Cc(cc4)ccc4F)c(c35)cccc5 **Astemizole** |
| **2** | Fc1ccc(cc1)Cn(c(c23)cccc2)c(n3)N(CC4)CCC4N(C)CC5CCOCC5 |
| **3** | Fc1ccc(cc1)Cn(c(c23)cccc2)c(n3)N(CC4)CCC4N(C)CCOC |
| **4** | O=S(=O)(C)Nc1ccc(cc1)OCCN(C)CCc(cc2)ccc2NS(=O)(=O)C **Dofetilide** |
| **5** | C1CCCCC1c2ccc(cc2)OCCCN3CCCCC3 |
| **6** | s1cccc1-c2ccc(cc2)OCCCN3CCCCC3 |
| **7** | CCOC(=O)c1ccc(cc1)-c2ccc(cc2)OCCCN3CCCCC3 |
| **8** | O1COc(c12)cc(cc2)-c3ccc(cc3)OCCCN4CCCCC4 |
| **9** | Nc1ccnc(c1)N(CC2)CCC2(c3ccccc3)C(=O)N(CC4)Cc(c45)cccc5 |
| **10** | Nc1ccnc(c1)N(CC2)CCC2(c3ccccc3)C(=O)Nc(n4)ccc(c45)cccc5 |
| **11** | Clc1cnc(cc1)NC(=O)c(cc2Cl)c(cc2)NC(=O)c3ccc(cc3)/C(=N/[H])N4CCC(CC4)C(=O)OCC |
| **12** | CCOC(=O)[C@@H](CCC1)CN1C(=N/[H])\c(cc2)ccc2C(=O)Nc(cc3)c(cc3Cl)C(=O)Nc(cc4)ncc4Cl |
| **13** | Clc1cnc(cc1)NC(=O)c(cc2Cl)c(c(c2)OC)NC(=O)c3ccc(cc3F)/C(=N/[H])N4CCC(CC4)C(=O)OCC |
| **14** | Clc1ccc(cc1)CCCC[N+](CC)(CC)CCCCCCC **Clofilium** |
| **15** | C1CC[C@@H](C)N1CCc(cc2)cc(c23)ccc(n3)-c(c4)nc(nc4C)-c5ccccc5 |
| **16** | c1c(N)ccnc1N(CC2)CCC23c4c(cccc4)[C@@H](O3)c5ccccc5 |
| **17** | c1cc(OC)cc(CC2)c1OC23CCN(CC3)CCc(c(F)c4)ccc4F |
| **18** | c1cc(F)ccc1C(c(cc2)ccc2F)CCCN(CC3)CCC3n4c(=O)[nH]c(c45)cccc5 **Pimozide** |
| **19** | c1cc(Cl)cc(c12)c(cc(=O)n2C)NC3CCN(CC3)C/C=C/c4ccccc4 |
| **20** | O=S(=O)(N)NCCC(CC1)CCN1c(nc(n2)CC)c(c23)cc(OC)c(c3)OC |
| **21** | Cc1cc(Cl)c(c(Cl)c1)OCCOc2ccc(cc2)[C@H]3CCNC[C@@H]3C(=O)N(C4CC4)Cc(c5)cc(CCCOC)cc5OC[C@@H]6C[C@@H]6C(=O)OCOC(=O)C(C)(C)C |
| **22** | CS(=O)(=O)Nc1ccc(cc1)C(=O)C2CCN(CC2)CCc(n3)cccc3C **E4031** |
| **23** | CN(C)C(=N/[H])\c(cc1)ccc1C(=O)Nc(cc2)c(C(=O)Nc(cc3)ncc3Cl)cc2Sc4ccccc4 |
| **24** | CN(C)C(=N/[H])\c(cc1)ccc1C(=O)Nc(cc2)c(C(=O)Nc(cc3)ncc3Cl)cc2Sc(cc4)ccc4C(=O)OC |
| **30** | [H]\N=C(N(C)C)\c(cc1)ccc1C(=O)Nc(cc2)c(cc2Cl)C(=O)Nc(cc3)ncc3Cl |
| **33** | C1CCCCN1c2cc(/C(=N/[H])N(C)C)ccc2C(=O)Nc(cc3)c(cc3Cl)C(=O)Nc(cc4)ncc4Cl |
| **38** | CN(C)C(=N/[H])\c(cc1)ccc1CN(C(=O)C2)[C@@H](C(=O)OCC)CN2S(=O)(=O)c(c3)[nH]c(c34)ccc(Cl)c4 |
| **52** | Fc1ccc(cc1)C(=O)CCCN2CC=C(CC2)n(c(=O)[nH]3)c(c34)cccc4 **Droperidol** |
| **53** | c1ccccc1C(c2ccccc2)OC(CC3)CCN3CCCC(=O)c(cc4)ccc4C(C)(C)C **Ebastine** |
| **56** | c1cc(F)ccc1C(c(cc2)ccc2F)OCCN3CCN(CC3)CCCc4ccccc4 **Vanoxerine_GBR-12909** |
| **63** | c1cc(C)nc(c12)cccc2N(CC3)CCN3CCc(c4)ccc(c45)OCC(=O)N5 |
| **67** | c1cc(C)nc(c12)cccc2N(CC3)CCN3CCc(cc4)cc(c45)OCC(=O)N5 |
| **103** | c1cccc(CC2)c1OC23CCN(CC3)CCc(c(F)c4)ccc4F |
| **104** | c1cccc(CC2)c1CC23CCN(CC3)CCc(c(F)c4)ccc4F |
| **113** | Clc1ccc(cc1)S(=O)(=O)C(CC2)CCN2CCc(cc3)c(F)cc3F |
| **118** | o1cccc1-c2ccc(cc2)OCCCN3CCCCC3 |
| **128** | COc1ccccc1-c2ccc(cc2)OCCCN3CCCCC3 |
| **132** | O=S(=O)(N)NCCC(CC1)CCN1c(ncn2)c(c23)ccc(c3)OC |
| **151** | Fc1ccc(cc1)Cn(c(c23)cccc2)c(n3)N(CC4)CCC4N(C)C |
| **152** | Cc1ccc(cc1)Cn(c(c23)cccc2)c(n3)N(CC4)CCC4N(C)C |
| **154** | COc1ccc(cc1)Cn(c(c23)cccc2)c(n3)N(CC4)CCC4N(C)CC5CCOCC5 |
| **155** | Fc1ccc(cc1)Cn(c(c23)cccc2)c(n3)N(CC4)CCC4N(C)CCO |
| **160** | Fc1ccc(cc1)Cn(c(c23)cccc2)c(n3)N(CC4)CCC4n5ccnc5 |
| **187** | c1c(Cl)ccc(c12)[nH]c(=O)cc2NC3CCN(CC3)Cc(c4)ccc(c45)OCO5 |
| **193** | c1cccnc1Cn2c(=O)cc(c(c23)cc(Cl)cc3)NC4CCN(CC4)Cc(c5)ccc(c56)cccc6 |
| **200** | FC(F)(F)Cn1c(=O)nc(c(c12)cc(Cl)cc2)NC3CCN(CC3)Cc(c4)ccc(c45)cccc5 |
| **202** | c1c(Cl)ccc(c12)n(CCCCC)c(=O)nc2NC3CCN(CC3)Cc(c4)ccc(c45)OCO5 |
| **204** | c1c(Cl)ccc(c12)n(CC(F)(F)F)c(=O)nc2N[C@H](C[C@H]34)C[C@@H](CC3)N4c(c5)ccc(c56)OCO6 |
| **208** | c1cccc(c12)n(CC(=O)NC)c(=O)n2C3CCN(CC3)CC[C@@H](C(C)C)Oc4cc(OC)ccc4C |
| **210** | C1C[C@@H](N2)C[C@@H](C[C@H]12)Oc(cc(c3)C#N)nc3-c4ccccc4 |
| **269** | C1CC[C@@H](C)N1CCc(cc2)cc(c23)ccc(n3)-c4c(C)n(nc4)-c(cc5Cl)ccc5Cl |
| **292** | Nc1ccnc(c1)N(CC2)CCC2(c3ccccc3)NC(=O)c(cc4)c(OC)cc4OC |
| **316** | CN(C)C(=N/[H])\c(cc1)ccc1C(=O)Nc(cc2)c(cc2Cl)C(=O)Nc(nc3)ccc3Cl |
| **322** | Cc1cc(Cl)c(c(Cl)c1)OCCOc2ccc(cc2)[C@H]3CCNC[C@@H]3C(=O)N(C4CC4)Cc(c5)cc(CCCOC)cc5OCC6(CC#N)CC6 |
| **323** | Cc1cc(Cl)c(c(Cl)c1)OCCOc2ccc(cc2)[C@H]3CCNC[C@@H]3C(=O)N(C4CC4)Cc(c5)cc(CCCOC)cc5OCCOC |
| **325** | Cc1cc(Cl)c(c(Cl)c1)OCCOc2ccc(cc2)[C@H]3CCNC[C@@H]3C(=O)N(C4CC4)Cc(c5)cc(CCCOC)cc5OCCCNS(=O)(=O)C |
| **326** | Cc1cc(Cl)c(c(Cl)c1)OCCOc2ccc(cc2)[C@H]3CCNC[C@@H]3C(=O)N(C4CC4)Cc(c5)cc(CCCOC)cc5OCCCNC(=O)NCC |
| **327** | Cc1cc(Cl)c(c(Cl)c1)OCCOc2ccc(cc2)[C@H]3CCNC[C@@H]3C(=O)N(C4CC4)Cc(c5)cc(CCCOC)cc5OCC(C)(C)O |
| **328** | Cc1cc(Cl)c(c(Cl)c1)OCCOc2ccc(cc2)[C@H]3CCNC[C@@H]3C(=O)N(C4CC4)Cc(c5)cc(CCCOC)cc5OC[C@@H]6C[C@@H]6C(=O)OCC |
| **384** | COc1cc(N)c(Cl)cc1C(=O)N[C@H]2CCN(C[C@H]2OC)CCCOc(cc3)ccc3F **Cisapride** |
| **395** | c1ccccc1C(O)(c2ccccc2)C(CC3)CCN3CCC[C@H](O)c(cc4)ccc4C(C)(C)C **Terfenadine** |
| **398** | Clc1cnc(cc1)NC(=O)c2c(cccc2)NC(=O)c(cc3)ccc3/C(=N/[H])N(C)C |
| **399** | Clc1cnc(cc1)NC(=O)c2c(ccc(Cl)c2)NC(=O)c(cc3)ccc3/C(=N/[H])N(C)C |
| **411** | c1cc(Cl)ccc1-c2nc(no2)COc(cc3)cc(c34)[C@H](CC4)N(CC5)CCN5C(C)C |
| **422** | c1cccc(c12)snc2N(CC3)CCN3CCc(c4)ccc(c45)CC[C@@H]5NC(=O)C(F)(F)F |
| **423** | c1cccc(c12)c(ns2)N(CC3)CCN3CCc(c4)ccc(c45)[C@H](CC5)NC(=O)C(F)(F)F |
| **428** | c1cccc(c12)snc2N(CC3)CCN3CCc(c4)ccc(c45)CC[C@@H]5NC(=O)C6CC6 |
| **433** | c1cccc(c12)snc2N(CC3)CCN3CCc(c4)ccc(c45)CC[C@@H]5NC(=O)c(cc6)ccc6F |
| **437** | c1cccc(c12)snc2N(CC3)CCN3CCc(c4)ccc(c45)CC[C@@H]5NC(=O)c6ccco6 |
| **438** | c1cccc(c12)snc2N(CC3)CCN3CCc(c4)ccc(c45)CC[C@@H]5NC(=O)c6ccno6 |
| **442** | c1cccc(c12)c(ns2)N(CC3)CCN3CCc(c4)ccc(c45)[C@H](CC5)NC(=O)C6CC6 |
| **444** | c1cccc(c12)c(ns2)N(CC3)CCN3CCc(c4)ccc(c45)[C@H](CC5)NC(=O)c(cc6)ccc6F |
| **460** | CCCc1nc(C#N)nc(c1)-c2cc(C(F)(F)F)cc(c2)C(=O)N(CCN(C)C)Cc3ccccc3 |
| **528** | N#Cc1ccc(cc1)-c(cc2)cc(c23)ccc(c3)CCN4[C@H](C)CCC |
| **531** | C1CC[C@@H](C)N1[C@@H](C2)C[C@H]2c(cc3)ccc3-c(cc4)ccc4C#N |
| **543** | n1c(C)ccc(c12)c(ccc2)N(CC3)CCN3CCc4cccc(c45)n6c(CO5)nc(c6)C(F)(F)F |
| **573** | c1cccc(c12)[nH]c(-c3ccccc3)c2C4CCN(CC4)CCc5ccccc5 |
| **574** | c1cccc(c12)[nH]c(-c3ccccc3)c2[C@@H](C[C@H]45)C[C@H](CC5)N4CCc6ccccc6 |
| **585** | C1NCC[C@@H](F)[C@@H]1c2c([nH]c(c23)cc(F)cc3)-c4cccc(c45)cccc5 |
| **594** | c1cc([N+](=O)[O-])ccc1OCCNCCc(cc2)ccc2[N+](=O)[O-] |
| **595** | c1cc([N+](=O)[O-])ccc1OCCN(C)CCc(cc2)ccc2[N+](=O)[O-] |
| **596** | c1cc([N+](=O)[O-])ccc1OCCN(CC)CCc(cc2)ccc2[N+](=O)[O-] |
| **597** | c1cc([N+](=O)[O-])ccc1OCCN(CCF)CCc(cc2)ccc2[N+](=O)[O-] |
| **602** | c1cc([N+](=O)[O-])ccc1OCCCCN(C)CCc(cc2)ccc2[N+](=O)[O-] |
| **603** | c1cc([N+](=O)[O-])ccc1OCCCCN(C)Cc(cc2)ccc2[N+](=O)[O-] |
| **606** | c1cc(Cl)ccc1C(=O)C2CCN(CC2)CCc(cc3Cl)ccc3Cl |
| **607** | c1cc(C)ccc1C(=O)C2CCN(CC2)CCCc3ccccc3 |
| **609** | c1cc(Cl)ccc1C(=O)[C@H](CC2)CC[N@@+]2(C)CCc3ccccc3 |
| **610** | CCCCCCCN(CC)CCCCc(cc1)ccc1Cl |
| **611** | CCCCCCCN(CC)C/C=C/Cc1ccccc1 |
| **614** | CCCCCCCN(CC)CC#CC(c1ccccc1)c2ccccc2 |
| **615** | CCCCCCC[N+](CC)(CC)CC#Cc(cc1)ccc1Cl |
| **617** | CCCCCCc([n+]1C)cccc1CCCCCC |
| **618** | CCCCCCCc([n+]1C)cccc1CCCCCCC |
| **619** | CCCCCCCCc([n+]1C)cccc1CCCCCCCC |
| **620** | CCCCCCCCCc([n+]1C)cccc1CCCCCCCCC |
| **621** | CCCCCCCCCCc([n+]1C)cccc1CCCCCCCCCC |
| **622** | CCCCCCCCCCCCc([n+]1C)cccc1CCCCCCCCCCCC |
| **625** | Clc1ccc(cc1)OCCCc([n+]2C)cccc2CCCOc(cc3)ccc3Cl |
| **627** | c1ccccc1-c2ccc(cc2)CCc([n+]3C)cccc3CCc4ccc(cc4)-c5ccccc5 |
| **630** | c1ccccc1-c2ccc(cc2)C#Cc([n+]3C)cccc3C#Cc(cc4)ccc4-c5ccccc5 |
| **632** | c1c(C)cccc1-c2ccc(cc2)C#Cc([n+]3C)cccc3C#Cc(cc4)ccc4-c5cccc(c5)C |
| **634** | c1c(Cl)cccc1-c2ccc(cc2)C#Cc([n+]3C)cccc3C#Cc(cc4)ccc4-c5cccc(Cl)c5 |
| **638** | Clc1ccc(cc1)-c2ccc(cc2)C#Cc([n+]3C)cccc3C#Cc(cc4)ccc4-c(cc5)ccc5Cl |
| **639** | c1cccc(c12)n(c(n2)C)[C@H](C3)C[C@@H](N([C@@H]34)C)COC4 |
| **655** | c1cc(Cl)ccc1Oc(cc2)ccc2Nc3nccc(c3)N |
| **664** | FC(F)(F)Oc(cc1)ccc1Oc(cc2)ccc2Nc3nccc(n3)N |
| **670** | n1c(C)ccc(c12)c(ccc2)N3CCN(CC3)CCc(c4)cccc4NC(=O)c5ccccc5 |
| **678** | CC(C)(C)C#Cc(cc1)cc2c1Oc(c3[C@]24N=C(N)OC4)ccc(c3)-c5cncnc5 |
| **683** | c1ncncc1-c(c2)ccc(c2[C@]34N=C(N)OC4)Oc5c3cc(cc5)OCC6CCCCC6 |
| **690** | CC(C)(C)COc(cc1)cc2c1Oc(c3[C@]24N=C(N)OC4)ccc(c3)-c5cncc(c5)C#N |
