# Supplementary material for: Computational Tool for Fast in silico Evaluation of hERG K+ Channel Affinity
Source: Front Chem. 2017 Feb 23;5:7. doi: 10.3389/fchem.2017.00007 (PMC5408157; doi:10.3389/fchem.2017.00007)
Supplement: Supplementary file 4 [file Image1.PDF]

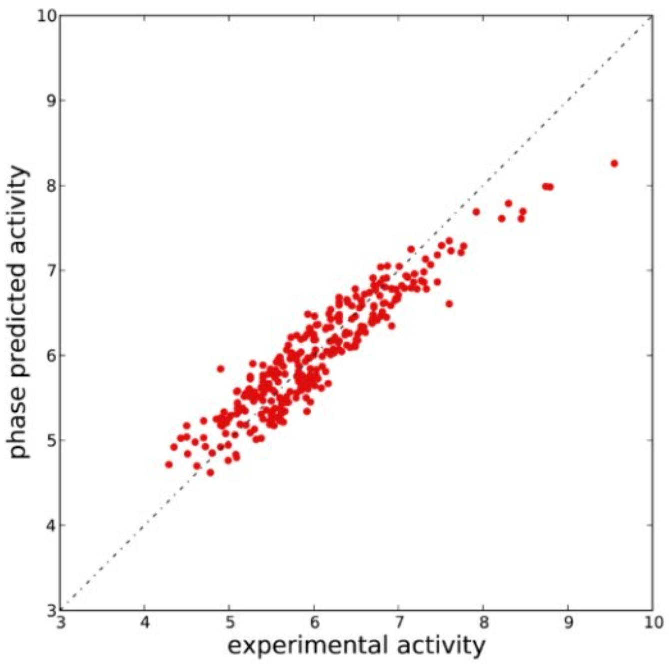

Figure S1. Scatter plot for the predicted and observed p*Ki* values (M) as calculated by the 3D-QSAR model applied to the external test set.
